# New plant immunity elicitors from a sugar beet byproduct protect wheat against *Zymoseptoria tritici*

**DOI:** 10.1038/s41598-022-26800-z

**Published:** 2023-01-03

**Authors:** Samara Mejri, Alina Ghinet, Maryline Magnin-Robert, Béatrice Randoux, Cristina-Maria Abuhaie, Benoit Tisserant, Philippe Gautret, Benoit Rigo, Patrice Halama, Philippe Reignault, Ali Siah

**Affiliations:** 1 Joint Research Unit 1158 BioEcoAgro, Junia, Université de Lille, Université de Liège, UPJV, ULCO, INRAE, Université d’Artois, 59000 Lille, France; 2 Sustainable Chemistry Team, Laboratory of Sustainable Chemistry and Health, Junia, Health & Environment Department, 59000 Lille, France; 3 Institut Pasteur Lille, U1167 - RID-AGE - Facteurs de Risque Et Déterminants Moléculaires Des Maladies Liées Au Vieillissement, University Lille, Inserm, CHU Lille, 59000 Lille, France; 4 Faculty of Chemistry, University of Iasi, Boulevard Carol I, Nr. 11, 700506 Iasi, Romania; 5 Unité de Chimie Environnementale et Interactions sur le Vivant (EA 4492), Université du Littoral Côte d’Opale, Calais, France

**Keywords:** Chemical biology, Plant sciences

## Abstract

The current worldwide context promoting agroecology and green agriculture require the discovery of new ecofriendly and sustainable plant protection tools. Plant resistance inducers, called also elicitors, are one of the most promising alternatives fitting with such requirements. We produced here a set of 30 molecules from pyroglutamic acid, bio-sourced from sugar beet byproducts, and examined for their biological activity on the major agro-economically pathosystem wheat-*Zymoseptoria tritici*. Foliar application of the molecules provided significant protection rates (up to 63% disease severity reduction) for 16 among them. Structure–activity relationship analysis highlighted the importance of all chemical groups of the pharmacophore in the bioactivity of the molecules. Further investigations using in vitro and *in planta* antifungal bioassays as well as plant molecular biomarkers revealed that the activity of the molecules did not rely on direct biocide activity towards the pathogen, but rather on the activation of plant defense mechanisms dependent on lipoxygenase, phenylalanine ammonia-lyase, peroxidase, and pathogenesis-related protein pathways. This study reports a new family of bio-sourced resistance inducers and provides new insights into the valorization of agro-resources to develop the sustainable agriculture of tomorrow.

## Introduction

Septoria tritici blotch (STB), caused by the fungal pathogen *Zymoseptoria tritici*, is one of the most challenging diseases on wheat crops worldwide. *Z. tritici* is an hemibiotrophic fungus with a symptomless biotrophic phase of approximatively two weeks, followed by a necrotrophic phase of around one week^1^. However, these durations may vary depending on host resistance, fungal pathogenicity, and environmental conditions^2,3^. Leaf penetration by the fungal spore-emerging germ tubes takes place through the stomatal apertures during the early stages of infection^4^. This passive mode of penetration avoids the need for specialized penetration structures such as appressoria, although *Z. tritici* develops appressorium-like structures on the leaf surface^1^. This lack of appressoria in *Z. tritici* is associated with an absence in this pathogen of several key genes required for appressorium formation^5^. Avoiding the differentiation of such active invasion structures may help the pathogen to escape the plant immunity responses associated with direct physical penetrations^6^. During the biotrophic phase, the fungus grows between the mesophyll cells and survives by feeding on the apoplastic nutrients^7^. During the transition to the necrotrophic phase, the pathogen produces an arsenal of toxins and cell-wall degrading enzymes to kill host cells and releases the nutrients from leaf tissues^1,8^. The establishment of the necrotrophic phase results in typical STB symptoms, corresponding to visible chlorotic and/or necrotic lesions containing dark brown to black sporulating bodies called pycnidia^6,7^. Pycnidia contains pycnidiospores, the fungal asexual spores, that are responsible for the spread of the disease up through the host leaf layers, due to their ability to travel by splash dispersal^6^. Management of STB relies mainly on the use of conventional fungicides, and to lesser extent on resistant cultivars. In the European Union where the climate is suitable for disease development, about 70 % of marketed fungicides target STB^9^, and the cost of fungicide application due to this disease is about 1.2 billion euros in France, Germany and the United Kingdom considered altogether^10^. Such chemical inputs are increasingly controversial because of their potential hazardous effects on both the environment and human health. Moreover, their intensive use generated in the field the development of fungicide resistance in several fungal populations around the world, leading to a decrease in their efficacy^11,12^. This resistance development could be facilitated by the high fitness degree of *Z. tritici*, resulting very likely from its active sexual reproduction and genetic recombination in the field^13^. Hence, looking for disease control alternatives such as the use of resistance inducers, also referred to as elicitors, is strongly encouraged in order to promote sustainable agriculture and safer food.

Elicitors are agents that confer improved protection to plants further pathogen or pest attacks by triggering host plant immunity. The mode of action of these compounds differs from that of traditional fungicides because they do not target directly the bio-aggressor through antifungal activity, but they inhibit its development indirectly *via* the elicitation of plant defense reactions^14^. The concept of elicitor timepoints back to the 1970s, when microbial molecules able to induce in the plant the production of phytoalexins, secondary metabolites with antimicrobial activity, were identified^15^. The subsequent use of such molecules in practice by the early 1980s led to the emergence of the concept of resistance inducers^16^. Elicitors can be released during plant-microbe interactions, and their recognition by the plant is ensured by pattern recognition receptors (PRRs) occurring in the plant cytoplasmic membrane^17^. Overall, there are three categories of elicitors, considered according to their origin during plant-microbe interactions: (i) pathogen-associated molecular pattern (PAMP) from pathogenic microorganisms, (ii) microbe-associated molecular patterns (MAMPs) from non-pathogenic microorganisms, both considered as exogenous elicitors, and (iii) damage-associated molecular pattern (DAMP) from the plant itself, corresponding to endogenous elicitors^14^. Most of commercial elicitors are compounds that mimic such molecules and their application on the plant can activate several defense mechanisms, including ion fluxes across the plasma membrane, reactive oxygen species (ROS) metabolism, pathogenesis related (PR)-protein and phytoalexin syntheses, and phenylpropanoid and octadecanoid pathways^18,19^. Such defense reactions are effective against a wide range of crop enemies, including viruses, bacteria, fungi, oomycetes, nematodes, as well as herbivores^14^.

Elicitors can be of natural or synthetic origin. Natural elicitors include living microorganisms, plant extracts, microbial cell-wall extracts, microbial metabolites, minerals, and ions, while synthetic elicitors include mainly structural or functional analogues of plant hormones^20^. One major limitation for the development of natural elicitors is the availability of resources (raw materials) required for the production of quantities of products sufficient for the treatment of large crop surfaces. Food industry byproducts could be an environmentally friendly bioresource for the conception of plant protection compounds, including elicitors. For instance, extracts from grape marc, which is a winemaking byproduct, have been demonstrated to induce defense reactions in tobacco, *Arabidopsis*, and tomato^21,22^. Byproducts from sugar beet agroindustry, available in great quantities^23^, can also provide biomolecules with high biological activity, but they have never been explored for their potential as a bioresource for the development of plant protection products. During the process of sugar beet transformation, several byproducts are released, including molasses, which represent the syrup (raw juice) obtained from sugar beet roots after sucrose extraction^24^. Molasses contain around 30% of amino acids, including glutamine and glutamic acid, which represent more than 50% of total sugar beet amino acids^25^. Partial decomposition or thermal cyclisation of glutamine leads to the formation of pyroglutamic acid (PGA), also known as pidolic acid or as “the forgotten amino acid^26^”. PGA represents an affordable raw material, without toxicity and consisting of interesting functionalizable units (lactam and carboxylic acid groups), allowing the creation of new biosourced molecules^27^. This molecule has also been isolated recently from *Disporopsis aspersa*, a chinese plant from the *Asparagaceae* family^28^. Numerous proteins carrying PGA at the *N*-terminus, such as PR proteins, are known for their implication in plant defense reactions^29^. Nevertheless, the feature of PGA residue for these proteins is not completely understood.

PGA is a constrained analogue of γ-aminobutyric acid (GABA), a non-proteinogenic amino acid synthesized from L-glutamate in a reaction catalyzed by glutamate decarboxylase^30^. *β*-Aminobutyric acid (BABA), an isomer of GABA, is one of the most studied elicitors and has been shown to induce plant resistance against a wide range of pathogens and pests, including STB^14,31^. BABA is known to possess great potential as a priming agent of defense reactions that are controlled by salicylic acid-dependent and -independent signaling pathways^32^. Salicylic acid-independent signaling pathways are defense pathways relying on other signalization pathways such jasmonic acid-dependent pathway. Regarding PGA, this molecule and its derivatives have never been explored for their activity in plant protection. On the other hand, there is a lack of discovery for the identification of new elicitors; and regarding *Z. tritici*, only few reports have been launched on the effectiveness of plant resistance inducers towards this agro-economically important pathogen^33–36^. The objective of the current study was thus to identify new elicitors, biosourced from PGA, for the control of *Z. tritici*. After an identification of a molecule (M1, obtained from PGA) with a significant protection efficacy towards the pathogen, a set of 28 other molecules (M2 to M29, functionalized from M1) were obtained and assessed again for their protection efficacy against the fungus. The analogue of PGA, GABA, was also included in the experiments. A structure-activity relationship analysis was then undertaken to highlight the molecule chemical groups responsible for the activity. Furthermore, two molecules with the highest protection efficacy levels (M1 and M2), as well as GABA, were selected and characterized for their modes of action within the plant in order to elucidate defense pathways involved in the observed plant induced resistance.

## Results

### Production of functionalized elicitors

**Figure 1:**
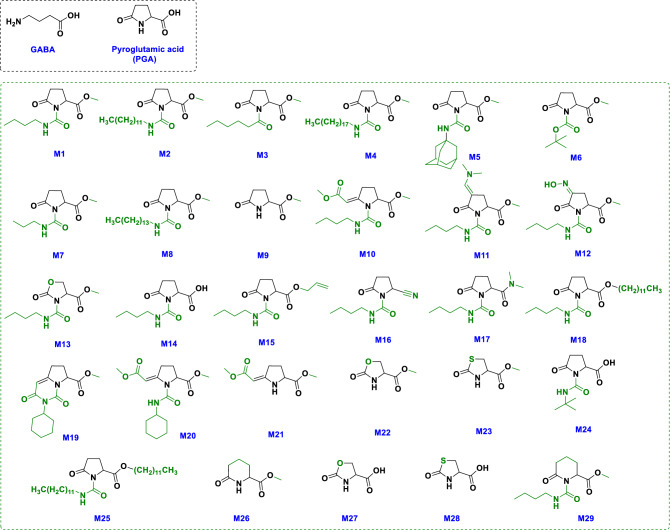
Chemical structure of γ-aminobutyric acid (GABA), pyroglutamic acid (PGA), the molecule M1 derived from PGA, and the 28 molecules (from M2 to M29) derived from M1.

**Figure 2:**
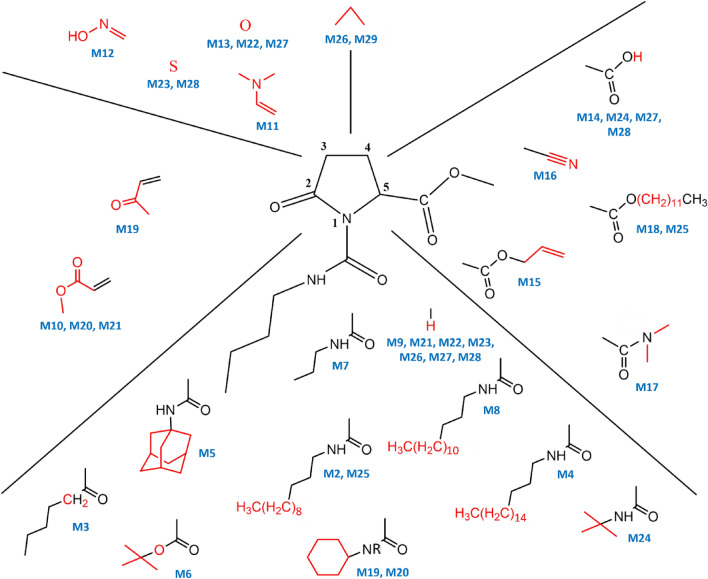
Pharmacophore of the molecule M1, showing the structure modifications performed on the different positions of the main nucleus of M1. All changes were done separately on all positions of the nucleus, except on position 4. Some molecules were modified in several positions simultaneously.

A panel of 29 molecules, including M1 (derived from PGA that was originating from sugar beet molasses) as well as 28 molecules functionalized from M1, were obtained *via* well-known synthetic procedures and following the twelve principles of green chemistry introduced in the USA during the 1990s^37,38^ (Fig. 1, Supplementary Fig. S1). The molecules were obtained using different chemical transformations, including: chain extension, isocyanate addition on lactam nitrogen, saponification, esterification, cyclization, ring extension, synthesis of vinylogous acylureas or substitution on the lactam nitrogen (Fig. 2, Table 1). All modifications (functionalization) were performed separately on all positions of the main nucleus of M1 (Fig. 2), except position 4, because modifications on this position are multi-step reactions and consequently costly, time consuming and discordant with the green chemistry principles and targeted application. The novel synthesized molecules showed different levels of lipophilic (hydrophobic) patterns, as revealed by Log *P* values (Table 1). Log *P*, also known as Log Kow, is a measure of the differential solubility of chemical compounds in two solvents (octanol/water partition coefficient); the more the log *P* is high, the more the corresponding molecule is lipophilic.

### GABA, M1 and 15 functionalized molecules from M1 protect wheat against *Z. tritici*

GABA, PGA, M1 (derived from PGA), and a panel of 28 molecules functionalized from M1 through green chemistry principles, were tested for their protection efficacy on wheat against *Z. tritici* in semi-controlled conditions (greenhouse). High levels of disease extent were observed on control plants at 21 dpi. Indeed, rates of disease severity (percentage of leaf area with lesions bearing pycnidia) reached 57 % and 55 % on inoculated plants treated with water alone or with water supplemented with the wetting agent Heliosol, respectively (Table 1). Statistical analyses showed no significant difference among these two controls, indicating that Heliosol did not confer any protection against the pathogen. Likewise, PGA, with 62% of diseased leaf area, did not show any protection against *Z. tritici* (Table 1). However, GABA, M1 and 15 other derived molecules allowed significant disease reductions, ranging from 19 to 63 % when compared to inoculated non-treated control plants. The thirteen remaining molecules did not display any protection on the corresponding treated plants. M1, M2 and GABA are among the molecules showing the best protective rates against the fungus (Table 1).

**Table 1:** Protection efficacy at 5 mM of **γ-**aminobutyric acid (GABA), pyroglutamic acid (PGA), M1 (derived from PGA) and 28 molecules functionalized from M1 on bread wheat (cultivar Alixan) against *Zymoseptoria tritici* (strain T02596), and description of the chemical modifications performed on M1 to obtain the functionalized molecules.

Molecule	Modifications compared to M1^a^	Modified position					Log *P*^b^	Disease severity (%)^c^	Protection efficacy^d^
		1	2	3	4	5			
Water-treated control								57 ± 0.15	0
Heliosol-treated control								55 ± 0.13	4
GABA							− 0.64 ± 0.22	**31 ± 0.15**	**44**
PGA							− 2.39 ± 0.27	62 ± 0.12	0
M1		–	–	–	–	–	− 0.70 ± 0.62	**26 ± 0.13**	**55**
M2	C8 chain extension	X	–	–	–	–	+ 3.55 ± 0.62	**21 ± 0.13**	**63**
M3	Nitrogen replacement	X	–	–	–	–	+ 1.71 ± 0.26	**34 ± 0.16**	**40**
M4	C14 chain extension	X	–	–	–	–	+ 6.73 ± 0.62	**35 ± 0.14**	**39**
M5	Bulky unit	X	–	–	–	–	− 0.58 ± 0.63	**39 ± 0.12**	**31**
M6	Chain ramification + Nitrogen replacement	X	–	–	–	–	− 0.05 ± 0.60	**42 ± 0.10**	**26**
M7	C1 chain suppression	X	–	–	–	–	− 1.24 ± 0.65	**44 ± 0.13**	**23**
M8	C10 chain extension	X	–	–	–	–	+ 4.61 ± 0.62	49 ± 0.10	14
M9	Chain suppression	X	–	–	–	–	− 1.92 ± 0.28	61 ± 0.13	0
M10	Vinylogous	–	X	–	–	–	+ 0.70 ± 0.61	56 ± 0.12	1
M11	Enamine	–	–	X	–	–	+ 0.94 ± 0.65	**38 ± 0.12**	**33**
M12	Oxime addition	–	–	X	–	–	− 0.38 ± 0.66	47 ± 0.16	17
M13	C replacement	–	–	X	–	–	− 0.20 ± 0.65	50 ± 0.10	12
M14	Ester saponification	–	–	–	–	X	− 1.24 ± 0.62	**33 ± 0.11**	**41**
M15	C2 ester chain extension	–	–	–	–	X	+ 0.16 ± 0.63	**36 ± 0.14**	**36**
M16	Ester replacement	–	–	–	–	X	− 1.03 ± 0.62	**39 ± 0.15**	**30**
M17	Ester replacement	–	–	–	–	X	− 1.63 ± 0.63	49 ± 0.16	13
M18	C11 ester chain extension	–	–	–	–	X	+ 5.14 ± 0.62	58 ± 0.09	0
M19	Bulky (1) + vinylogous (2)	X	X	–	–	–	+ 2.62 ± 0.40	**31 ± 0.15**	**44**
M20	Bulky (1) + vinylogous (2)	X	X	–	–	–	− 1.63 ± 0.63	**37 ± 0.11**	**35**
M21	Chain suppression (1) + vinylogous (2)	X	X	–	–	–	− 0.06 ± 0.29	46 ± 0.18	18
M22	Chain suppression (1) + C replacement (3)	X	–	X	–	–	− 0.33 ± 0.31	**41 ± 0.21**	**28**
M23	Chain suppression (1) + C replacement (3)	X	–	X	–	–	− 0.65 ± 0.71	**46 ± 0.16**	**19**
M24	Chain ramification (1) + Ester saponification (5)	X	–	–	–	X	− 1.53 ± 0.62	**39 ± 0.12**	**31**
M25	C8 chain (1) + C11 ester chain extensions (5)	X	–	–	–	X	+ 9.39 ± 0.62	48 ± 0.16	16
M26	Suppression of the substituent (1) + 1 C ring extension (3 + 4)	X	–	X	X	–	− 1.36 ± 0.28	51 ± 0.14	11
M27	Chain suppression (1) + C replacement (2) + Saponification (3)	X	–	X	–	X	− 0.79 ± 0.30	48 ± 0.15	16
M28	Chain suppression (1) + C replacement (2) + Saponification (3)	X	–	X	–	X	− 1.11 ± 0.71	63 ± 0.09	0
M29	C1 ring extension (3 + 4)	–	–	X	X	–	− 0.14 ± 0.62	57 ± 0.11	0

^a^When more than one modification was performed, their positions on M1 are mentioned between brackets.

^b^Calculated with ACD/Log *P* software. ± indicates standard deviation.

^c,d^Disease severity (percentage of leaf area with lesions bearing pycnidia) and protection values highlighted in bold are significantly different from the water-treated control according to the Tukey test at *P* = 0.05. A total of 36 third leaves were used as replicates in each treatment and the control. ± indicates standard deviation.

### Functionalized molecules did not exhibit any direct antifungal effect

All molecules were assessed in vitro for their direct activity towards *Z. tritici*. In vitro bioassays performed on microplates revealed that none of the tested molecules exhibits direct antifungal effect towards *Z. tritici*, since non-significant difference in the rates of fungal growth was observed among treated and non-treated conditions (Supplementary Fig. S2). Nevertheless, the wetting agent Heliosol completely inhibited the in vitro mycelial growth of the fungus at the approved field concentration (0.2%), while the other wetting agent Cantor (used for further experiments regarding cytological and molecular investigations) did not show any significant effect on the fungal growth at the approved field concentration (0.15%).

### Structure–activity relationship analysis

A structure-activity relationship investigation was performed in order to identify the chemical groups responsible for the activity of the molecule M1. All modifications (functionalizations) were done separately on all positions of the main nucleus of M1 (Fig. 2), except position 4 that involves multi-step chemistry unfavorable to potential agricultural use. Observations of the protective effects of the molecules (Table 1) and their structural features (summarized in Fig. 2 and Table 1) have highlighted some regular outlines of structure-activity relationships. Most of the modifications carried out on M1 stabilize, reduce or abolish the activity, except the modification performed in position 1 generating the molecule M2 (carboxylic chain extension to C8) which enhanced the activity from 55% to 63% disease reduction (Table 1, Fig. 2). Cyclisation of the carboxylic chain (M5) allowed to the molecule M1 to keep a significant efficacy, while the suppression, shortening or extension of the carboxylic chain, over 12 carbons (molecules M9, M7, M8, and M4), led to a decrease in the activity. This decline of activity is also observed when the nitrogen group is substituted by a carbon atom in position 1 (M3). The introduction of a vinylogous group in position 2 (M10) induced a complete suppression of the activity. All modifications made on position 3, such as the substitution of the carbon by an oxygen, addition of an oxime or an enamine (M13, M12 and M11), led to a decrease in the activity. Finally, changes made on position 5 confirmed the importance of the methyl ester since saponification, extension, and replacement decreased the activity (M14, M15, and M16) or inhibited it completely (M18). Some modifications were made simultaneously on two or three different positions (Fig. 2, Table 1). The molecules modulated on two different positions showed whether a stabilized, reduced (M19, M20, M22, M23, and M24) or no plant protection activity (M21 and M25). Nevertheless, the simultaneous modifications on three different positions in molecules M26, M27, M28 and M29 abolished the biological activity. In order to examine the effect of the lipophilic property of the molecules on their activity, a correlative analysis between the log *P* values of the molecules and their protection efficacy levels (Table 1) was performed using the Pearson test. Results revealed a lack of correlation between these two parameters (r = 0.20, *P* value = 0.20), indicating that the lipophilic property did not explain the activity of the molecules.

### M1, M2 and GABA confer the best protective rates

**Figure 3:**
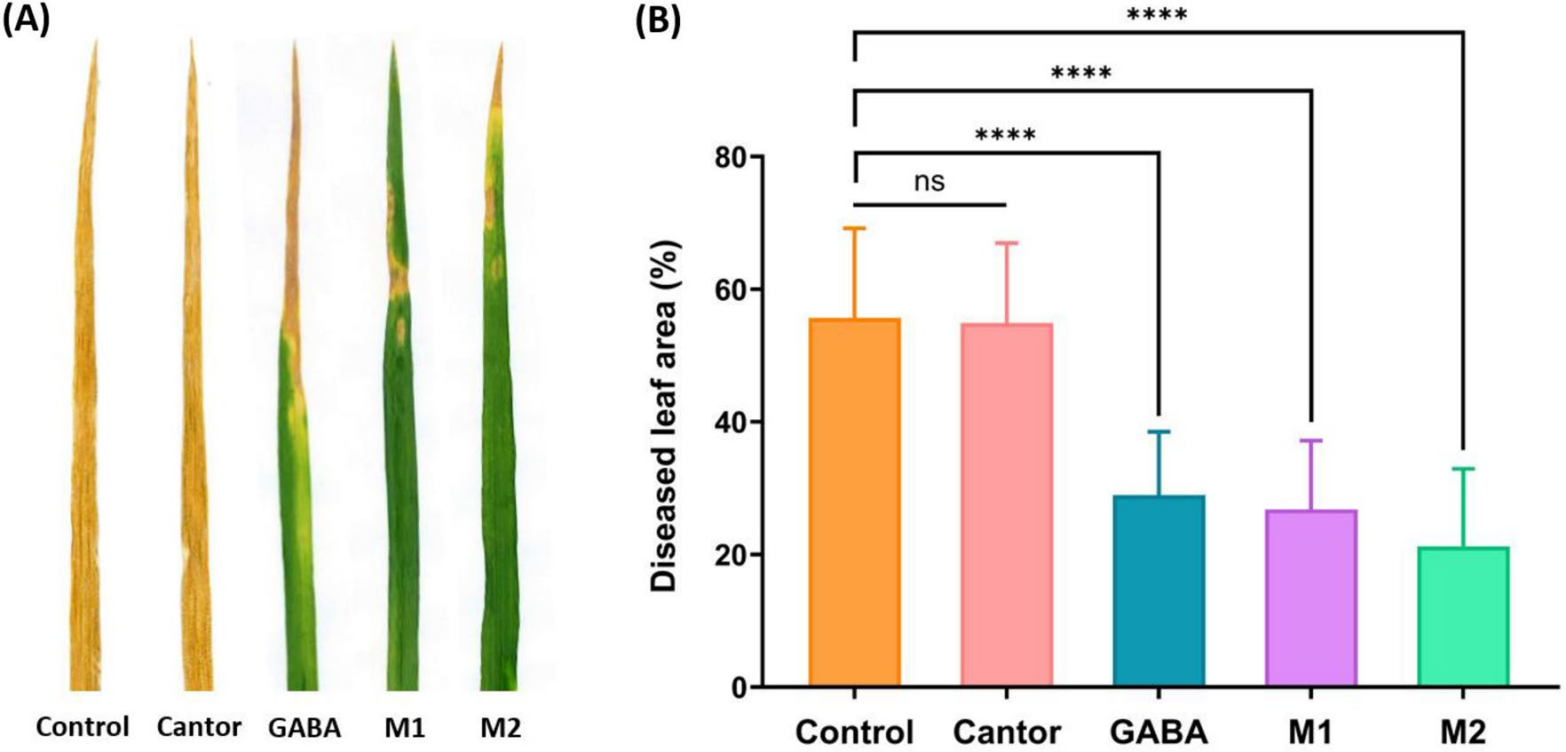
Disease severity on wheat plants (cultivar Alixan) against *Zymoseptoria tritici* (strain T02596) treated with water (control), Cantor (0.15%), **γ-**aminobutyric acid (GABA), and M1 and M2 molecules at 5 mM. **A** stands for representative third leaves from the control and each treatment, while **B** strands for the means scored on 36 third leaves from the control and each treatment. Disease symptoms (in A and B) were recorded 21 days post-inoculation by scoring the percentage of the third leaf area covered with lesions bearing pycnidia. Asterisks indicate significant differences according to the Tukey test at *P* = 0.001. Bars indicate standard deviation (SD).

As revealed in the in vitro bioassay, Heliosol displayed a direct effect on *Z. tritici* development on PDA medium, although this wetting agent did not protect the plants against the disease under greenhouse conditions. Therefore, for the following experiments, Heliosol was substituted by the other wetting agent Cantor, deprived of any direct antifungal activity according to the in vitro bioassays. Furthermore, an additional experiment in the greenhouse carried out using Cantor (0.15 %) as a wetting agent confirmed that GABA, M1 and M2 significantly reduced disease extent (29 %, 27 % and 21 % of diseased leaf area, respectively) on the plants treated with these molecules, compared to plants treated with water alone (56 % of diseased leaf area) (Fig. 3). Disease severity reductions by GABA, M1 and M2 treatments reached 48 %, 52 % and 63 %, respectively. The ranking of the three molecules according to their protective effect remained unchanged when compared to the previous assay dedicated to the structure-activity relationship analysis (Fig. 3, Table 1). No significant difference was obtained between the disease extent scored on the plants treated with Cantor (55% of diseased leaf area) and that recorded on control plants treated with water alone (Fig. 3).

### M1, M2 and GABA did not affect *in planta* epiphytic growth of *Z. tritici*

**Figure 4:**
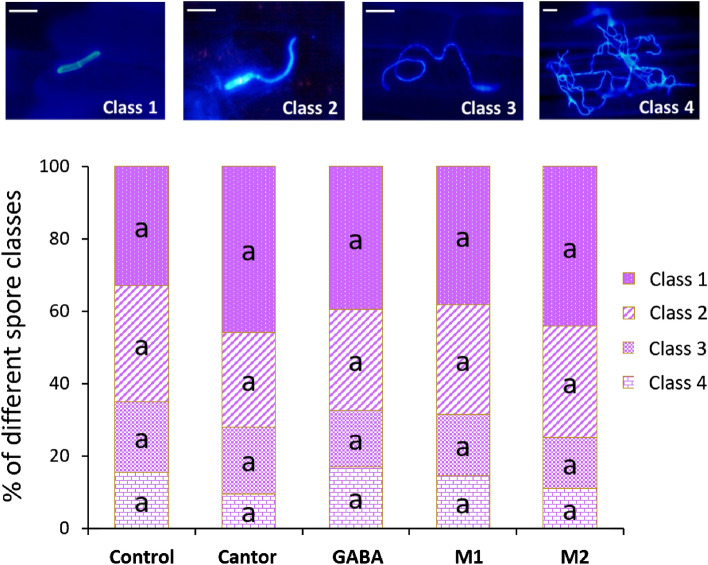
*In planta* effect of **γ-**aminobutyric acid (GABA), M1, M2 and Cantor treatments on the epiphytic hyphal growth of *Zymoseptoria tritici* (strain T02596) on the wheat leaves (cultivar Alixan), compared to the water-treated control. Three third-leaf segments with 100 fungal spores monitored on each were used as replicates in each condition. Four different classes of Calcofluor-stained germinated spores were assessed five days post-inoculation (class 1, non-geminated spores; Class 2, geminated spores with small germ tube; class 3, geminated spores with developed germ tube; class 4, geminated spores with a strongly developed germ tube). Within each class, bars with common letters are not significantly different using the Tukey test at *P* = 0.05. Scale bar = 10 µ.

*In planta* bioassays were undertaken in order to evaluate the effect of M1, M2 and GABA, as well as the wetting agent Cantor, on the epiphytic hyphal growth of *Z. tritici*. Cytological observations carried out at 1 dpi showed no significant difference among the rates of germinated spores recorded on the control plants inoculated and treated with water alone and on the plants inoculated and treated with Cantor, GABA, M1 or M2. Indeed, the rates of germinated spores at 1 dpi consisted of 74 %, 82 %, 83 %, 87 %, and 82 % for control, Cantor, GABA, M1, and M2 treatments, respectively, without any significant difference among these values according to the Tukey test at *P* = 0.05. Likewise, no effect of the treatments with Cantor, or with GABA, M1 or M2, on the epiphytic (on leaf surface) hyphal development of the pathogen was observed at 5 dpi. Indeed, no significant difference was detected among all conditions regarding the different assessed classes of mycelial growth (Fig. 4). Overall, fungal germ tubes at 5 dpi were randomly oriented on the leaf surface and the patterns of mycelial growth were similar in both plants inoculated and treated with water alone and plants inoculated and treated with Cantor, GABA, M1 or M2 (*data not shown*).

### M1, M2 and GABA induce defense-related mechanisms in wheat

The expression level of four genes involved in plant defense reactions (*LOX*, *PAL*, *POX2*, and *PR1*) were assessed during the early stages of treatment as well as infection in order to evaluate the elicitation and priming effects of M1, M2 and GABA on wheat plants against *Z. tritici*.

**Figure 5:**
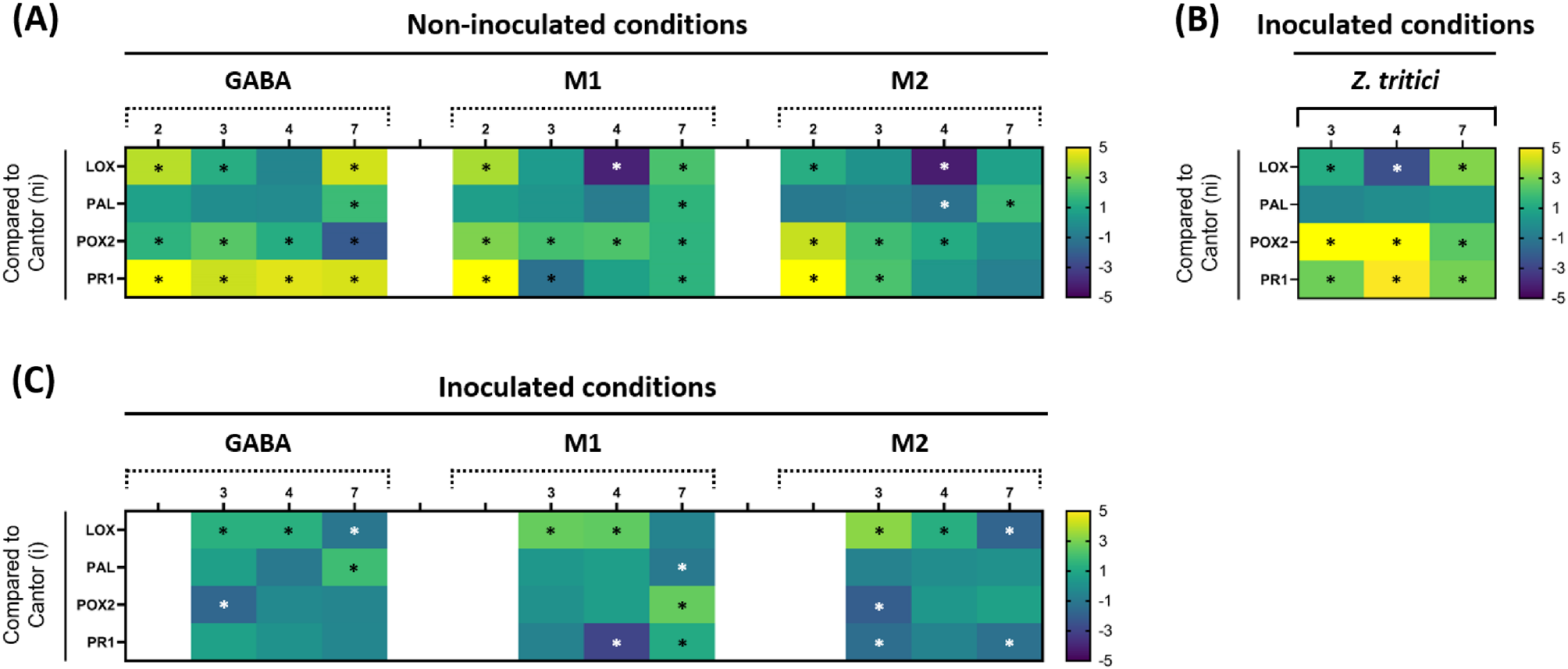
Relative expression of four selected defense-related genes in wheat leaves (cultivar Alixan) treated or not with **γ-**aminobutyric acid GABA, M1 and M2 and inoculated or not with *Zymoseptoria tritici* (strain T02596). (**A**) non-infectious conditions (ni) where GABA, M1 and M2-treated and non-inoculated plants are compared to Cantor-treated and non-inoculated plants; (**B**) Cantor-treated and inoculated plants compared to Cantor-treated and non-inoculated control plants; (**C**) GABA-, M-1 and M2-treated and inoculated plants compared to Cantor-treated and inoculated control plants. The data are resulting from two independent experiments, with three biological replicates and two technical replicates (six replicates) each. Values of relative gene expression were converted to Log 2 and represented in the form of a heatmap. Over-expression is highlighted with shades of green to yellow, while down-expression is presented with shades of purple to blue. The genes were considered significantly down-regulated (white asterisks) or up-regulated (black asterisks) compared to the corresponding controls when changes in their expression were < 0.5 × or > 2 x, respectively. The numbers on the heatmap indicate days post-treatment (dpt).

In non-infectious conditions, gene expression profiles in plants non-inoculated and treated with M1, M2 and GABA were compared to plants non-inoculated and treated with water amended with 1% ethanol and 0.15% Cantor (Fig. 5A). *LOX* gene was induced by 2 dpt by all the three molecules, with induction levels of 15-, 13- and 2- folds for plants treated with GABA, M1 and M2, respectively. GABA-treated plants were the only ones to show a slight up-regulation of 2-fold at 3 dpt, while GABA and M1 treated plants exhibited an induction of 19- and 4-folds at 7 dpt, respectively. A down regulation of the *LOX* gene was observed at 4 dpt on both M1- and M2-treated plants. Regarding *PAL* gene, up regulations of about 3-fold were observed at 7 dpt in plants treated with the three molecules. The expression of this gene was down regulated at 4 dpt in plants treated with M2. Concerning *POX2*, an up-regulation of this gene was recorded at 2, 3 and 4 dpt in plants treated with the three molecules, with induction levels varying between 2- and 18- folds. The highest expression level was obtained for M2 at 2 dpt. At 7 dpt, an induction of *POX2* was observed only in plants treated with M1, while at the same date, a down regulation of this gene was observed in plants treated with GABA. *PR1* gene showed high induction levels when compared to the other targeted genes, especially at 2 dpt, in plants treated with the three molecules, with a most important induction in GABA-treated plants. Up-regulations of *PR1* gene expression in GABA-treated plants ranged from 18- to 347-folds; the highest value (347-fold) was recorded at 2 dpt. In M1-treated plants, *PR1* gene was induced at 2 and 7 dpt (by 179- and 3-folds, respectively), while a down-regulation of this gene was observed at 3 dpt on the same plants. In M2-treated plants, up-regulations of *PR1* at 2 and 3 dpt (by 37- and 4- folds, respectively) were noticed, while down regulations of this gene were observed in the same plants at 3 and 7 dpt (Fig. 5A).

Under infectious conditions, the specific effect of inoculation on wheat plants was assessed in order to highlight the way by which the fungus alone influences the expression of targeted genes. Plants inoculated and treated with Cantor were compared to plants that have received the same treatment but non-inoculated (Fig. 5B). An up-regulation of *LOX* gene expression at 3 and 7 dpt (corresponding to 1 and 5 dpi, respectively) was observed, while a down regulation of this gene was recorded at 4 dpt (2 dpi). *PAL* gene expression was unchanged compared to non-inoculated plants throughout the whole time course. By contrast, both *POX2* and *PR1* gene expressions were up-regulated at all studied timepoints. In order to investigate the priming effect of the molecules, plants inoculated and treated with the three molecules were compared to plants inoculated and treated with water amended with 1% ethanol and 0.15% Cantor (Fig. 5C). *LOX* gene expression was induced at 3 dpt (1 dpi) by 2-, 6- and 10- folds, and at 4 dpt (2 dpi) by 3-, 6- and 2-folds in plants treated with GABA, M1 and M2, respectively. However, down-regulations of this gene were observed at 7dpt (5 dpi) in plants treated with GABA and M2. Regarding *PAL*, at 7 dpt (5 dpi), this gene was induced in plants treated with GABA and down-regulated in plants treated with M1. For *POX2* gene, few modifications of expression were observed compared to the control. Indeed, only an induction of *POX2* gene expression (6-fold) was noticed at 7dpt (5 dpi) in plants treated with M1, while down-regulations of this gene were recorded at 3 dpt (1 dpi) in plants treated with GABA and M2. M1-treated plants showed an up-regulation at 7dpt (5 dpi) and a down-regulation at 4 dpt (2 dpi), while M2-treated plants exhibited down-regulations of *POX2* gene expression at 3 (1 dpi) and 7 dpt (5 dpi).

## Discussion

The present study allowed us to discover new bio-sourced elicitors from sugar beet byproducts that induce resistance in wheat against *Z. tritici*, the major pathogen on this crop. Among the 31 studied molecules, GABA, M1, and 15 molecules functionalized from M1, displayed significant protection levels on wheat without any direct antifungal effect towards the pathogen, indicating that the protection efficacy conferred by these molecules is due to an elicitation effect rather than to a direct activity. Few previous reports have already showed the ability of elicitors to protect wheat against *Z. tritici*. For instance, surfactin, a cyclic lipopeptide from the bacterial strain *Bacillus amyloliquefaciens* S499, was shown to confer protection in wheat towards *Z. tritici* (70 % disease reduction) by activating both salicylic acid- and jasmonic acid-dependent defense pathways^33^. Two commercial elicitors (Vacciplant, marketed by Arysta Lifescience, and Bion, marketed by Syngenta) are registered for the control of *Z. tritici*. These compounds were reported to reduce in wheat *Z. tritici*-induced disease severity by 45% and 69 %, respectively^33,36^. GABA has never been explored before for its protection efficacy on wheat against *Z. tritici*, but its plant protection potential against some phytopathogens has been investigated in previous works. For instance, a treatment of pear fruit with GABA induced resistance against the postharvest blue mold rot caused by *Penicillium expansum*^39^. Few studies were also conducted to examine the effect of GABA on reducing abiotic stress on wheat. For instance, exogenous application of GABA reduced damages caused by chilling stress in wheat seedlings^40^. Likewise, foliar application of a GABA-based formulation on wheat enhanced grain yield and leaf manganese content associated with an improved tolerance to powdery mildew^41^.

The structure-activity relationship study based on chemical modulations made on the molecule M1 revealed that all chemical groups of the molecule, at all positions, are important for its resistance induction activity in wheat. Simultaneous modifications carried out at all positions affected the protection efficacy, either by reducing or by completely inhibiting the activity. Likewise, single chemical functionalization resulted in a loss or stability of the protection level, except the modification performed to obtain the molecule M2 (C8 chain extension) which allowed an enhancement of the protection efficacy from 55 % (M1) to 63 % (M2) disease severity reduction (Table 1). Interestingly, an extension of the carbonic chain to 14 carbons (M4) did not increase the activity; but rather, led to a decrease in the activity (Table 1), suggesting that a moderate length of the chain (C8 for instance) is more suitable to the protection activity. Surprisingly, Log *P* of the molecules were not correlated to the protection efficacy. For instance, the most hydrophobic molecule M25, with a Log *P* of 9.39, did not confer any protection to wheat towards the pathogen (Table 1). According to Schönherr and Bauer^42^, highly hydrophobic leaf cuticles restrict the penetration of hydrophilic agents inside the leaves. Since wheat leaves are highly hydrophobic^43^, it is more likely that hydrophilic molecules used in our study have a low chance to enter inside leaf tissues compared to the hydrophobic (lipophilic) molecules, even if the penetration into the leaves may be insured through leaf stomata as an alternative. However, the use of the wetting agent Heliosol probably allowed, to all molecules, including the hydrophilic ones such as GABA and M1 (with low Log *P* values of −0.62 and −0.7, respectively), to get into the leaves and to confer significant protection rates (44 % and 55 %, respectively). We can thus suggest that the absence of protective activity by the non-active molecules is not due to hydrophilic properties, but more likely to their structure and to the non-recognition of these molecules by the plant, resulting in non-activation of defense mechanisms. For the lipophilic molecules, their application with the wetting agent has likely improved their penetration, consequently their activity.

Regarding Heliosol, although this wetting agent did not show alone any protection *in planta* in the greenhouse, laboratory assays showed that it displays in vitro direct antifungal effect. Such difference between in vitro and *in planta* assays could be the result of a high concentration of the product being in contact with the fungus in in vitro conditions when compared to *in planta* conditions. The lack of protection *in planta* could also be due to its photodegradation, thermolability or volatility, making the *in planta* concentration in contact with the fungus at the leaf surface (inoculated two days later) much lower than the in vitro concentration. In order to guarantee an absence of direct effect from the wetting agent, Cantor was chosen for further experiments since no direct activity was recorded in vitro and no protective effect was conferred *in planta* by this wetting agent when applied alone. Interestingly, the protection efficacies obtained with GABA, M1 and M2 amended with either Heliosol or Cantor were overall similar (Table 1, Figure 3), confirming the non-contribution of the direct activity of Heliosol to the protection efficacy of the molecules and suggesting that the biological activity of the molecules do not vary depending on the used wetting agents.

In-depth characterization of M1, M2 and GABA allowed a better understanding of the mode of action of these molecules. Histopathological observations confirmed the absence of direct effect of these molecules as well as Cantor on both spore germination and fungal hyphal growth on leaf surfaces (Figure 4), supporting the finding that the activity of these molecules is strictly due to plant defense activation. Gene expression monitoring confirmed this result and revealed that the three molecules trigger defense responses in wheat against *Z. tritici*. Since leaf penetration by the fungal hyphae occurs by two days after inoculation^1^, the wheat defense responses activated here by the molecules during the early stages of infection could be the mechanism slowing the fungal development inside the leaves. Overall, the expression levels induced by the molecules in non-infectious conditions (Fig. 5A) were higher than under infectious conditions (Fig. 5C), suggesting that the resistance induced in wheat by the three molecules relies on an elicitation activity rather than on a priming effect. Such a result indicates that the mode of action of GABA (as well as M1 and M2) is different from that of its isomer BABA known as a priming agent of defense reactions^32^. On the other hand, gene inductions under infectious conditions without treatments (Fig. 5B) was much higher than under infectious conditions in presence of treatments by the molecules (Fig. 5C), reporting that the ability of the fungus alone to activate wheat defenses is stronger than that of the molecules. An induction of wheat-defense related genes by *Z. tritici* in absence of treatments, including *LOX*, *PAL*, *POX2* and *PR1* genes targeted in the present study, has previously been reported, with expression patterns that vary depending on the used host cultivar and the sampled time point during the early stages of fungal infection^43–45^.

Regarding genes induced by the molecules, similarities were found among the three molecules, although few differences in the expression level of some genes at some time points were observed in both non-infectious and infectious conditions (Fig. 5A and C). Overall, expression levels of *LOX* gene were higher than *PAL* gene in plants treated with all molecules. LOX activity acts upstream of the octadecanoid pathway, by catalysing the dioxygenation of polyunsaturated fatty acid, leading to the downstream biosynthesis of jasmonic acid^46^. PAL activity is involved in the upstream of phenylpropanoid pathway, catalysing the non-oxidative deamination of phenylalanine to trans-cinnamate, a precursor required for the downstream biosynthesis of phenolic compound-based phenylpropanoids, such as flavonoids, phytoalexins, lignins, and salicylic acid^47,48^. Both jasmonic and salicylic acids are essential signals involved in plant defense responses. Based on our findings, the mode of action of M1, M2 and GABA molecules seems to rely more on jasmonic acid-rather than on salicylic acid-related pathway. On the other hand, the three molecules elicit, with a strong intensity, the expression of *PR1* gene. PR1 is a PR protein with a non-elucidated function, while most of other PR-proteins are known to be antimicrobials having the role of arresting pathogen invasion^49^. Up-regulations of *PR1* gene have already been reported in wheat plants infected with *Z. tritici*^43,45^. Concerning *POX2*, marked expression amounts of this gene were also observed in plants treated with the three molecules. *POX* activity is involved in ROS metabolism and uses hydrogen peroxide (H_2_O_2_) as a substrate during defense responses^50^. The accumulation of H_2_O_2_ has been shown to inhibit colonization by *Z. tritici* in resistant cultivars^51^. Our result showing an induction of *POX2* gene suggests an accumulation of H_2_O_2_ in wheat plants treated with the three molecules; it is likely that the plants then induced POX activity to reduce the rates of H_2_O_2_, since this compound of oxidative stress could display a toxicity to the plant when produced at high concentrations. *POX* gene expression has been associated to innate resistance in wheat against *Z. tritici*. Indeed, Shetty et al.^52^ reported that resistant wheat cultivars accumulate *POX* transcripts and had an important POX activity compared to susceptible cultivars when inoculated with *Z. tritici*.

## Conclusion

The present study reports new resistance inducers bio-sourced from sugar beet byproducts, as well as GABA, that are able to induce resistance in wheat against *Z. tritici*. These molecules provide protection rates of up to 63 % disease severity reduction, without any direct antifungal effect towards the pathogen, indicating that the activity of the molecules results exclusively from plant defense stimulation. Structure-activity relationship study revealed the involvement of all chemical groups (especially the carbonic chain), at all positions, in the activity of the molecule M1 used for functionalization. Gene expression assay confirmed the ability of M1, M2 and GABA to trigger defense reactions in wheat, thus confirming the eliciting properties of these molecules. However, further experiments are required to decipher the defense pathways activated by these molecules in wheat against *Z. tritici*. Moreover, although PGA that served as a parent molecule to produce the elicitors highlighted in the current study is a non-toxic compound, a verification of the absence of toxicity as well as eco-toxicity of the functionalized molecules is required for any future application in plant protection.

## Methods

### General chemistry experimental procedures

Starting materials used for the synthesis of novel molecules, including L-pyroglutamic acid (PGA), 2-oxo-1,3-thiazolidin-4-carboxylic acid (M28), and γ-aminobutyric acid (GABA), were commercially available (TCI Europe and Sigma-Aldrich France) and were used without further purification. Melting points were measured on a MPA 100 OptiMelt apparatus and are uncorrected. NMR spectra were acquired at 400 MHz for ^1^H NMR and 100 MHz for ^13^C NMR on a Varian MR 400 spectrometer. Chemical shifts (δ) are given in ppm relative to CDCl_3_ (7.26 ppm; 77.1 ppm). Splitting patterns are designated as: s, singlet; d, doublet; dd, doublet of doublets; t, triplet; quint, quintuplet; m, multiplet and sym m, symmetric multiplet.

Coupling constants *J* are reported in hertz (Hz). Thin layer chromatography was performed on Macherey Nagel silica gel plates with a fluorescent indicator and were visualized with UV lamp at 254 nm and 366 nm. Column chromatography was performed using a CombiFlash Rf Companion (Teledyne-Isco System) and Macherey-Nagel prepacked columns. IR spectra were recorded on a Varian 640-IR FT-IR Spectrometer. Elemental analyses (C, H, N) of new compounds were determined by “Welience”, Pôle Chimie Moléculaire, Faculté de Sciences Mirande, Université de Bourgogne, Dijon, France. The synthetic methodology employed to obtain the novel derivative molecules is fully described in Supporting Information section.

### Plant growth, treatment and inoculation

Grains of the susceptible bread wheat (*Triticum aestivum* L.) cultivar Alixan (Limagrain, France) were pre-germinated on moist filter paper in Petri dishes (12 × 12 cm) according to Ors et al.^45^, and then transferred into three-liter pots filled with universal loam (Gamm Vert, France). The pots were placed in the greenhouse under semi-controlled conditions at 18 °C (± 2 °C) with a day-night cycle of 16/8 h with a supplementary lighting. In the plant protection efficacy assay performed in the structure-activity relationship analysis, all molecules (M1 to M29), as well as PGA and GABA, were first dissolved in ethanol (1% v/v final concentration) and incubated in an ultrasonic bath to insure solubilization, before being mixed with distilled water containing the wetting agent Heliosol at 0.2% v/v final concentration. In the further experiments related to the cytological and gene expression assays focused on GABA, M1, and M2 molecules, the wetting agent Heliosol was substituted by Cantor at 0.15% v/v final concentration since Heliosol exhibited a direct antifungal activity against *Z. tritici*. When the third leaf is fully expended (fourth leaf is emerging), plants were treated with 5 mM of each molecule using a manual hand sprayer. Two days after treatment, plants were inoculated with *Z. tritici* by spraying a spore suspension at 10^6^ spores.mL^-1^ of the *Z. tritici* pathogenic strain T02596 (isolated in 2014 from northern France), amended with 0.05 % polyoxyethylene-sorbitan monolaurate (Tween 20, Sigma Aldrich, USA). Immediately after inoculation, pots were covered with clear polyethylene bags for three days in order to ensure a high hygrometry required for good fungal germination. The disease severity was assessed at 21 days post-inoculation (dpi) only on the third leaves by scoring the percentage of diseased leaf area (chlorosis and necrosis) bearing pycnidia^1^. Thirty-six plants (three pots of 12 plants each) were used as replicates for each condition, including controls, and all experiments were repeated twice in time.

### In vitro antifungal activity

Direct effect of the molecules was assessed in clear and sterile flat-bottomed polystyrene microplates (Iwaki, Asahi techno glass, Japan) with twelve columns of eight wells, according to Siah et al.^53^. Molecules, first dissolved in 1% ethanol of final volume, were added to the medium at 50 °C following autoclaving. Plate wells were each filled with 150 μL of liquid glucose peptone medium (14.3 g L^−1^ dextrose (VWR), 7.1 g L^−1^ bactopeptone (Difco laboratories) and 1.4 g L^−1^ yeast extract (Merck)) amended with the molecules at 5 mM (final concentration in 200 μL of medium). For each molecule, 16 wells were used, among them, 8 were amended with aliquots of 50 μL suspension containing 2.10^5^ spores mL^−1^ of the *Z. tritici* strain T02596 and 8 were amended with 50 μL of distilled sterile water. In each microplate, non-inoculated medium without molecules, as well as inoculated medium without molecules, were used as experimental controls. Plates were incubated for 6 days at 20°C in the dark while being shaken at 140 rpm, after which fungal growth was measured using a plate reader (MRX, Dynex technologies) at 405 nm. Eight wells were used as replicates for each condition.

Since the color of the wetting agents Heliosol and Cantor is strongly influenced by the optical density, the antifungal activity of these compounds was assessed on potato dextrose agar (PDA) medium, according to Siah et al.^53^. The compounds were added to PDA medium at 50 °C after autoclaving at 5 mM for the molecules and at 0.2% and 0.15% for Heliosol and Cantor, respectively. Five aliquots of 5 μL of 5.10^5^ spores mL^−1^ fungal suspension were spotted on each PDA plate. After incubation for 10 days at 20 °C in the dark, the colony perpendicular diameters were measured for each spot and compared to spots developed on the control medium. Three Petri dishes with five spots (colonies) in each were used as replicates for each condition.

### Epiphytic fungal growth

Fungal growth on leaf surfaces of inoculated plants treated with water alone, water amended with 1% ethanol and 0.15 % Cantor or with M1, M2 and GABA molecules, was monitored at 1 and 5 dpi using Fluorescence Brightener 28 (Calcofluor, Sigma Aldrich) staining, according to Siah et al.^1^. Third-leaf segments (4 cm) were collected from plants of each condition before being stained with a solution of 0.1 % Calcofluor 0.1 M Tris-HCl buffer at pH 8.5 and washed with sterile distilled water. After drying in darkness at laboratory temperature, leaf segments were placed on a glass slide, covered with a cover slip and observed under a microscope with UV illumination (Nikon, Eclipse 80i). The effect of different treatments on hyphal growth was assessed at 1 dpi by determining the percentage of germinated spores, and at 5 dpi by recording four fungal cytological event classes (class 1, non-germinated spore; class 2, geminated spore with minor germ tube; class 3, geminated spore with developed germ tube; class 4, geminated spore with a strongly developed germ tube). Microscopic observations at both 1 and 5 dpi were performed on 100 randomly-chosen fungal spores on each leaf segment. Three third-leaf segments were as replicates for each condition.

### Plant gene expression

The expression of four genes involved in wheat defense mechanisms, including genes encoding for LOX (lipoxygenase), PAL (phenylalanine ammonia-lyase), POX (peroxidase) and PR1 (pathogenesis-related protein 1), was monitored at 2, 3, 4 and 7 days post treatment (dpt) in non-inoculated conditions and at 3, 4 and 7 dpt (corresponding to 1, 2 and 5 dpi) in inoculated conditions, according to Ors et al.^45^. ACT (actin) and TUB (*β*-tubulin) encoding genes were used as housekeeping genes. Leaf samples from plants treated with water alone, water amended with 1% ethanol and 0.15 % Cantor or with GABA, M1 and M2 molecules, all inoculated or not with *Z. tritici*, were collected and submitted for RNA extraction using the RNeasy Plant Mini Kit (Quiagen, The Netherlands). The extraction of total RNA was carried out from 100 mg of plant tissue, and genomic DNA potentially contaminating the samples was removed by treatment with DNase using RNase-Free DNase Set (Quiagen, The Netherlands). The obtained RNA was suspended in 60 µL of RNase-free water and quantified by measuring the absorbance at 260 nm (Eppendorf AG, BioPhotometer). Reverse transcription of the obtained RNA was performed with the High Capacity cDNA Reverse Transcription Kit (Applied Biosystems, USA) according to the manufacturer’s protocol. PCR reactions were undertaken on the subsequent obtained cDNA to amplify the six genes described above. Cycling conditions consisted of a denaturation cycle (95 °C for 3:10 min) and an annealing/extension cycle repeated 39 times (30 s at 60 °C). Primer efficiencies were calculated for different genes by performing real-time PCR with dilutions series with factor 3 made on cDNA samples from several conditions (Supplementary Table S1). PCR reactions were performed in duplicates for each sampled third leaf. In non-inoculated conditions, plants non-inoculated and treated with both Cantor and ethanol were used as control for plants non-inoculated and treated with the molecules. In inoculated conditions, plants non-inoculated and treated with both Cantor and ethanol were also used as control for plants inoculated and treated with water alone. Plants inoculated and treated with both Cantor and ethanol were used as control for plants inoculated and treated with the molecules. Two independent experiments, with three biological replicates each, were performed for qPCR assay. Each biological replicate corresponds to one wheat third leaf sampled from an independent pot. In addition, qPCR reactions were performed in duplicates (two technical replicates) for each sample.

### Data analyses

Comparisons between percentage of disease severity, *in planta* spore germination at 1 dpi, and *in planta* hyphal growth at 5 dpi were carried out with the Tukey test at a significance level of *P* = 0.05 using the XLSTAT software (Addinsoft, France). The correlation between lipophilic property (Log *P*) and protection efficacy of the molecules was performed with the Pearson test using the XLSTAT software. For gene expression analysis, the genes were considered significantly down- or up-regulated compared to controls when changes in their expression were < 0.5 x or > 2 x, respectively. Values of gene expression were converted to Log 2 and represented in the form of a heatmap using the GraphPad Prism software version 9 (GraphPad Software Inc., San Diego, United States). Shades of purple to blue represent gene down regulation, while the shades of green to yellow represent gene up-regulation.

## Supplementary Information

Supplementary Information.

## Data Availability

All data generated and analyzed during the present study are included in this published article and its supplementary information files. The corresponding original datasets are available from the corresponding author on reasonable request.
