# Supplementary material for: New plant immunity elicitors from a sugar beet byproduct protect wheat against Zymoseptoria tritici
Source: Sci Rep. 2023 Jan 3;13:90. doi: 10.1038/s41598-022-26800-z (PMC9810720; doi:10.1038/s41598-022-26800-z)
Supplement: Supplementary file 1 — Supplementary Information. [file 41598_2022_26800_MOESM1_ESM.pdf]

Supporting Information

Supplementary Figure S1. Experimental procedure related to the synthesis of the compounds assessed in the present study.

General Remarks. Starting materials were commercially available and were used without further purification. Melting points were measured on a MPA 100 OptiMelt apparatus and are uncorrected. NMR spectra were acquired at 400 MHz for ^{1}H NMR and 100 MHz for ^{13}C NMR on a Varian MR 400 spectrometer. Chemical shifts (δ) are given in ppm relative to $CDCl_3$ (7.26 ppm; 77.1 ppm). Splitting patterns are designed as: s, singlet; d, doublet; dd, doublet of doublets; t, triplet; quint, quintuplet; m, multiplet and sym m, symmetric multiplet. Coupling constants *J* are reported in hertz (Hz). Thin layer chromatography was realized on Macherey Nagel silica gel plates with a fluorescent indicator and were visualized with UV-lamp at 254 nm and 366 nm. Column chromatographies were performed using a Combi*Flash* Rf Companion (Teledyne-Isco System) and Redi*Sep* prepacked columns. IR spectra were recorded on a Varian 640-IR FT-IR Spectrometer. Elemental analyses (C, H, N) of new compounds were determined by "Welience", Faculté de Sciences Mirande, Université de Bourgogne, Dijon, France.

γ-Aminobutyric acid (GABA), L-pyroglutamic acid and 2-oxo-1,3-thiazolidin-4-carboxylic acid **M28** were commercially available.

General procedure for the synthesis of acylureas M1, M2, M4, M5, M7 and M8 (Reaction 1). Isocyanate (R-NCO) (1-1.2 equiv.) dissolved in toluene was added dropwise with a syringe to a stirred solution of methyl pyroglutamate (**M9**) (1 equiv.) in refluxed toluene (20 mL) (Reaction 1). The mixture was stirred under nitrogen atmosphere at reflux for 24-48 h. After cooling to room temperature, methanol was added to the medium. The mixture was concentrated and purified by flash chromatography (elution with *n*-heptane/ethyl acetate: 80/20 → *n*-heptane/ethyl acetate: 0/100) to afford the pure products.

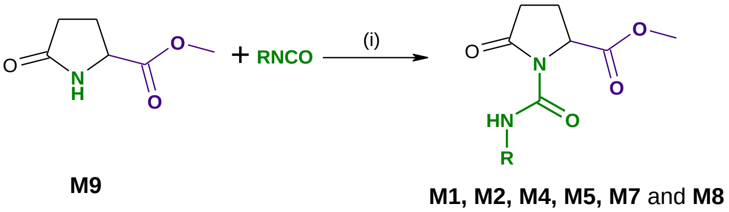

Reaction 1. *Reagents and conditions: (i) 1-1.2 eq. R-NCO, toluene, reflux, 24-48 h.*

Methyl 1-[(butylamino)carbonyl]-5-oxoprolinate (**M1**)

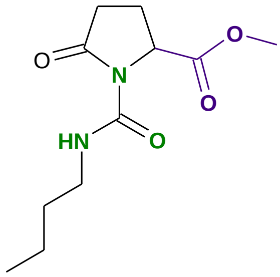

Acylurea **M1** was obtained with the same physico-chemical properties as previously described in the literature by our group.[i]

Methyl 1-[(dodecylamino)carbonyl]-5-oxoprolinate (**M2**)

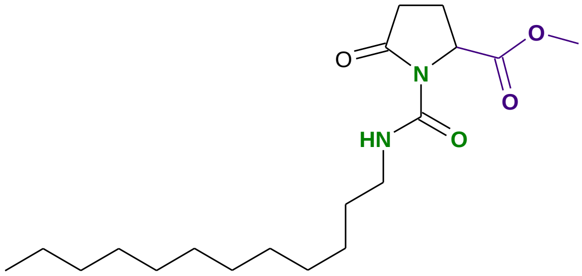

The general procedure was followed using methyl pyroglutamate (**M9**) (2.82 g, 19.7 mmol, 1 equiv.) in toluene (20mL) and *n*-dodecyl isocyanate (5.00 g, 23.6 mmol, 1.2 equiv.) and the mixture heated at reflux under nitrogen atmosphere for 24 hours. After cooling to room temperature, methanol (20 mL) was added. The residue was concentrated and separated by chromatography on flash chromatography (elution with *n*-heptane/ethyl acetate: 80/20 → *n*-heptane/ethyl acetate: 0/100) to generate pure product **M2** as yellow oil in 55% yield, R_f (EtOAc/*n*-heptane 50/50) = 0.67;

IR ν cm^{-1}: 3316, 2922, 2853, 1720, 1539, 1438, 1378, 1206, 1043, 596.

^{1}H NMR (400 MHz, $CDCl_3$) δ ppm: 0.88 (t, *J* = 6.8 Hz, 3H, $CH_2(CH_2)_9CH_2CH_3$), 1.26 (br s, 18H, $CH_2(CH_2)_9CH_2CH_3$), 1.54 (quint, *J* = 13.9, 6.9, 2.0 Hz, 2H, $CH_2(CH_2)_9CH_2CH_3$), 2.01-2.10 (m, 1H, CH_2CH_2CH), 2.27-2.40 (m, 1H, CH_2CH_2CH), 2.52-2.62 (m, 1H, CH_2CH_2CH), 2.69-2.80 (m, 1H, CH_2CH_2CH), 3.20-3.36 (m, 2H, $CH_2(CH_2)_9CH_2CH_3$), 3.79 (s, 3H, OCH_3), 4.80 (dd, *J* = 9.8, 2.7 Hz, 1H, CH_2CH_2CH), 8.27 (br s, 1H, NH).

^{13}C NMR (100 MHz, $CDCl_3$) δ ppm: 14.1 (CH_3), 21.3 (CH_2), 22.7 (CH_2), 26.9 (CH_2), 29.3 (CH_2), 29.4 (CH_2), 29.5 (CH_2), 29.6 ($3CH_2$), 29.7 ($2CH_2$), 31.9 (CH_2), 32.0 (CH_2), 40.0 (CH_2), 52.7 (CH_3), 58.0 (CH), 152.2 (C), 172.0 (C), 176.3 (C).

Anal. calcd for **$C_{19}H_{34}N_2O_4$** (354.49 g/mol): C, 64.38; H, 9.67; N, 7.90%. Found: C, 64.76; H, 9.78; N, 7.87%.

LogP: 3.55 ± 0.62.

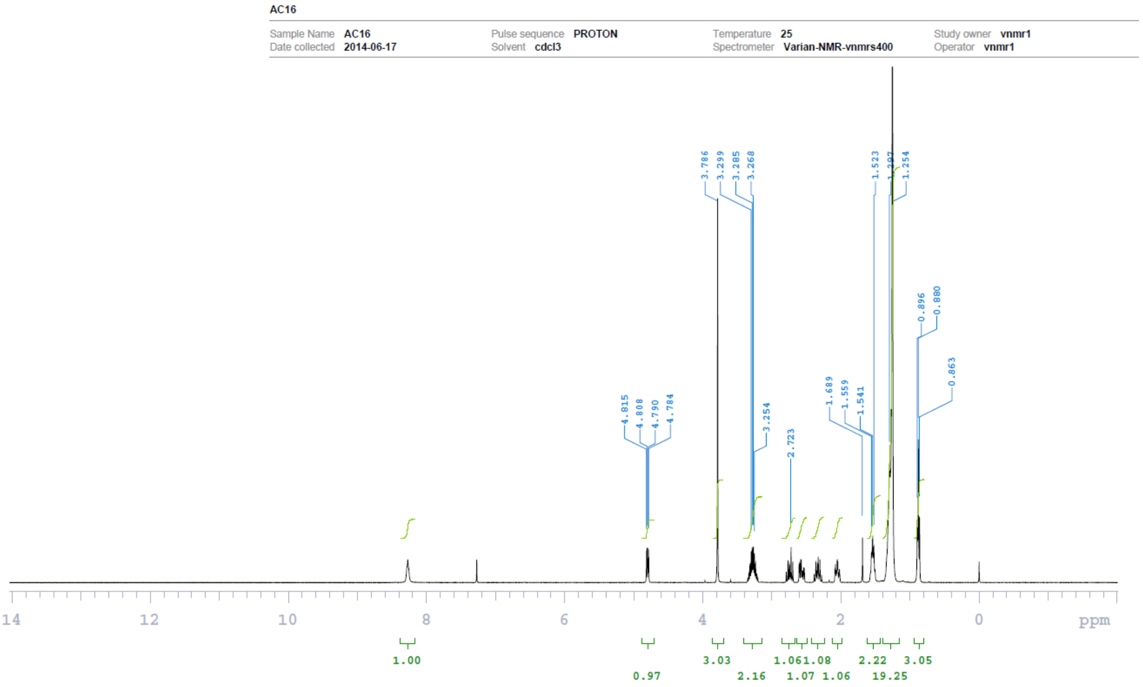

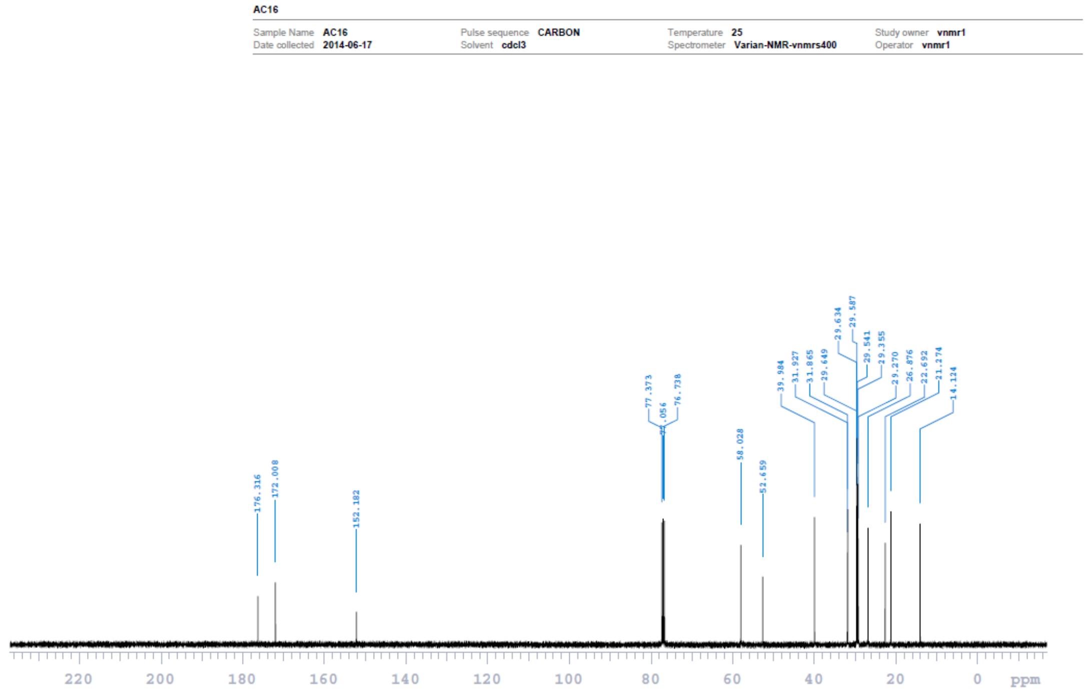

Methyl 1-[(octadecylamino)carbonyl]-5-oxoprolinate (**M4**)

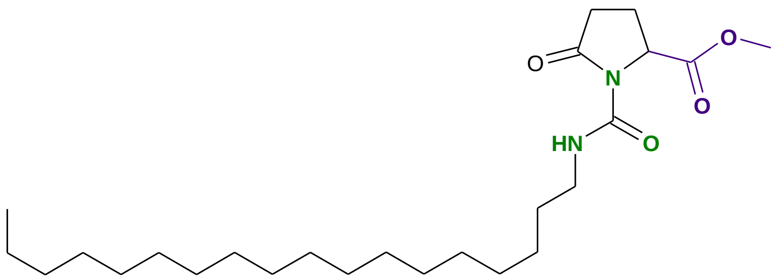

The general procedure was followed using methyl pyroglutamate (**M9**) (0.97 g, 6.8 mmol, 1 equiv.) in toluene (20mL) and *n*-octadecyl isocyanate (2.0 g, 6.8 mmol, 1 equiv.) and the mixture heated at reflux under nitrogen atmosphere for 48 hours. After cooling to room temperature, methanol (20 mL) was added. The residue was concentrated and separated by flash chromatography on silica prepacked column (elution with *n*-heptane/ethyl acetate: 80/20 → *n*-heptane/ethyl acetate: 0/100) to generate pure product **M4** as a white solid in 53% yield, R_f (EtOAc/*n*-heptane 50/50) = 0.65;

IR ν cm^{-1}: 3336, 2918, 1750, 1716, 1532, 1462, 1242, 1202, 720, 584.

^{1}H NMR (400 MHz, $CDCl_3$) δ ppm: 0.88 (t, *J* = 6.9 Hz, 3H, $CH_2(CH_2)_{15}CH_2CH_3$), 1.25 (br s, 30H, $CH_2(CH_2)_{15}CH_2CH_3$), 1.50-1.60 (m, 2H, $CH_2(CH_2)_{15}CH_2CH_3$), 2.01-2.09 (m, 1H, CH_2CH_2CH), 2.27-2.40 (m, 1H, CH_2CH_2CH), 2.52-2.61 (m, 1H, CH_2CH_2CH), 2.69-2.80 (m, 1H, CH_2CH_2CH), 3.20-3.36 (m, 2H, $CH_2(CH_2)_{15}CH_2CH_3$), 3.79 (s, 3H, OCH_3), 4.80 (dd, *J* = 9.4, 2.7 Hz, 1H, CH_2CH_2CH), 8.27 (br s, 1H, N*H*).

^{13}C NMR (100 MHz, $CDCl_3$) δ ppm: 14.1 (CH_3), 21.2 (CH_2), 22.7 (CH_2), 26.7 (CH_2), 29.2 (CH_2), 29.3 (CH_2), 29.5 (CH_2), 29.5 (CH_2), 29.6 (CH_2), 29.6 (CH_2), 29.7 (7CH_2), 31.8 (CH_2), 31.9 (CH_2), 40.1(CH_2), 52.6 (CH_3), 58.0 (CH), 152.1 (C), 172.0 (C), 176.3 (C).

Anal. calcd for **$C_{25}H_{46}N_2O_4$** (438.66 g/mol): C, 68.45; H, 10.57; N, 6.39%. Found: C, 68.27; H, 10.30; N, 6.26%.

LogP: 6.73 ± 0.62.

AC83

Sample Name AC83	Pulse sequence PROTON	Temperature 25	Study owner vnmr1
Date collected 2015-04-03	Solvent cdcl3	Spectrometer w0275-vnmrs400	Operator vnmr1

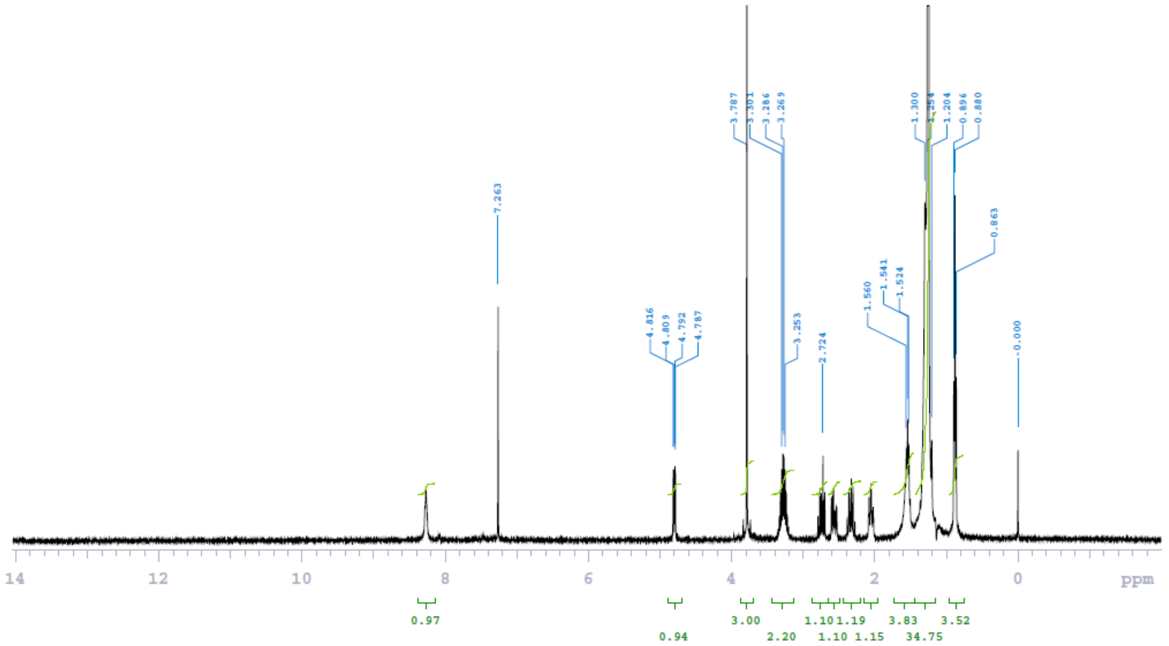

AC83

Sample Name AC83	Pulse sequence CARBON	Temperature 25	Study owner vnmr1
Date collected 2015-04-03	Solvent cdcl3	Spectrometer w0275-vnmrs400	Operator vnmr1

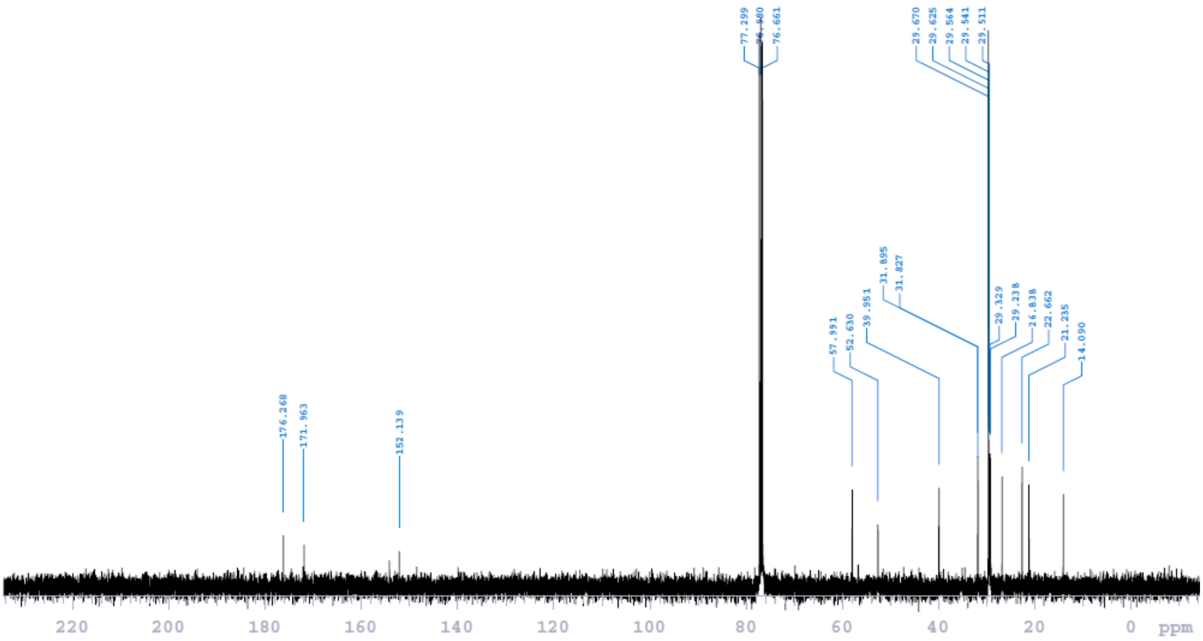

Methyl 1-[(1-adamantylamino)carbonyl]-5-oxoprolinate (**M5**)

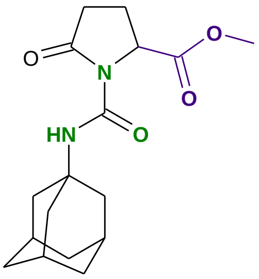

The general procedure was followed using methyl pyroglutamate (**M9**) (4.05 g, 28.0 mmol, 1 equiv.) in toluene (20mL) and 1-adamantyl isocyanate (5.00 g, 28.0 mmol, 1.2 equiv.) and the mixture heated at reflux under nitrogen atmosphere for 24 hours. After cooling to room temperature, methanol (20 mL) was added. The residue was concentrated and separated by flash chromatography on silica column (elution with *n*-heptane/ethyl acetate: 80/20 → *n*-heptane/ethyl acetate: 0/100) to generate pure product **M5** as a white solid in 53% yield, mp 147-148 °C, R_f (EtOAc/*n*-heptane 50/50) = 0.67.

IR ν cm^{-1}: 3287, 2900, 1747, 1717, 1546, 1379, 1277, 1200, 768, 609.

^{1}H NMR (400 MHz, $CDCl_3$) δ ppm: 1.65-1.70 (m, 6H, 3CH_2), 1.98-2.03 (m, 7H, 3CH_2 +CH_2CH_2CH), 2.05-2.10 (m, 3H, 3C*H*), 2.22-2.35 (m, 1H, CH_2CH_2CH), 2.49-2.60 (m, 1H, CH_2CH_2CH), 2.66-2.78 (m, 1H, C$H_2$$CH_2$CH), 3.78 (s, 3H, OC$H_3$), 4.76 (dd, *J* = 9.4, 2.8 Hz, $CH_2$$CH_2$C*H*), 8.20 (br s, 1H, N*H*).

^{13}C NMR (100 MHz, $CDCl_3$) δ ppm: 21.0 (CH_2), 29.6 (3CH), 32.0 (CH_2), 36.3 (3CH_2), 41.6 (3CH_2), 51.6 (C), 52.6 (CH_3), 57.9 (CH), 150.3 (C), 172.0 (C), 176.3 (C).

Anal. calcd for $\mathbf{C_{17}H_{24}N_2O_4}$ (320.39 g/mol): C, 63.73; H, 7.55; N, 8.74%. Found: C, 63.62; H, 7.91; N, 8.76%.

LogP: -0.58 ± 0.63.

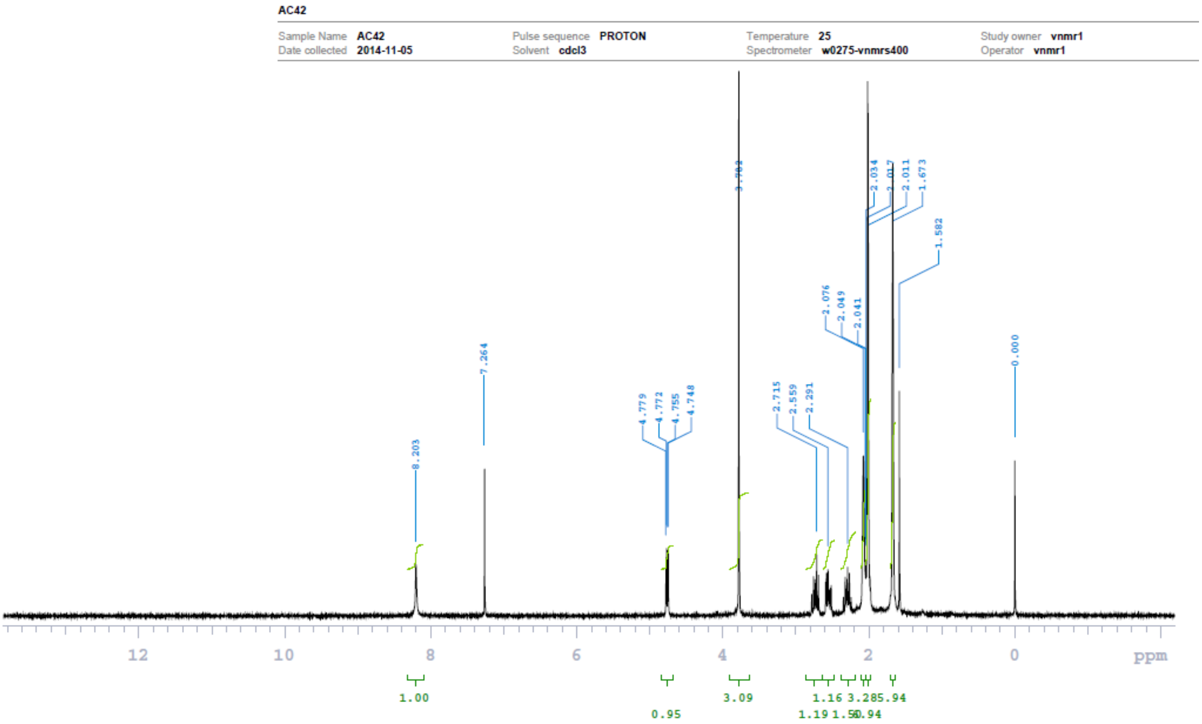
AC42
Sample Name AC42
Date collected 2014-11-05
Pulse sequence PROTON
Solvent cdcl3
Temperature 25
Spectrometer w0275-vnmrs400
Study owner vnmr1
Operator vnmr1
ppm

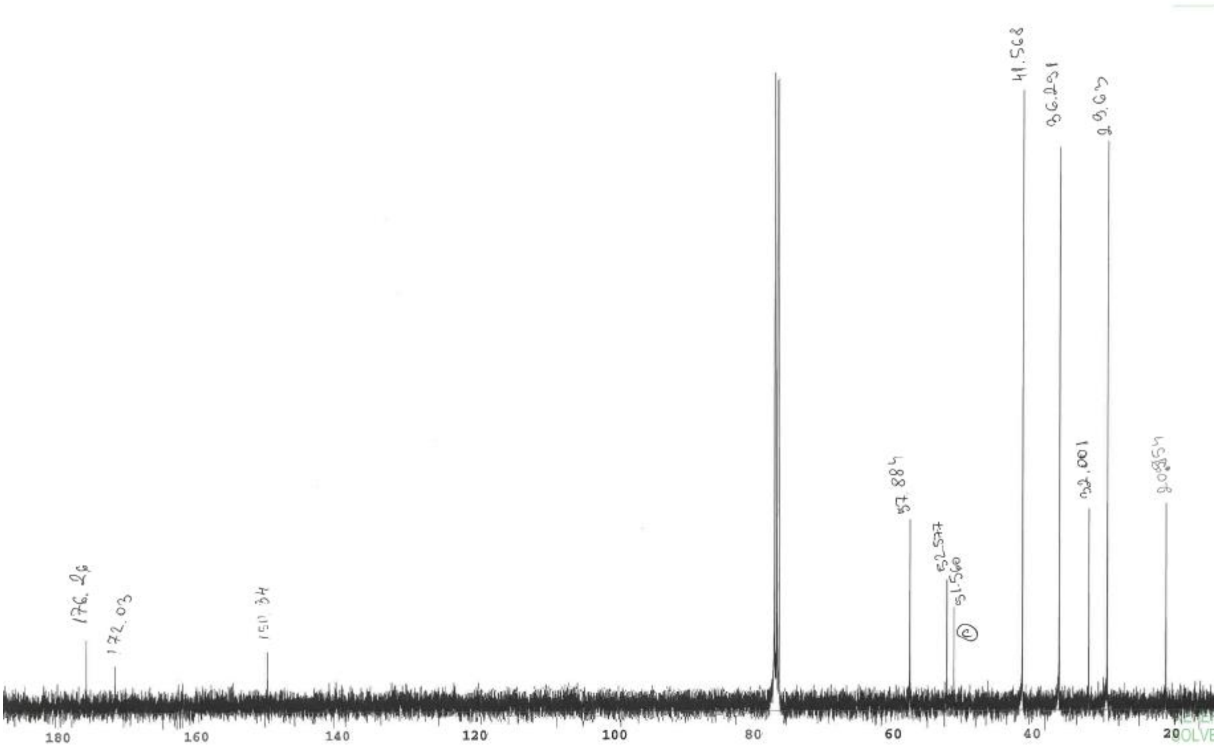

Methyl 5-oxo-1-[(propylamino)carbonyl]prolinate (**M7**)

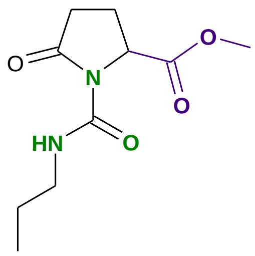

The general procedure was followed using methyl pyroglutamate (**M9**) (5.09 g, 35.5 mmol, 1 equiv.) in toluene (20mL) and *n*-propyl isocyanate (3.63 g, 42.7 mmol, 1.2 equiv.) and the mixture was heated at reflux under nitrogen atmosphere for 26 hours. After cooling to room temperature, methanol (20 mL) was added. The residue was concentrated and separated by flash chromatography on silica column (elution with *n*-heptane/ethyl acetate: 80/20 → *n*-heptane/ethyl acetate: 0/100) to generate pure product **M7** as a yellow solid in 56% yield, mp 50-51 °C, R_f (EtOAc/*n*-heptane 50/50) = 0.54.

IR ν cm^{-1}: 3299, 2960, 2875, 1747, 1714, 1681, 1537, 1461, 1258, 1205, 993, 544.

^{1}H NMR (400 MHz, $CDCl_3$) δ ppm: 0.94 (t, J = 7.5 Hz, 3H, $NHCH_2CH_2CH_3$), 1.58 (sext, J = 14.5, 7.4 Hz, 2H, $NHCH_2CH_2CH_3$), 2.02-2.10 (m, 1H, CH_2CH_2CH), 2.29-2.40 (m, 1H, CH_2CH_2CH), 2.53-2.62 (m, 1H, CH_2CH_2CH), 2.70-2.80 (m, 1H, CH_2CH_2CH), 3.19-3.33 (m, 2H, $NHCH_2CH_2CH_3$), 3.79 (s, 3H, OCH_3), 4.80 (dd, J = 9.7, 2.8 Hz, 1H, CH_2CH_2CH), 8.30 (br s, 1H, NH).

^{13}C NMR (100 MHz, $CDCl_3$) δ ppm: 11.3 (CH_3), 21.3 (CH_2), 22.9 (CH_2), 31.9 (CH_2), 41.6 (CH_2), 52.7 (CH_3), 58.0 (CH), 152.2 (C), 172.0 (C), 176.3 (C).

Anal. calcd for $\mathbf{C_{10}H_{16}N_2O_4}$ (228.25 g/mol): C, 52.62; H, 7.07; N, 12.27%. Found: C, 52.49; H, 7.19; N, 12.38%.

LogP: -1.24 ± 0.62.

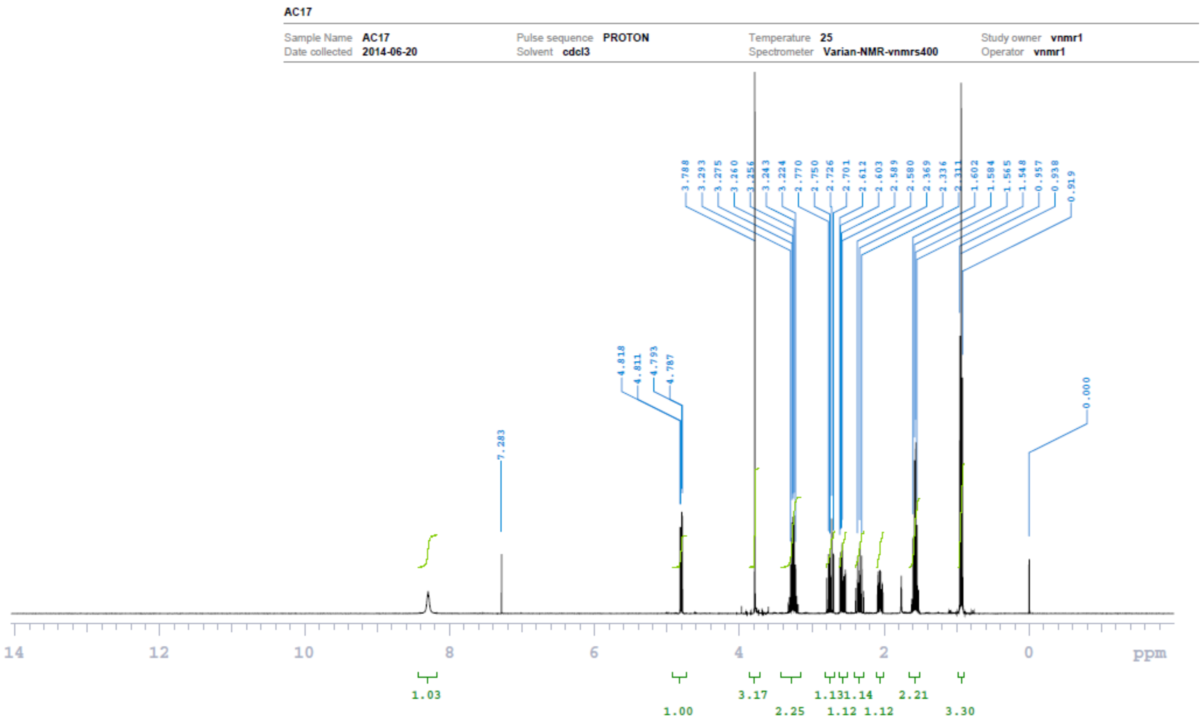
AC17
Sample Name AC17
Date collected 2014-06-20
Pulse sequence PROTON
Solvent cdcl3
Temperature 25
Spectrometer Varian-NMR-vnmrs400
Study owner vnmr1
Operator vnmr1
ppm
1.03
1.00
3.17
2.25
1.13
1.14
1.12
1.12
2.21
3.30

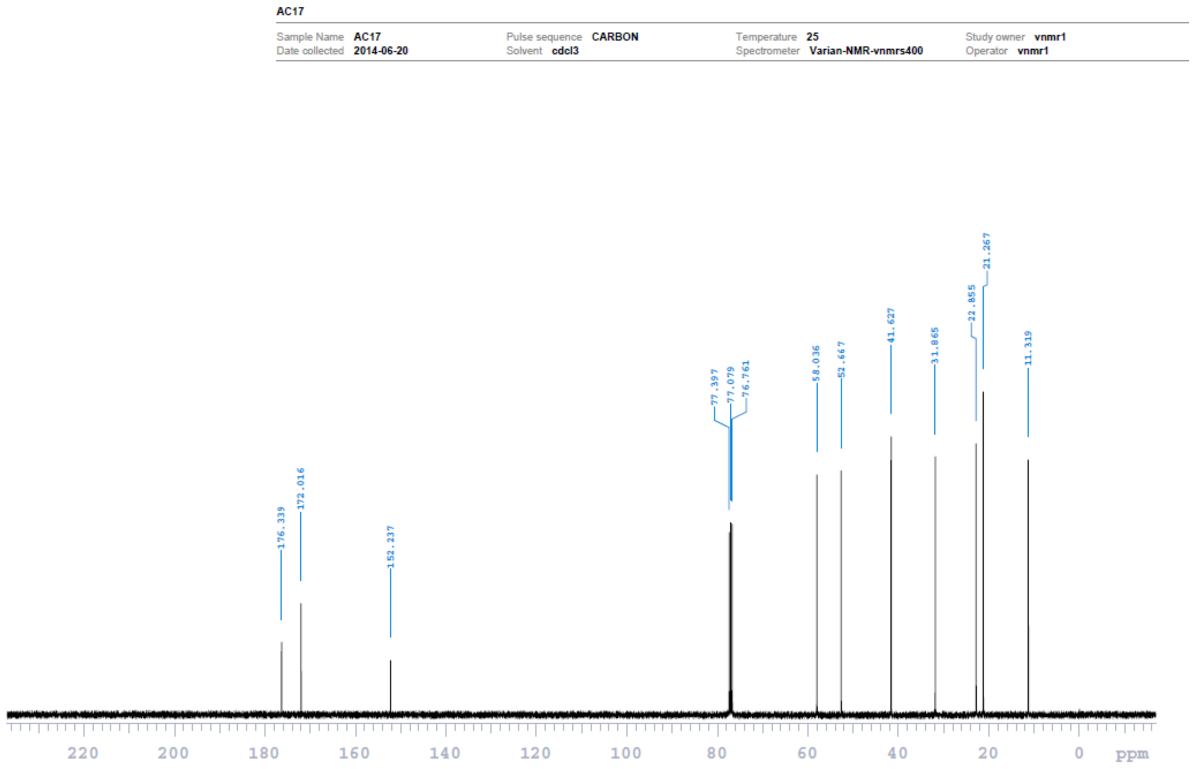
AC17
Sample Name AC17
Date collected 2014-06-20
Pulse sequence CARBON
Solvent cdcl3
Temperature 25
Spectrometer Varian-NMR-vnmrs400
Study owner vnmr1
Operator vnmr1
176.339
172.016
152.237
77.397
77.079
76.761
58.036
52.667
41.627
31.865
22.855
21.267
11.319
ppm

Methyl 5-oxo-1-[(tetradecylamino)carbonyl] prolinate (**M8**)

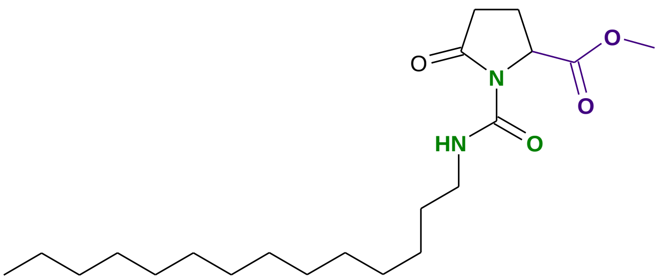

The general procedure was followed using methyl pyroglutamate (**M9**) (1.79 g, 12.5 mmol, 1 equiv.) in toluene (20mL) and *n*-tetradecyl isocyanate (3.0 g, 12.5 mmol, 1 equiv.) and the mixture was heated at reflux under nitrogen atmosphere for 24 hours. After cooling to room temperature, methanol (20 mL) was added. The residue was concentrated and separated by flash chromatography on silica column (elution with *n*-heptane/ethyl acetate: 80/20 → *n*-heptane/ ethyl acetate: 0/100) to generate pure product **M8** as a white solid in 26% yield, mp 44-45°C, R_f (EtOAc/*n*-heptane 50/50) = 0.68.

IR ν cm^{-1}: 3338, 2917, 2848, 1743, 1721, 1535, 1462, 1341, 1206, 989, 721, 596.

^{1}H NMR (400 MHz, $CDCl_3$) δ ppm: 0.88 (t, *J* = 7.1 Hz, 3H, $CH_2(CH_2)_{11}CH_2CH_3$), 1.25 (br s, 22H, $CH_2(CH_2)_{11}CH_2CH_3$), 1.49-1.57 (m, 2H, $CH_2(CH_2)_{11}CH_2CH_3$), 2.01-2.10 (m, 1H, CH_2CH_2CH), 2.27-2.39 (m, 1H, CH_2CH_2CH), 2.52-2.61 (m, 1H, CH_2CH_2CH), 2.69-2.80 (m, 1H, CH_2CH_2CH), 3.20-3.36 (m, 2H, $CH_2(CH_2)_{11}CH_2CH_3$), 3.79 (s, 3H, OCH_3), 4.80 (dd, *J* = 9.4, 2.7 Hz, 1H, CH_2CH_2CH), 8.27 (br s, 1H, N*H*).

^{13}C NMR (100 MHz, $CDCl_3$) δ ppm: 14.1 (CH_3), 21.2 (CH_2), 22.7 (CH_2), 26.8 (CH_2), 29.2 (CH_2), 29.3 (CH_2), 29.5 (CH_2), 29.6 ($5CH_2$), 29.7 (CH_2), 31.8 (CH_2), 31.9 (CH_2), 39.9 (CH_2), 52.6 (CH_3), 58.0 (CH), 152.1 (C), 172.0 (C), 176.3 (C).

Anal. calcd for **$C_{21}H_{38}N_2O_4$** (382.55 g/mol): C, 65.94; H, 10.01; N, 7.32%. Found: C, 66.26; H, 10.30; N, 7.48%.

LogP: 4.61 ± 0.62.

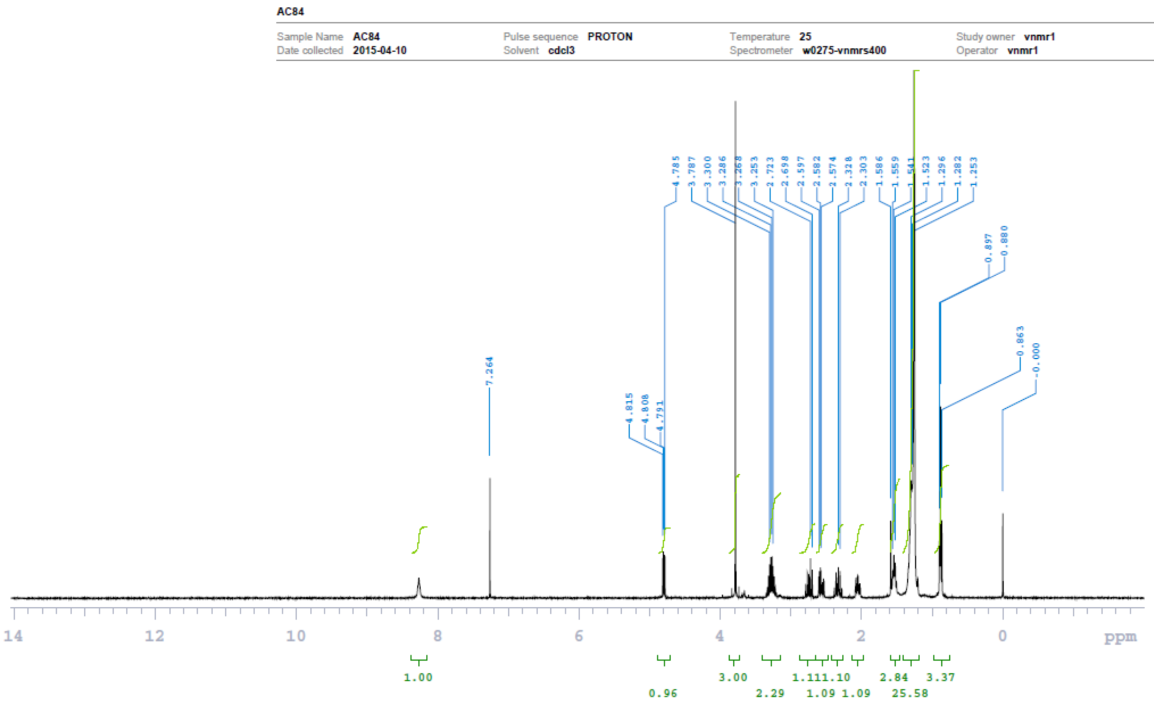
AC84
Sample Name AC84
Date collected 2015-04-10
Pulse sequence PROTON
Solvent cdcl3
Temperature 25
Spectrometer w0275-vnmrs400
Study owner vnmr1
Operator vnmr1
4.785
3.787
3.300
3.286
3.268
3.253
2.723
2.698
2.597
2.582
2.574
2.328
2.303
1.586
1.559
1.541
1.523
1.296
1.282
1.253
0.897
0.880
0.863
0.000
7.264
4.815
4.808
4.791
14
12
10
8
6
4
2
0
ppm
1.00
0.96
3.00
2.29
1.11
1.10
1.09
1.09
2.84
25.58
3.37

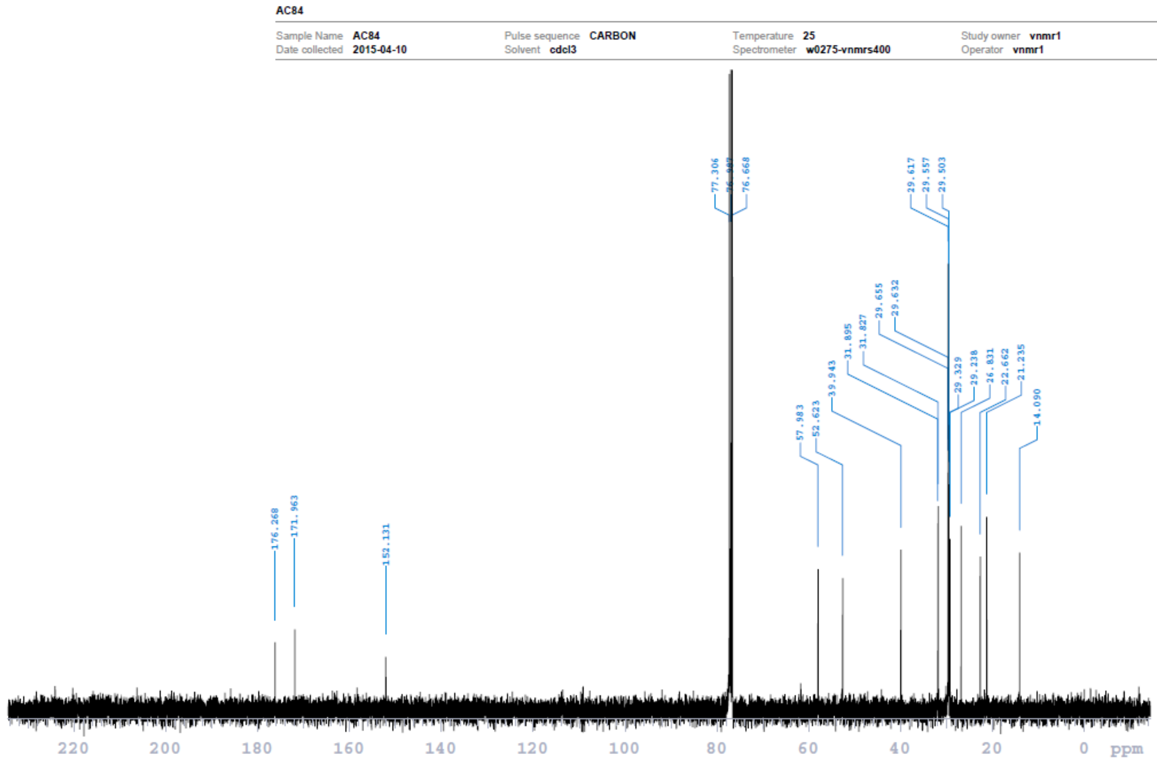
AC84
Sample Name AC84
Date collected 2015-04-10
Pulse sequence CARBON
Solvent cdcl3
Temperature 25
Spectrometer w0275-vnmrs400
Study owner vnmr1
Operator vnmr1
77.306
76.668
29.617
29.557
29.503
29.655
29.632
31.895
31.827
39.943
29.329
29.238
26.831
22.662
21.235
14.090
57.983
52.623
176.268
171.963
152.131
220
200
180
160
140
120
100
80
60
40
20
0
ppm

1-Hexanoyl-5-oxo-pyrrolidine-2-carboxylic acid methyl ester (**M3**)

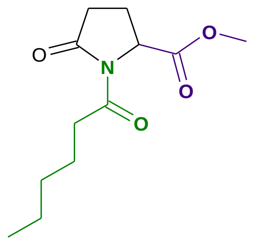

A mixture of methyl pyroglutamate **M9** (5.0 g, 34.9 mmol), hexamethyldisilazane (HMDS) (5.63 g, 34.9 mol), saccharine (0.08 g, 0.70 mmol) and hexanoyl chloride (1.2 eq.), in dichloromethane (30mL) was stirred under nitrogen atmosphere at reflux for 24 h. The residue was partitioned between water and ethyl acetate. The organic layer was dried over anhydrous $MgSO_4$ and then filtered and concentrated. The residue was separated by flash chromatography on silica pre-packed column, eluting EtOAc/*n*-heptane 5:5 to generate pure product **M3** as a white solid in 40% yield, mp 78-80 °C, R_f (EtOAc/*n*-heptane 50/50) = 0.65.

IR ν cm^{-1}: 2951, 1736, 1684, 1645, 1541, 1494, 1399, 1242, 1209, 1155, 1018, 698, 421.

^{1}H NMR (400 MHz, $CDCl_3$) δ ppm: 0.89 (t, J = 7.0 Hz, 3H, CH_3), 1.30-1.36 (m, 4H, 2CH_2), 1.59-1.68 (m, 2H, CH_2), 2.03-2.11 (m, 1H, CH_2CH_2CH), 2.27-2.39 (m, 1H, CH_2CH_2CH), 2.52-2.60 (m, 1H, C$H_2$$CH_2$CH), 2.67-2.77 (m, 1H, C$H_2$$CH_2$CH), 2.82-3.01 (m, 2H, C$H_2$), 3.77 (s, 3H, OC$H_3$), 4.76 (dd, *J* = 9.5, 2.8 Hz, 1H, CH_2CH_2C*H*).

^{13}C NMR (100 MHz, $CDCl_3$) δ ppm: 13.9 (CH_3), 21.3 (CH_2), 22.4 (CH_2), 23.6 (CH_2), 31.2 (CH_2), 32.0 (CH_2), 36.5 (CH_2), 52.6 (CH_3), 57.8 (CH), 171.6 (C), 174.2 (2C).

Anal. calcd for **$C_{12}H_{19}NO_4$** (241.29 g/mol): C, 59.73; H, 7.94; N, 5.80%. Found: C, 59.60; H, 7.80; N, 6.26%.

LogP: 1.71 ± 0.26.

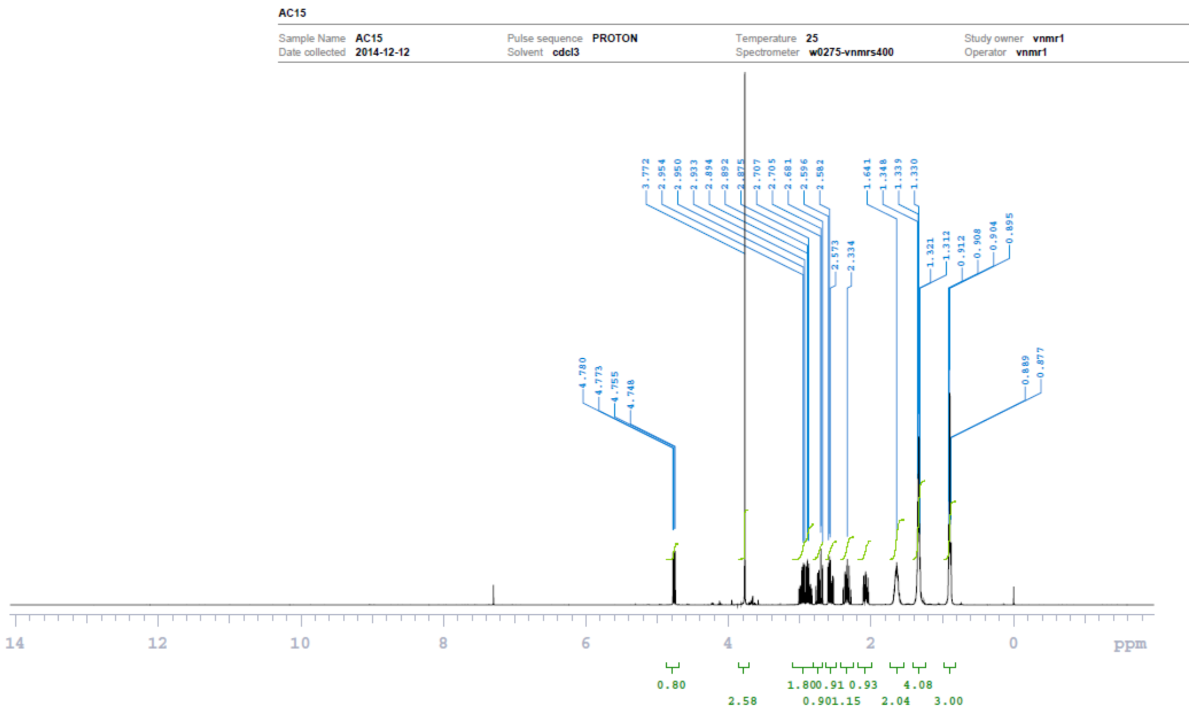
AC15
Sample Name AC15
Date collected 2014-12-12
Pulse sequence PROTON
Solvent cdcl3
Temperature 25
Spectrometer w0275-vnmrs400
Study owner vnmr1
Operator vnmr1
ppm

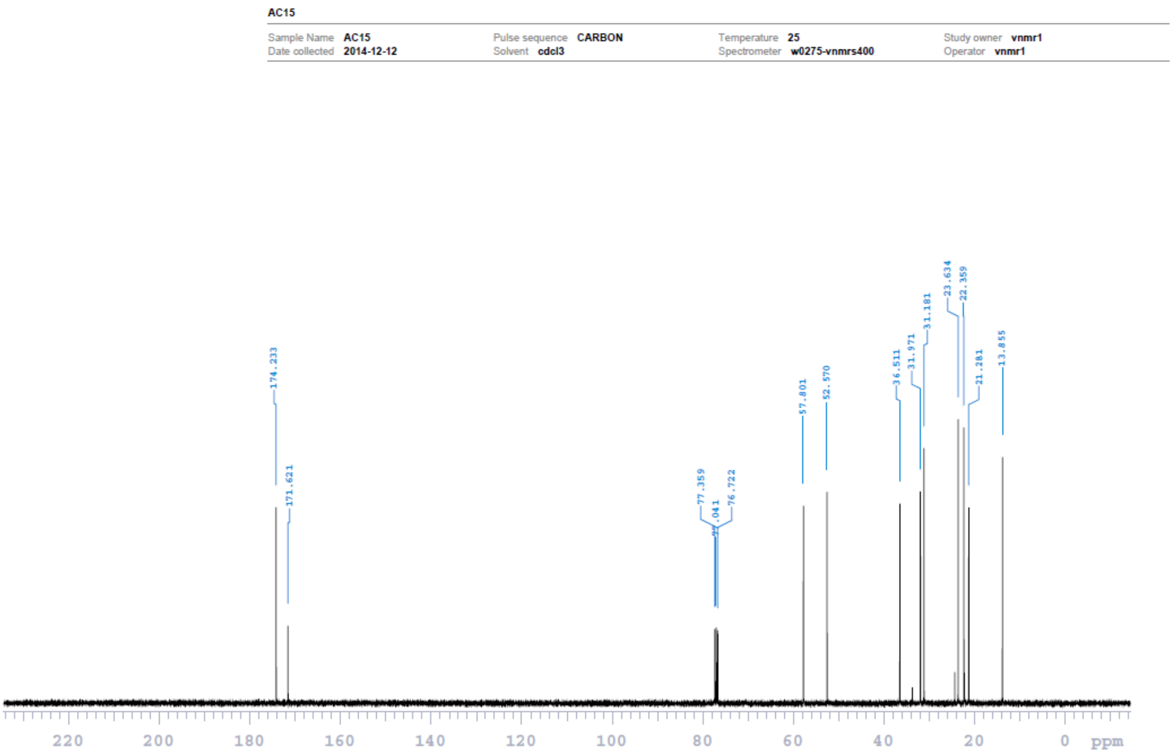
AC15
Sample Name AC15
Date collected 2014-12-12
Pulse sequence CARBON
Solvent cdcl3
Temperature 25
Spectrometer w0275-vnmrs400
Study owner vnmr1
Operator vnmr1
ppm

<u>*L-Methyl pyroglutamate* (**M9**)</u>

L-Methylpyroglutamate (**M9**) was obtained by esterification of pyroglutamic acid following a described procedure.[ii] reaction is carried out in a mixture of refluxing methanol and chloroform which, in the presence of a catalytic amount of methanesulphonic acid, via a soxhlet filled with 3Å molecular sieves, allows the removal of water formed during the reaction (Reaction 2). Methyl ester **M9** was thus obtained in quantitative yield with the same properties as described in the literature.[2.]

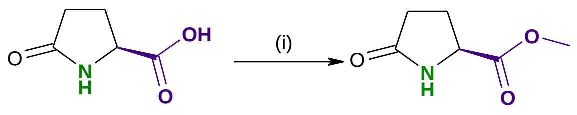

pyroglutamic acid **M9**

Reaction 2. *Reagents and conditions: (i) methanesulfonic acid, molecular sieves 3Å, MeOH/$CHCl_3$, reflux, 98%.*

General procedure for the synthesis of vinylogous acylureas M10 and M20 and of bicyclic molecule M19 (Reaction 3).

Isocyanates (1 equiv.) dissolved in toluene were added dropwise with a syringe to a stirred solution of methyl 5-(2-methoxy-2-oxoethylidene)prolinate (**M21**) (1 equiv.) in refluxed toluene (20 mL). The mixture was stirred under nitrogen atmosphere at reflux for 48 h. After cooling to room temperature, methanol was added. The mixture was concentrated and purified by flash chromatography (elution with *n*-heptane/ethyl acetate: 80/20 → *n*-heptane/ethyl acetate: 0/100) to afford the products **M10**, **M19** and **M20** (Reaction 3).

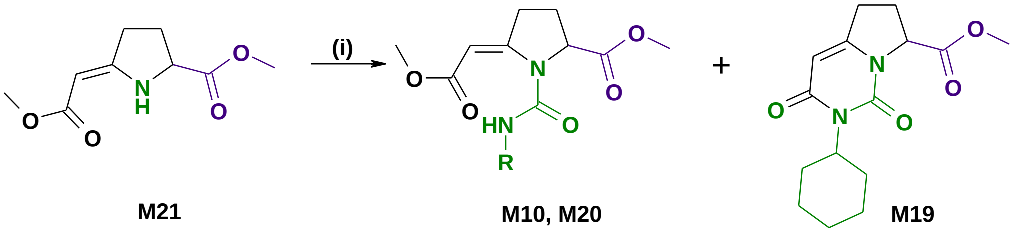

M21 **M10, M20** **M19**

Reaction 3. Reagents and conditions: *(i) 1 eq. R-NCO, toluene, reflux, 48 h.*

Methyl 1-[(butylamino)carbonyl]-5-(2-methoxy-2-oxoethylidene) prolinate (**M10**)

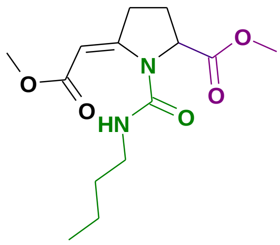

The general procedure was followed using methyl 5-(2-methoxy-2-oxoethylidene)prolinate (**M21**) (3.00 g, 15.0 mmol, 1 equiv.) in toluene (20mL) and *n*-butyl isocyanate (1.49 g, 15.0 mmol, 1 equiv.) and mixture was heated at reflux under nitrogen atmosphere for 48 hours. After cooling to room temperature, methanol (20 mL) was added. The residue was concentrated and separated by flash chromatography on silica column (elution with *n*-heptane/ethyl acetate: 80/20 → *n*-heptane/ethyl acetate: 0/100) to generate pure product **M10** as yellow oil in 24% yield, R_f (EtOAc/*n*-heptane 50/50) = 0.3.

IR ν cm^{-1}: 3311, 2955, 1743, 1655, 1599, 1435, 1209, 1073, 801, 554.

^{1}H NMR (400 MHz, $CDCl_3$) δ ppm: 0.91 (t, *J* = 7.2 Hz, 3H, CH_3), 1.32-1.42 (m, 2H, CH_2), 1.48-1.56 (m, 2H, CH_2), 2.01-2.19 (m, 1H, $CH_2C\mathit{H_2}CH$), 2.24-2.35 (m, 1H, $CH_2C\mathit{H_2}CH$), 3.09-3.22 (m, 2H, $C\mathit{H_2}CH_2CH$), 3.24-3.32 (m, 2H, CH_2), 3.69 (s, 3H, $OC\mathit{H_3}$), 3.74 (s, 3H, $OC\mathit{H_3}$), 4.45 (dd, *J* = 9.2, 5.3 Hz, 1H, $CH_2CH_2C\mathit{H}$), 9.07 (s, 1H, C*H*), 11.79 (s, 1H, N*H*).

^{13}C NMR (100 MHz, $CDCl_3$) δ ppm: 13.8 (CH_3), 20.3 (CH_2), 25.5 (CH_2), 31.7 (CH_2), 35.1 (CH_2), 38.8 (CH_2), 50.6 (OCH_3), 52.6 (OCH_3), 60.9 (CH), 88.1(CH), 169.8 (2C), 171.6 (C), 173.5 (C).

Anal. calcd for $\mathbf{C_{14}H_{22}N_2O_5}$ (298.34 g/mol): C, 56.36; H, 7.43; N, 9.39%. Found: C, 56.51; H, 7.39; N, 9.70%.

LogP: 0.70 ± 0.61.

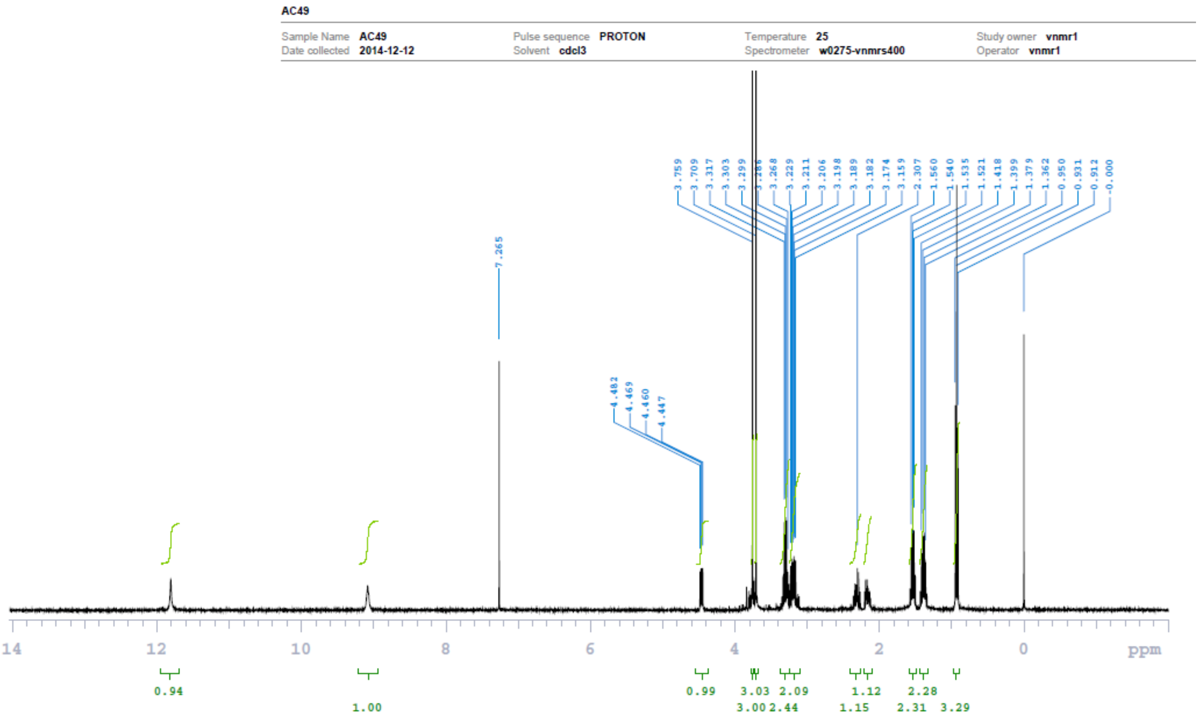
AC49
Sample Name AC49
Date collected 2014-12-12
Pulse sequence PROTON
Solvent cdcl3
Temperature 25
Spectrometer w0275-vnmrs400
Study owner vnmr1
Operator vnmr1
7.265
4.482 4.469 4.460 4.447
3.759 3.709 3.317 3.303 3.299 3.268 3.229 3.211 3.206 3.198 3.189 3.182 3.174 3.159 2.307 1.560 1.540 1.535 1.521 1.418 1.399 1.379 1.362 0.950 0.931 0.912 -0.000
14 12 10 8 6 4 2 0 ppm
0.94 1.00 0.99 3.03 3.00 2.09 2.44 1.12 1.15 2.28 2.31 3.29

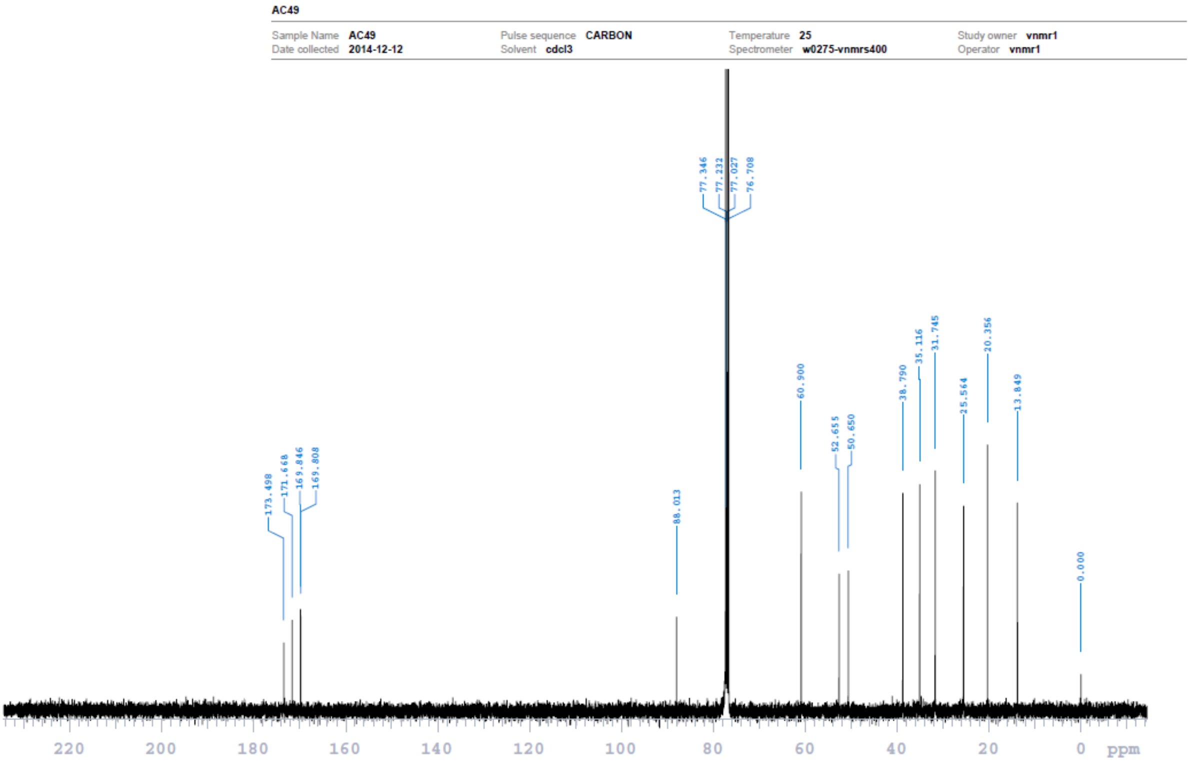
AC49
Sample Name AC49
Date collected 2014-12-12
Pulse sequence CARBON
Solvent cdcl3
Temperature 25
Spectrometer w0275-vnmrs400
Study owner vnmr1
Operator vnmr1
173.498 171.668 169.846 169.808
88.013
77.346 77.232 77.027 76.708
60.900 52.655 50.650 38.790 35.116 31.745 25.564 20.356 13.849 0.000
220 200 180 160 140 120 100 80 60 40 20 0 ppm

Methyl 1-[(cyclohexylamino)carbonyl]-5-(2-methoxy-2-oxoethylidene)prolinate (**M20**) and *methyl 2-cyclohexyl-1,3-dioxo-1,2,3,5,6,7-hexahydropyrrolo[1,2-c]pyrimidine-7-carboxylate* (**M19**)

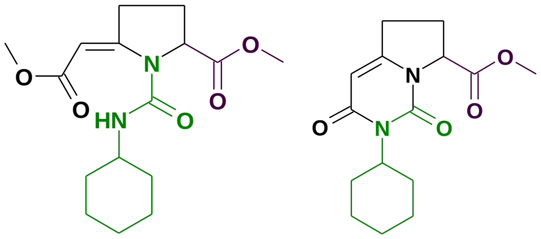

M20 **M19**

The general procedure was followed using methyl 5-(2-methoxy-2-oxoethylidene)prolinate (**M21**) (3.00 g, 15.0 mmol, 1 equiv.) in toluene (20mL) and cyclohexyl isocyanate (1.88 g, 15.0 mmol, 1 equiv.) and heated at reflux under nitrogen atmosphere for 48 hours. After cooling to room temperature, methanol (20 mL) was added. The residue was concentrated and separated by flash chromatography on silica column (elution with *n*-heptane/ethyl acetate: 80/20 → *n*-heptane/ethyl acetate: 0/100) to generate pure products **M20** and **M19**.

Product **M20**: white solid; 56% yield, mp 120-122 °C, R_f (EtOAc/*n*-heptane 50/50) = 0.33.

IR ν cm^{-1}: 2935, 1741, 1703, 1649, 1524, 1412, 1199, 1100, 796, 552.

^{1}H NMR (400 MHz, $CDCl_3$) δ ppm: 1.18-1.33 (m, 4H, $2CH_2$), 1.33-1.46 (m, 2H, CH_2), 1.66-1.77 (m, 2H, CH_2), 1.86-1.97 (m, 2H, CH_2), 2.11-2.21 (m, 1H, CH_2CH_2CH), 2.25-2.37 (m, 1H, CH_2CH_2CH), 3.10-3.28 (m, 2H, CH_2CH_2CH), 3.71 (s, 3H, OCH_3), 3.76 (s, 3H, OCH_3), 3.78-3.90 (m, 1H, CH), 4.46 (dd, J = 11.4, 6.0 Hz, 1H, CH_2CH_2CH), (d, J = 7.6 Hz, 1H, C*H*), 11.80 (s, 1H, N*H*).

^{13}C NMR (100 MHz, $CDCl_3$) δ ppm: 24.8 (CH_2), 25.5 (CH_2), 25.8 (CH_2), 33.1 ($2CH_2$), 35.1 ($2CH_2$), 47.5 (CH), 50.6 (CH_3), 52.6 (CH_3), 60.9 (CH), 88.0 (CH), 168.9 (C), 169.8 (C), 171.6 (C), 173.5 (C).

Anal. calcd for $C_{16}H_{24}N_2O_5$ (324.38 g/mol): C, 59.24; H, 7.46; N, 8.64%. Found: C, 59.43; H, 7.69; N, 9.00%.

LogP: 0.70 ± 0.61.

AC65

Sample Name AC65	Pulse sequence PROTON	Temperature 25	Study owner vnmr1
Date collected 2015-01-27	Solvent cdcl3	Spectrometer w0275-vnmrs400	Operator vnmr1

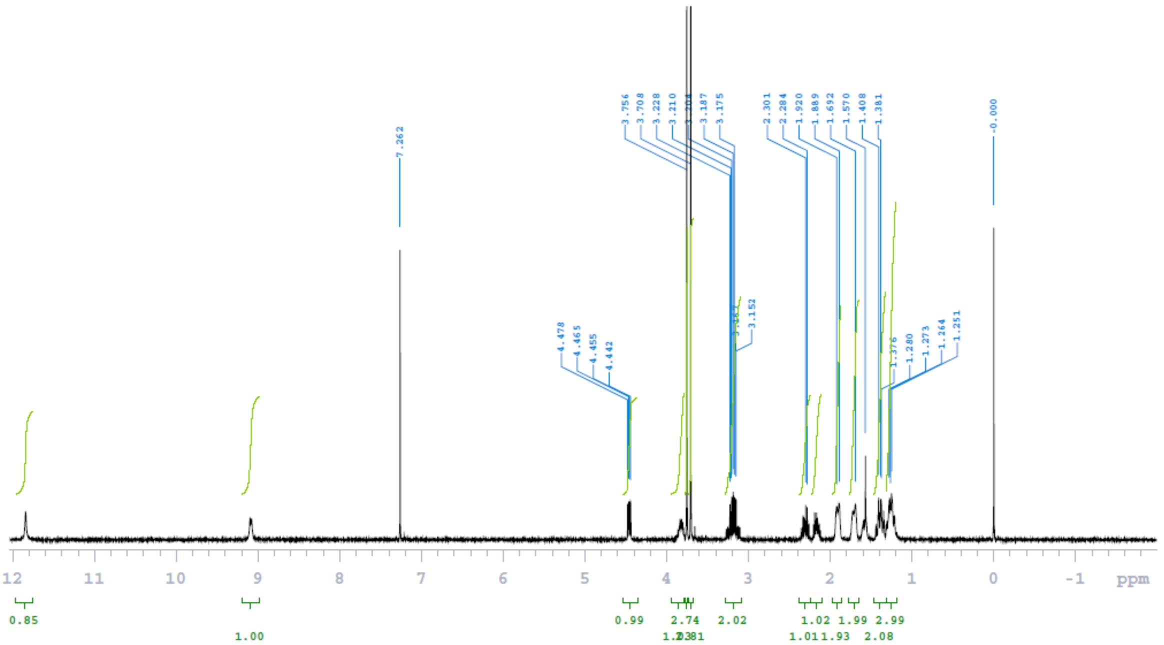

AC65

Sample Name AC65	Pulse sequence CARBON	Temperature 25	Study owner vnmr1
Date collected 2015-01-30	Solvent cdcl3	Spectrometer w0275-vnmrs400	Operator vnmr1

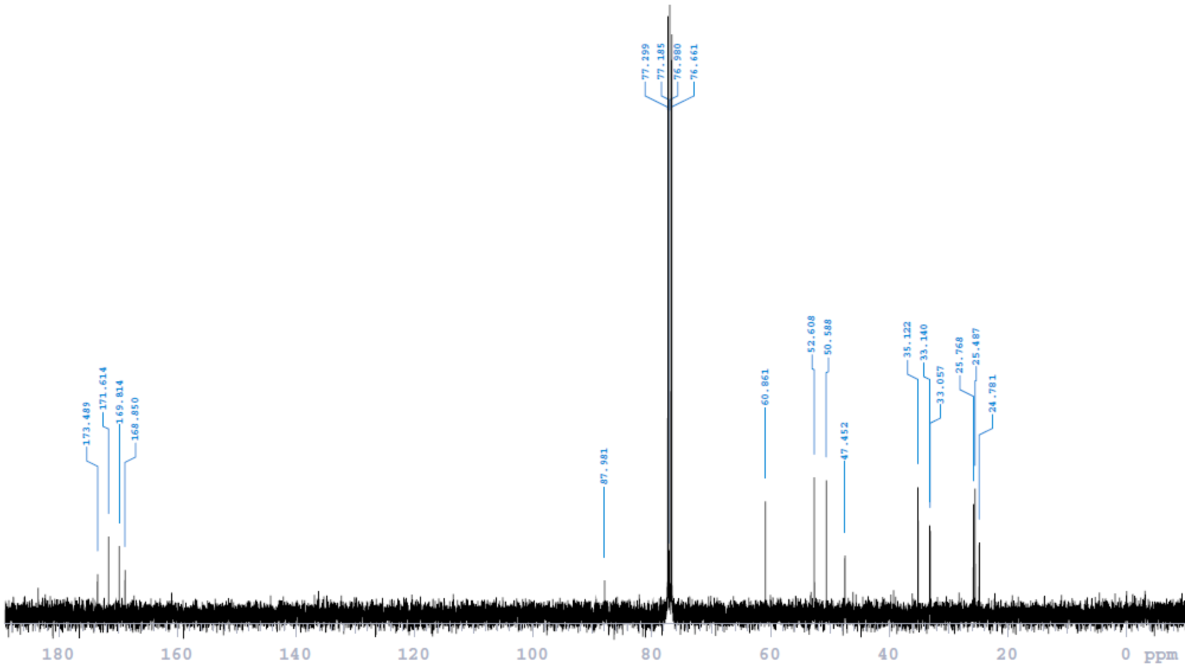

Product **M19:** yellow oil; 10% yield, R_f (EtOAc/*n*-heptane 50/50) = 0.6;

IR ν cm^{-1}: 2933, 1709, 1637, 1363, 1150, 848, 601, 421.

^{1}H NMR (400 MHz, $CDCl_3$) δ ppm: 1.17-1.41 (m, 4H, $2CH_2$), 1.68-1.74 (m, 2H, CH_2), 1.74-1.94 (m, 4H, $2CH_2$), 2.38-2.44 (m, 1H, $CH_2C\mathit{H_2}CH$), 2.89-2.94 (m, 3H, $C\mathit{H_2}C\mathit{H_2}CH$), 3.68 (s, 3H, $OC\mathit{H_3}$), 4.54-4.63 (m, 1H, CH), 4.72 (dd, *J* = 9.0, 5.3 Hz, 1H, $CH_2CH_2C\mathit{H}$), 5.76 (s, 1H, C*H*).

^{13}C NMR (100 MHz, $CDCl_3$) δ ppm: 22.4 ($2CH_2$), 26.7 (CH_2), 30.9 ($2CH_2$), 31.6 (CH_2), 35.1 ($2CH_2$), 34.9 (CH_2), 52.5 (CH_3), 54.6 (CH), 59.0 (CH), 100.9 (CH), 168.9 (C), 150.2 (C), 162.0 (C), 164.4 (C), 172.5 (C).

Anal. calcd for $\mathbf{C_{15}H_{20}N_2O_4}$ (292.34 g/mol): C, 61.63; H, 6.90; N, 9.58%. Found: C, 61.90; H, 7.11; N, 9.94%.

LogP: 2.62 ± 0.40.

Methyl 1-[(butylamino)carbonyl]-4-[(dimethylamino)methylene]-5-oxoprolinate (**M11**)

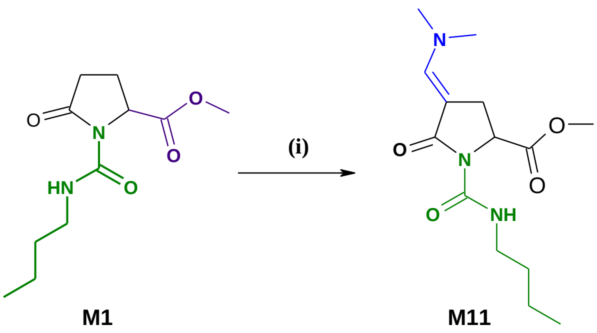

Reaction 4. *Reagents and conditions*: *(i)* Bredereck's reagent *(2 equiv.), toluene, reflux, 4h.*

A mixture of compound **M1** (1 equiv.), and Bredereck's reagent (3 equiv.) was heated at reflux for 4 h (Reaction 4). Volatile components were evaporated *in vacuo*, dichloromethane was then added (200 mL) and the solution was washed with distilled water. The organic phase was dried with anhydrous magnesium sulfate, filtered, and the filtrate concentrated *in vacuo*. The residue was concentrated and separated by flash chromatography on silica column (elution with *n*-heptane/ethyl acetate: 80/20 → *n*-heptane/ethyl acetate: 0/100) to generate pure product **M11** as a white solid in 25% yield; mp 117-118 °C, R_f (EtOAc/*n*-heptane 50/50) = 0.56.

IR ν cm^{-1}: 3311, 2960, 1752, 1692, 1599, 1528, 1437, 1334, 1248, 1115, 630, 459.

^{1}H NMR (400 MHz, $CDCl_3$) δ ppm: 0.92 (t, *J* = 7.3 Hz, 3H, CH_3), 1.33-1.42 (m, 2H, CH_2), 1.50-1.57 (m, 2H, CH_2), 2.89 (dd, *J* = 15.0, 2.9 Hz, 1H, CH_2CH), 3.03 (s, 6H, $2CH_3$, $N(CH_3)_2$), 3.22-3.37 (m, 3H, CH_2 + CH_2CH), 3.76 (s, 3H, OCH_3), 4.72 (dd, *J* = 10.8, 3.5 Hz, 1H, CH_2CH), 7.07 (s, 1H, $CHN(CH_3)_2$), 8.63 (s, 1H, N*H*).

^{13}C NMR (100 MHz, $CDCl_3$) δ ppm: 13.7 (CH_3), 20.0 (CH_2), 26.0 (CH_2), 31.8 ($2CH_2$), 39.4 ($2CH_3$), 52.4 (CH_3), 55.0 (CH), 91.5 (C), 146.1 (CH), 153.8 (C), 172.0 (C), 172.7 (C).

Anal. calcd for **$C_{14}H_{23}N_3O_4$** (297.36 g/mol): C, 56.55; H, 7.80; N, 14.13%. Found: C, 56.90; H, 8.11; N, 14.53%.

LogP: 0.94 ± 0.68.

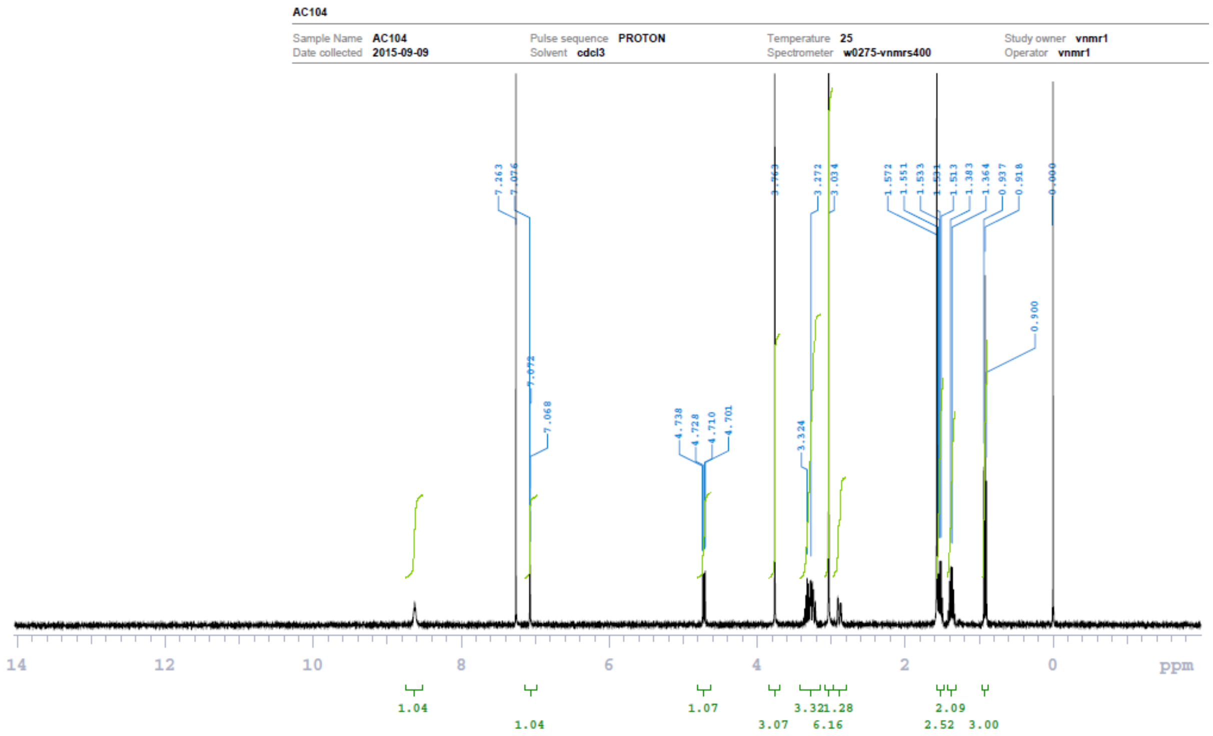

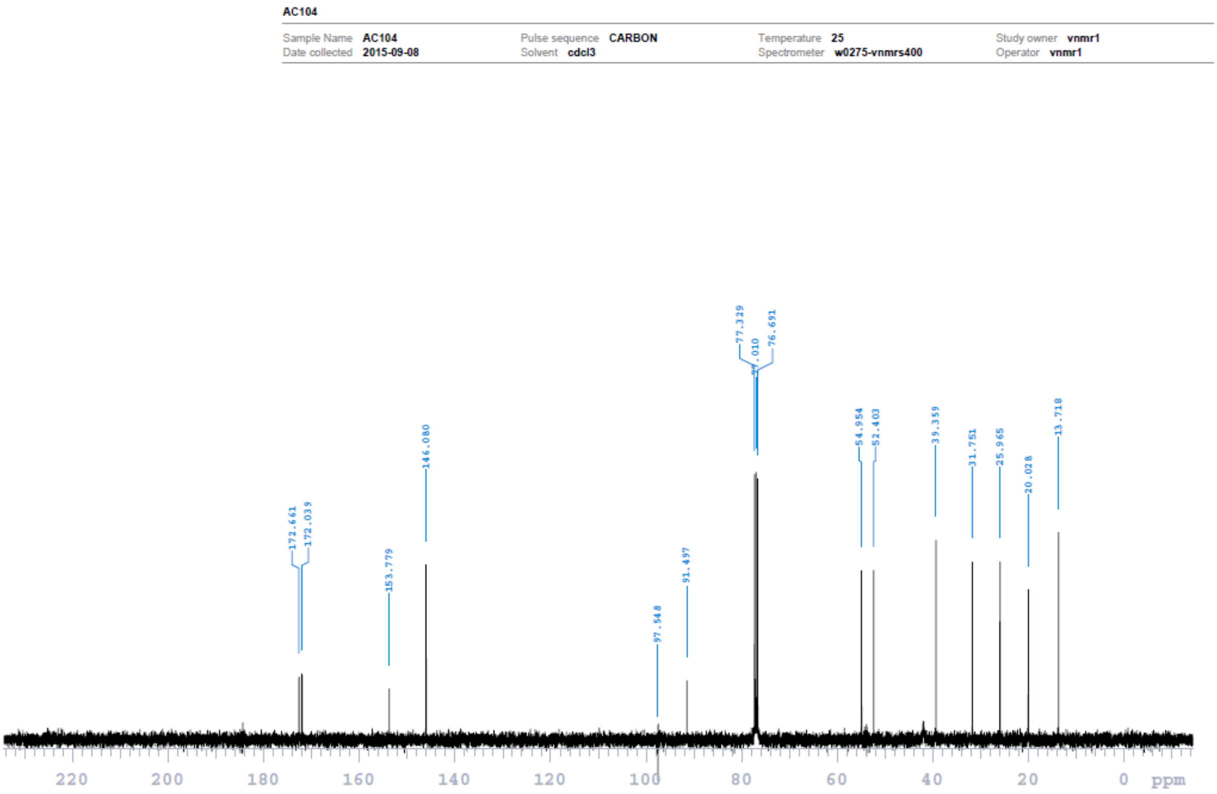

Methyl 1-[(butylamino) arbonyl]-4-(hydroxyimino)-5-oxoprolinate (**M12**).

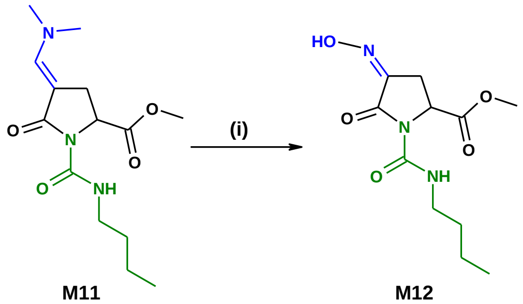

Reaction 5. *Reagents and conditions*: *(i) CH_3COOH, $NaNO_2$, H_2O, 0-5 °C, 3h.*

A cold solution (0-5°C) of sodium nitrite (460 mg, 66.6 mmol, 1 equiv.) in water (3 mL) was added to a stirred solution of enamine **M11** (500 mg, 16.8 mmol, 4 equiv.) in acetic acid for 3 h (Reaction 5). The solution was neutralized with saturated aqueous $NaHCO_3$ (50 mL) and was extracted with dichloromethane (2×100 mL). The combined organic layers were dried over $MgSO_4$ and then evaporated, to generate pure product **M12** as white powder in 83% yield; mp (Et_2O) 119–122°C; R_f (EtOAc/*n*-heptane 50/50) = 0.20.

IR ν cm^{-1}: 3244, 2930, 1752, 1715, 1651, 1524, 1386, 1244, 1179, 950, 730, 532.

^{1}H NMR (400 MHz, $CDCl_3$) δ ppm: 0.94 (t, *J* = 7.3 Hz, 3H, CH_3), 1.33-1.43 (m, 2H, CH_2), 1.53-1.60 (m, 2H, CH_2), 2.91 (dd, *J* = 19.8; 3.0 Hz, 1H, CH_2CH), 3.13 (dd, *J* = 19.8; 9.9 Hz, 1H, CH_2CH), 3.27-3.41 (m, 2H, CH_2), 3.80 (s, 3H, OCH_3), 4.88 (dd, *J* = 9.8, 3.0 Hz, 1H, CH_2C*H*), 8.32 (s, 1H, N*H*), 9.15 (s, 1H, NO*H*).

^{13}C NMR (100 MHz, $CDCl_3$) δ ppm: 13.7 (CH_3), 19.9 (CH_2), 24.4 (CH_2), 31.5 (CH_2), 39.9 (CH_2), 53.0 (CH_3), 53.8 (CH), 150.2 (C), 151.8 (C), 163.8 (C), 170.9 (C).

Anal. calcd for **$C_{11}H_{17}N_3O_5$** (271.28 g/mol): C, 48.70; H, 6.32; N, 15.49%. Found: C, 49.06; H, 6.67; N, 15.90%.

LogP: -0.38 ± 0.66.

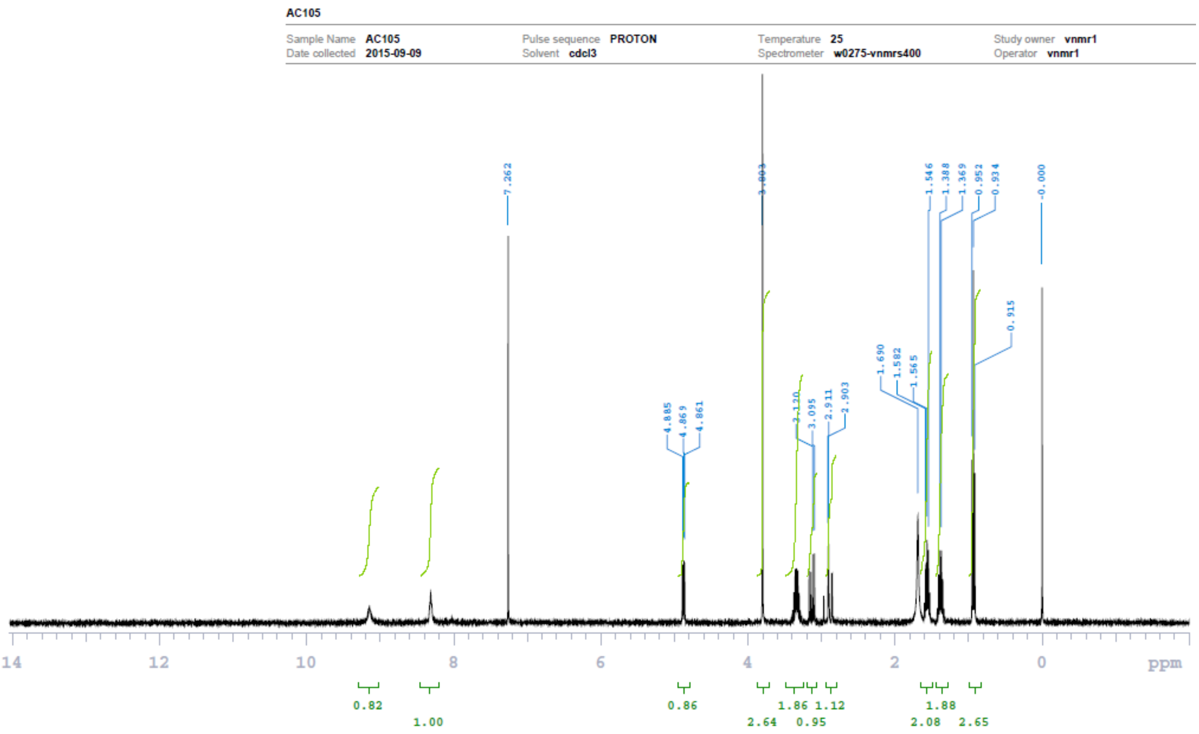

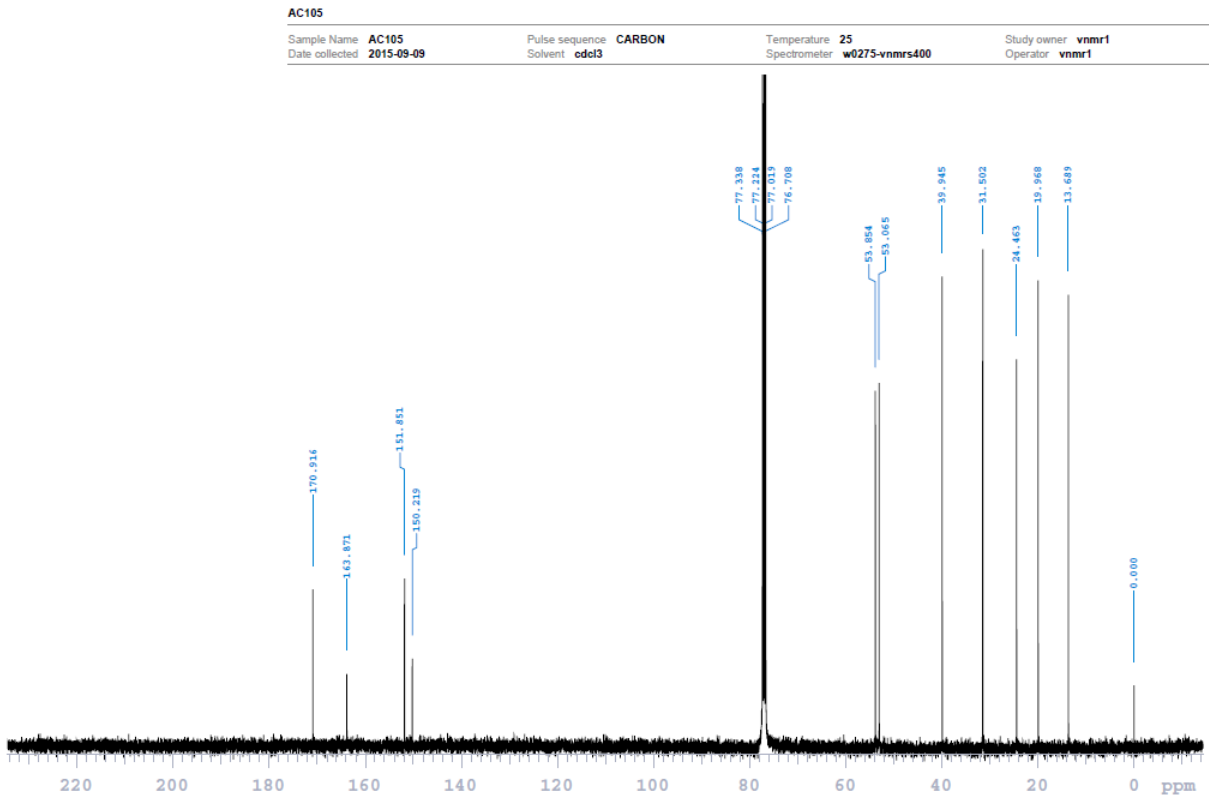

Methyl 3-[(butylamino)carbonyl]-2-oxo-1,3-oxazolidine-4-carboxylate (**M13**)

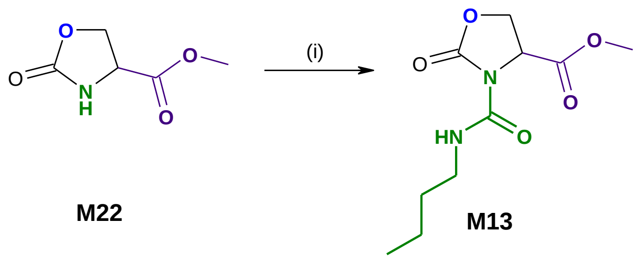

Reaction 6. *Reagents and conditions: (i) 1.2 eq. n-butyl isocyanate, toluene, reflux, 24h.*

n-Butyl isocyanate (1.64 g, 16.5 mmol, 1.2 equiv.) was added to a mixture of methyl *DL*-2-oxo-1, 3-oxazolidine-4-carboxylate **M22** (2.00 g, 13.8 mmol, 1 equiv.) in toluene (20mL) and the medium was heated at reflux under nitrogen atmosphere for 24 hours (Reaction 6). After cooling to room temperature, methanol (20 mL) was added. The residue was concentrated and separated by flash chromatography on silica column (elution with *n*-heptane/ethyl acetate: 80/20 → *n*-hexane/ethyl acetate: 0/100) to generate pure product **M13** a yellow oil in 54% yield, R_f (EtOAc/*n*-heptane 50/50) = 0.36.

IR ν cm^{-1}: 3358, 2958, 1753, 1700, 1538, 1396, 1206, 1101, 763, 609.

^{1}H NMR (400 MHz, $CDCl_3$) δ ppm: 0.93 (t, *J* = 7.4 Hz, 3H, $CH_2CH_2CH_2C\mathit{H}_3$), 1.32-1.42 (m, 2H, $CH_2CH_2C\mathit{H}_2CH_3$), 1.51-1.59 (m, 2H, $CH_2C\mathit{H}_2CH_2CH_3$), 3.25-3.37 (m, 2H, $C\mathit{H}_2CH_2CH_2CH_3$), 3.83 (s, 3H, $OC\mathit{H}_3$), 4.34 (dd, *J* = 9.4, 3.9 Hz, 1H, $SC\mathit{H}_2CH$), 4.56 (t, *J* = 9.4 Hz, 1H, $SC\mathit{H}_2CH$), 4.94 (dd, *J* = 9.4, 3.9 Hz, 1H, $SCH_2C\mathit{H}$), 7.68 (br s, 1H, N*H*).

^{13}C NMR (100 MHz, $CDCl_3$) δ ppm: 13.6 (CH_3), 19.9 (CH_2), 31.5 (CH_2), 39.9 (CH_2), 53.2 (CH_3), 55.2 (CH), 64.8 (CH_2), 150.7 (C), 154.7 (C), 169.5 (C).

Anal. calcd for **$C_{10}H_{16}N_2O_5$** (244.25 g/mol): C, 49.18; H, 6.60; N, 11.47%. Found: C, 49.27; H, 6.22; N, 11.90%.

LogP: -0.20 ± 0.65.

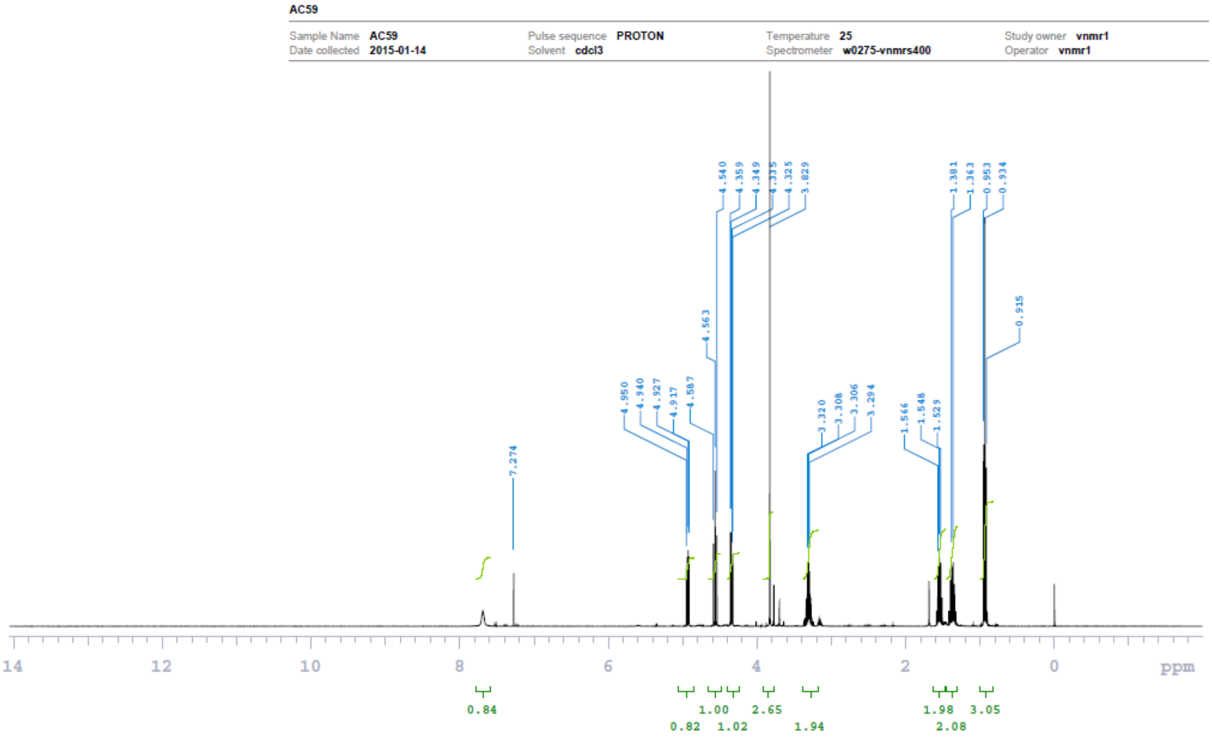

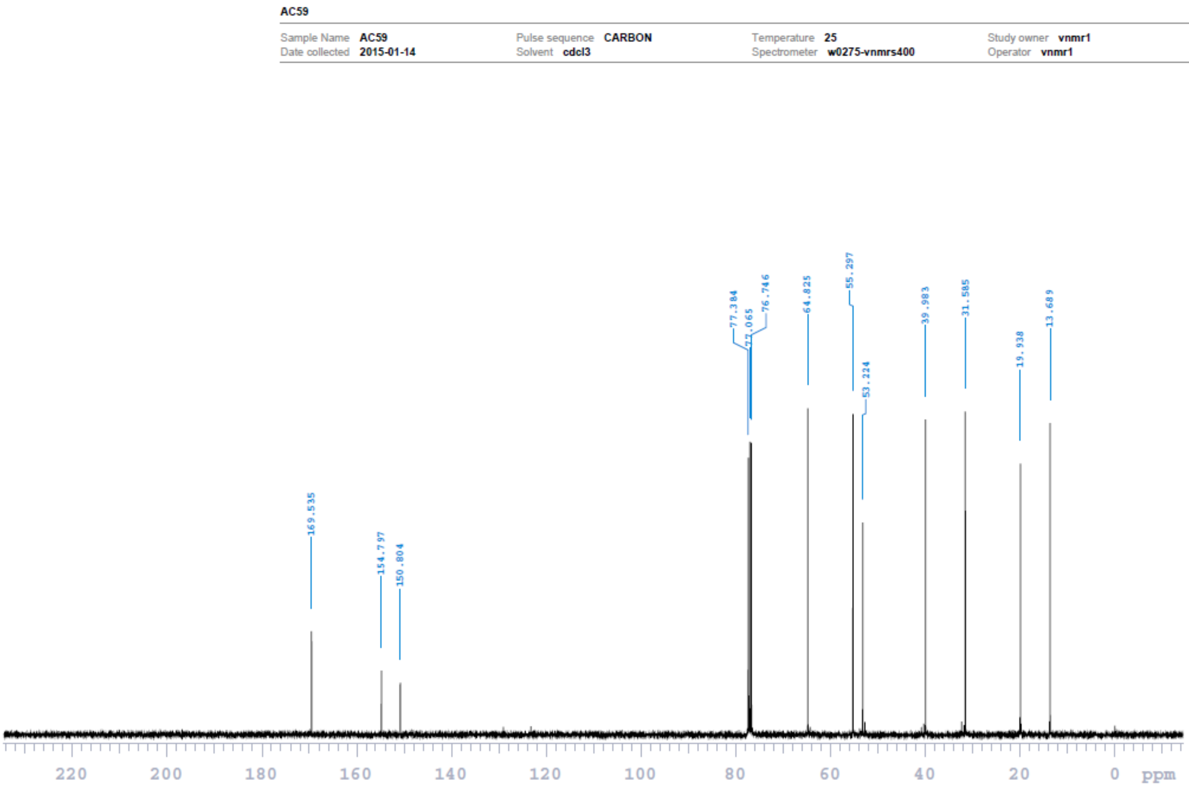

1-Butylcarbamoyl-5-oxo-pyrrolidine-2-carboxylic acid (**M14**)

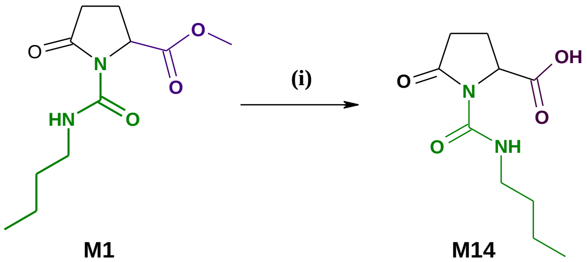

Reaction 7. *Reagents and conditions: (i) 1.5 equiv.* Cs_2CO_3*,* H_2O*, reflux, 6h, 89%.*

Methyl 1-[(butylamino)carbonyl]-5-oxoprolinate (**M1**) (1 equiv.) was mixed with a 2N aqueous solution of cesium carbonate (Cs_2CO_3) (1.5 equiv.) and then refluxed for 6 hours (Reaction 7). After complete solubilization and cooling the medium at rt, concentrated HCl was added till a white precipitate formed. The precipitate was washed with water and dried to provide pure carboxylic acid **M14** as a white solid in 89% yield.

^{1}**H NMR** (400 MHz, DMSO-d$_6$) δ ppm: 0.85 (t, *J* = 7.4 Hz, 3H, $CH_2CH_2CH_2CH_3$), 1.17-1.27 (m, 2H, $CH_2CH_2CH_2CH_3$), 1.41-1.49 (m, 2H, $CH_2CH_2CH_2CH_3$), 1.64-1.74 (m, 1H, $CH_2CH_2CH_2CH_3$), 1.87-1.97 (m, 1H, $CH_2CH_2CH_2CH_3$), 2.25-2.32 (m, 2H, CH_2CH_2CH), 3.20

(dt, J = 7.08, 2.36 Hz, 2H, CH_2CH_2CH), 4.05 (t, J = 6.68 Hz, 1H, CH_2CH_2CH), 8.22 (s, 1H, N*H*), 12.23 (br s, 1H, CO_2H).

^{13}C NMR (100 MHz, DMSO-d_6) δ ppm: 13.9 (CH_3), 19.8 (CH_2), 27.4 (CH_2), 29.5 (CH_2), 30.0 (CH_2), 37.7 (CH_2), 55.8 (CH), 157.2 (C), 174.1 (C), 174.5 (C).

Anal. calcd for **$C_{10}H_{16}N_2O_4$** (228.25 g/mol): C, 52.62; H, 7.07; N, 12.27%. Found: C, 52.19; H, 6.79; N, 12.49%.

LogP: -1.24 ± 0.62.

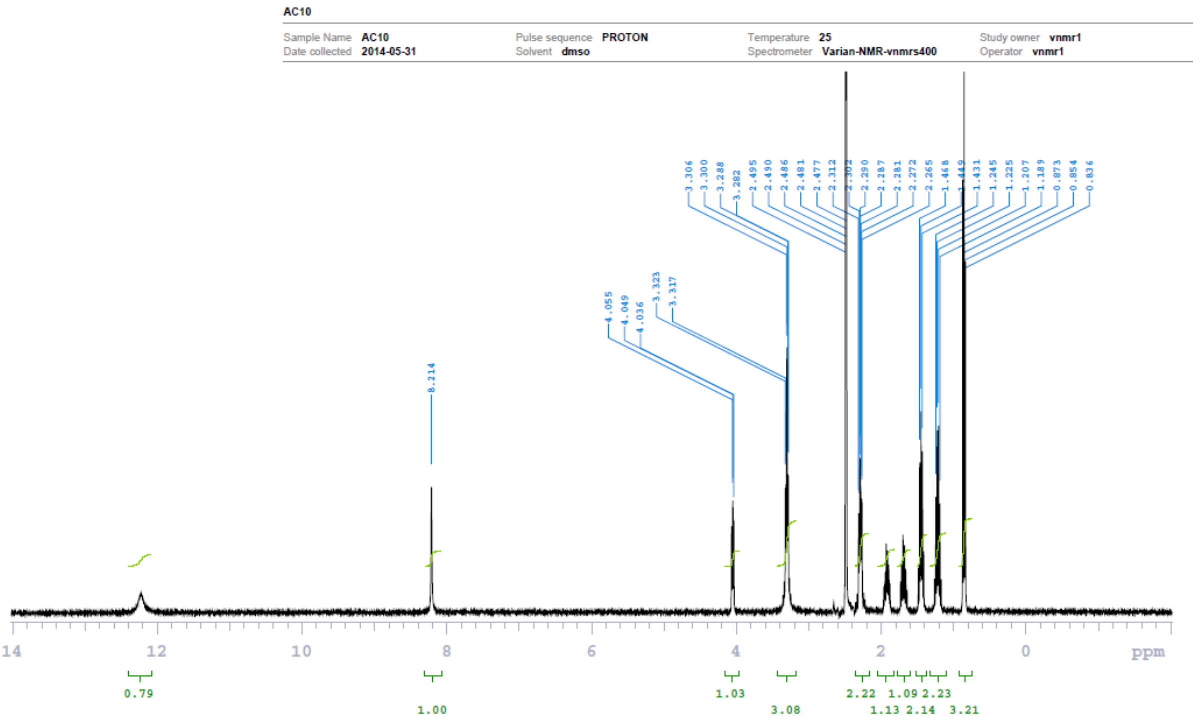

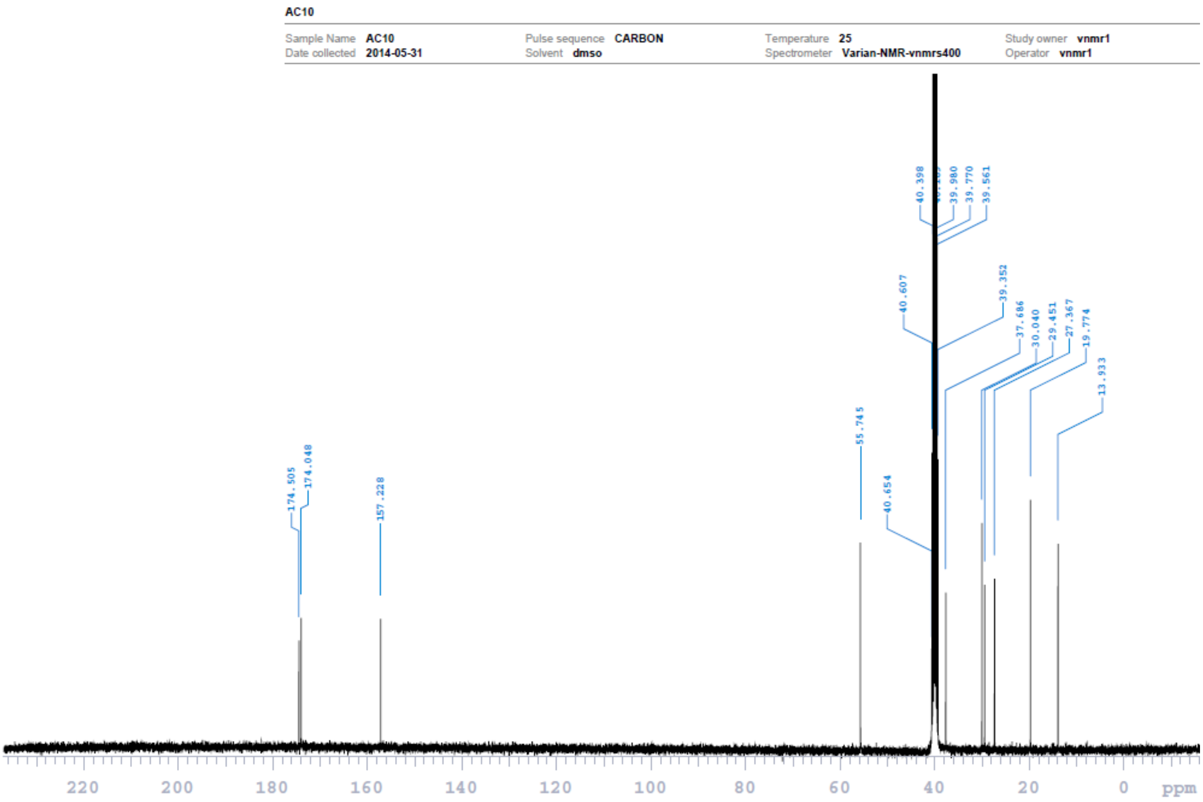

General procedure for the synthesis of derivatives M15, M16, M17 and M18, analogues of M1 with different substitutions in position 5 (Reaction 8).

To a stirred solution of substrate (1 equiv.) in refluxed toluene (20 mL), was added dropwise with a syringe *n*-butyl isocyanate (1-1.2 equiv.) dissolved in toluene. The mixture was stirred under nitrogen atmosphere at reflux for 24-48 h (Reaction 8). After cooling to room temperature, methanol was added. The mixture was concentrated and purified by flash chromatography on silica pre-packed column (elution with *n*-heptane/ethyl acetate: 80/20 → *n*-heptane/ethyl acetate: 0/100) to afford the products.

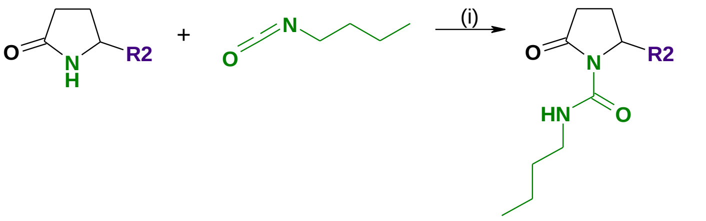

M15-M16-M17-M18

Reaction 8. *Reagents and conditions: (i) 1-1.2 equiv. n-C_4H_9NCO, toluene, reflux, 24-48 h.*

<u>Allyl 1-[(butylamino)carbonyl]-5-oxoprolinate</u> **<u>(M15)</u>**

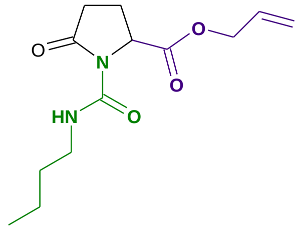

The general procedure was followed using allyl 5-oxoprolinate[iii] (3.0 g, 17.73 mmol, 1 equiv.) in toluene (20mL) and *n*-butyl isocyanate (2.26 g, 18.0 mmol, 1.02 equiv.). The mixture was heated at reflux under nitrogen atmosphere for 48 hours. After cooling to room temperature, methanol (20 mL) was added. The residue was concentrated and separated by flash chromatography on silica column (elution with *n*-heptane/ethyl acetate: 80/20 → *n*-heptane/ ethyl acetate: 0/100) to generate pure product **M15** a yellow oil in 48% yield, R_f (EtOAc/*n*-heptane 50/50) = 0.73.

IR ν cm^{-1}: 3317, 2960, 1717, 1539, 1459, 1368, 1255, 1193, 734, 595.

^{1}H NMR (400 MHz, $CDCl_3$) δ ppm: 0.90 (t, J = 7.2 Hz, 3H, CH_3), 1.29-1.41 (m, 2H, CH_2), 1.45-1.57 (m, 2H, CH_2), 2.07-2.18 (m, 1H, CH_2CH_2CH), 2.32-2.37 (m, 1H, CH_2CH_2CH), 2.46-2.53 (m, 1H, CH_2CH_2CH), 2.76 (s, 2H, CH_2), 2.92-3.01 (m, 1H, CH_2CH_2CH), 3.21-3.33 (m, 4H, $NHCH_2CH_2CH_2CH_3$ + OCH_2), 4.70 (dd, *J* = 8.6, 1.3 Hz, 1H, CH_2CH_2CH), 6.7 (br s, 1H, C*H*), 8.47 (br s, 1H, N*H*).

^{13}C NMR (100 MHz, $CDCl_3$) δ ppm: 13.7 (CH_3), 20.0 (CH_2), 28.4 (CH_2), 30.6 (CH_2), 32.2 (CH_2), 38.3 (CH_2), 59.3 (CH), 65.4 (CH_2), 118.3 (CH_2), 132.2 (CH), 156.2 (C), 169.6 (C), 169.9 (C).

Anal. calcd for $\mathbf{C_{13}H_{20}N_2O_4}$ (268.32 g/mol): C, 58.19; H, 7.51; N, 10.44%. Found: C, 58.26; H, 7.39; N, 10.68%.

LogP: 0.16± 0.63.

AC67

Sample Name **AC67**	Pulse sequence **PROTON**	Temperature **25**	Study owner **vnmr1**
Date collected **2015-09-16**	Solvent **cdcl3**	Spectrometer **w0275-vnmrs400**	Operator **vnmr1**

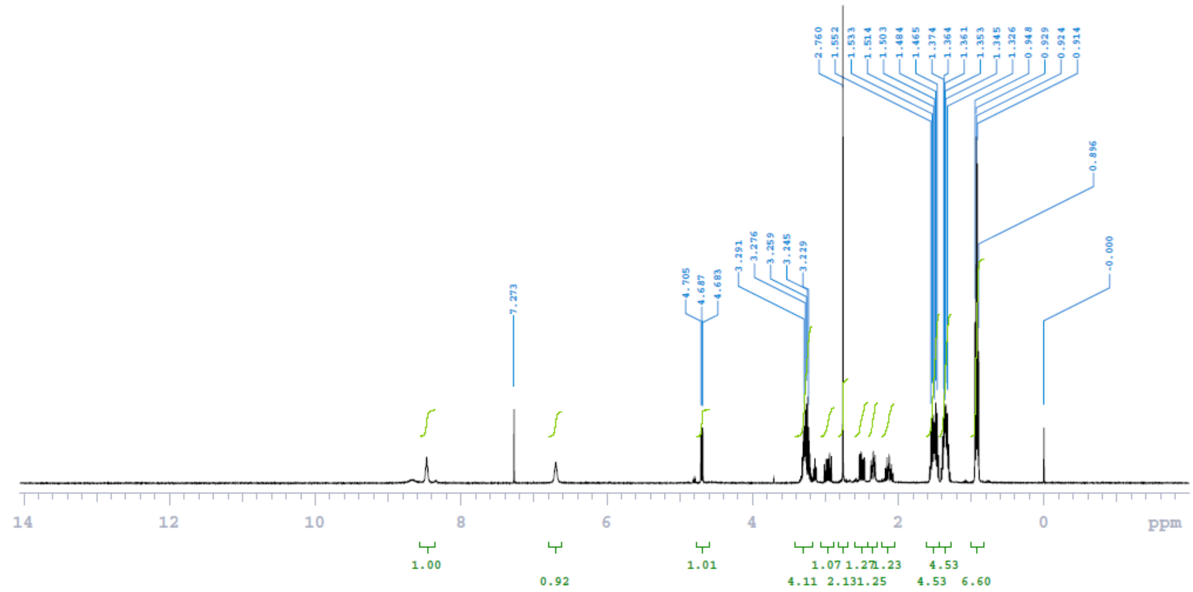

AC67

Sample Name **AC67**	Pulse sequence **CARBON**	Temperature **25**	Study owner **vnmr1**
Date collected **2015-09-16**	Solvent **cdcl3**	Spectrometer **w0275-vnmrs400**	Operator **vnmr1**

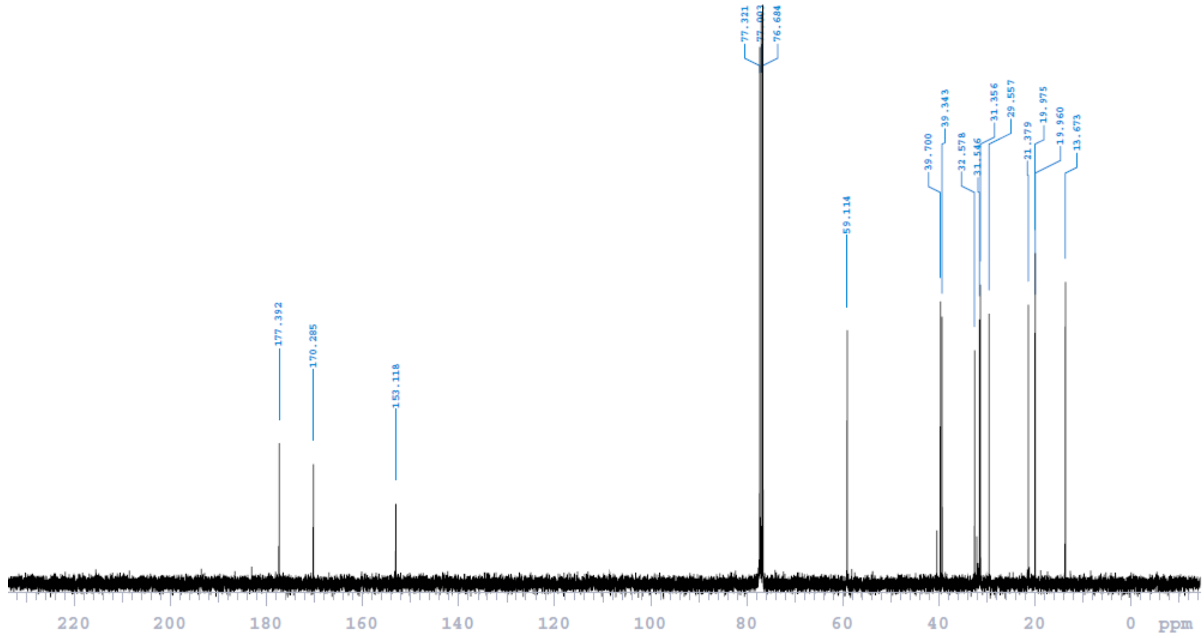

N-Butyl-2-cyano-5-oxopyrrolidine-1-carboxamide **(M16)**

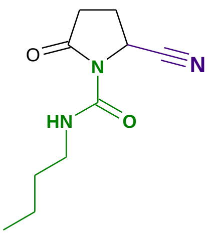

The general procedure was followed using 5-oxopyrrolidine-2-carbonitrile[iv] (3.0 g, 27.2 mmol, 1 equiv.) in toluene (20mL) and *n*-butyl isocyanate (2.7 g, 27.2 mmol, 1 equiv.); the mixture was heated at reflux under nitrogen atmosphere for 24 hours. After cooling to room temperature, methanol (20 mL) was then added. The residue was concentrated and separated by flash chromatography on silica column (elution with *n*-heptane/ethyl acetate: 80/20 → *n*-heptane/ethyl acetate: 0/100) to generate pure product **M16** as a yellow oil in 71% yield.

IR ν cm^{-1}: 2957, 1739, 1692, 1437, 1364, 1208, 1040, 596.

^{1}H NMR (400 MHz, $CDCl_3$) δ ppm: 0.91 (t, *J* = 7.2 Hz, 3H, $NHCH_2CH_2CH_2CH_3$), 1.34 (sext, *J* = 15.2, 7.5 Hz, 2H, $NHCH_2CH_2CH_2CH_3$), 1.52 (quint, *J* = 14.2, 7.2 Hz, 2H, $NHCH_2CH_2CH_2CH_3$), 2.33-2.45 (m, 2H, CH_2CH_2CH), 2.59-2.70 (m, 1H, CH_2CH_2CH), 2.82-2.95 (m, 1H, CH_2CH_2CH), 3.29 (dd, *J* = 7.5, 2.1 Hz, 2H, $NHCH_2CH_2CH_2CH_3$), 5.02 (dd, *J* = 7.7, 3.4 Hz, 1H, CH_2CH_2CH), 8.04 (br s, 1H, N*H*).

^{13}C NMR (100 MHz, $CDCl_3$) δ ppm: 13.6 (CH_3), 19.9 (CH_2), 22.7 (CH_2), 31.5 (CH_2), 31.7 (CH_2), 39.8 (CH_2), 46.4 (CH), 117.6 (C), 150.9 (C), 174.7 (C).

Anal. calcd for $\mathbf{C_{10}H_{15}N_3O_2}$ (209.25 g/mol): C, 57.40; H, 7.23; N, 20.08%. Found: C, 57.78; H, 7.06; N, 20.43%.

LogP: -1.03 ± 0.62.

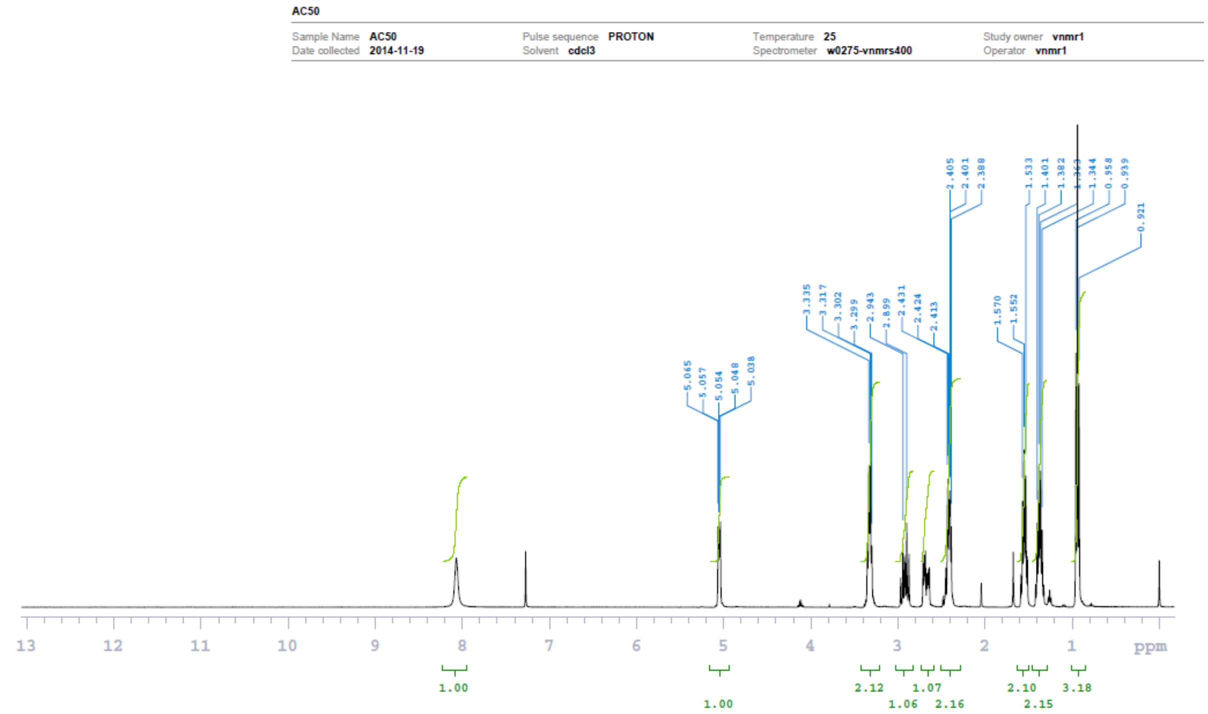
AC50
Sample Name AC50
Date collected 2014-11-19
Pulse sequence PROTON
Solvent cdcl3
Temperature 25
Spectrometer w0275-vnmrs400
Study owner vnmr1
Operator vnmr1
5.065
5.057
5.054
5.048
5.038
3.335
3.317
3.302
3.299
2.943
2.899
2.431
2.424
2.413
2.405
2.401
2.388
1.570
1.552
1.533
1.401
1.382
1.344
0.958
0.939
0.921
1.00
1.00
2.12
1.06
1.07
2.16
2.10
2.15
3.18
ppm

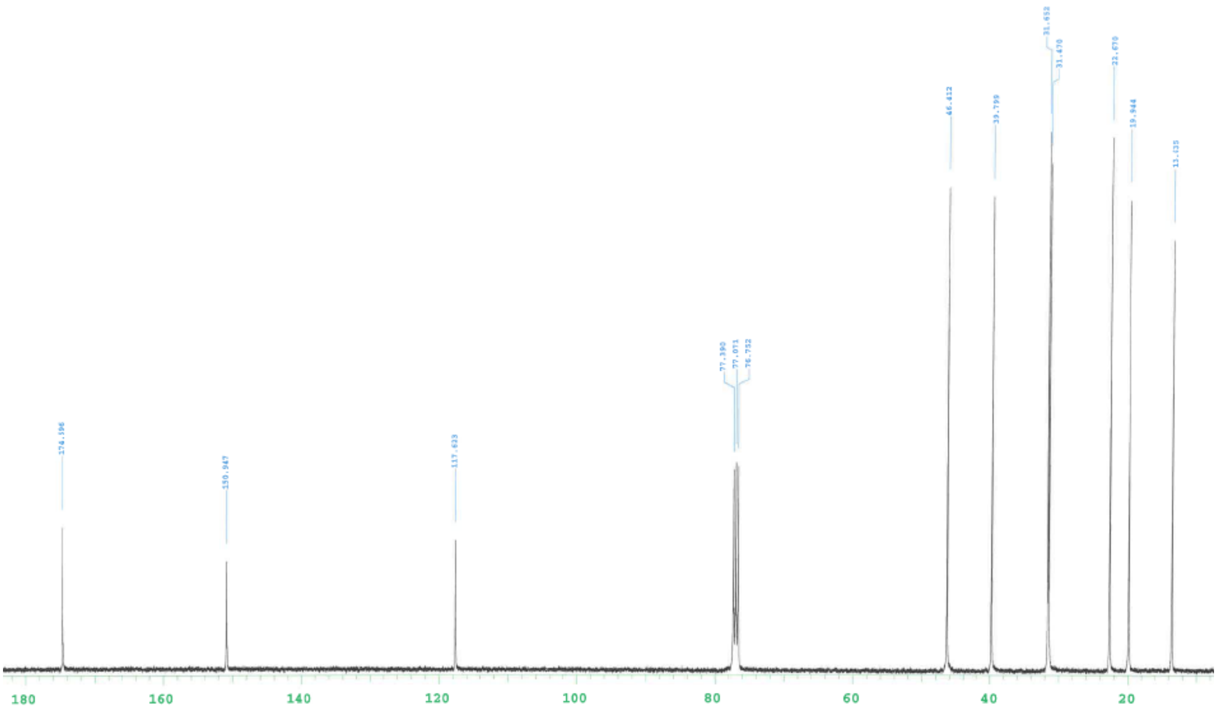
174.196
150.947
117.633
77.390
77.071
76.752
46.412
39.799
31.652
31.470
22.670
19.944
13.435

N^1-butyl-N^2,N^2-dimethyl-5-oxopyrrolidine-1,2-dicarboxamide **(M17)**

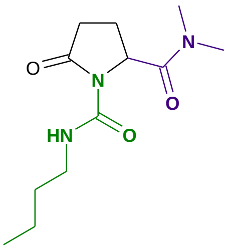

The general procedure was followed using *N*,*N*-dimethyl-5-oxoprolinamide[v] (5.0 g, 32.0 mmol, 1 equiv.) in toluene (20mL) and *n*-butyl isocyanate (3.17 g, 32.0 mmol, 1 equiv.); the mixture was heated at reflux under nitrogen atmosphere for 24 hours. After cooling to room temperature, methanol (20 mL) was added. The residue was concentrated and separated by flash chromatography on silica column (elution with *n*-heptane/ethyl acetate: 80/20 → *n*-heptane/ethyl acetate: 0/100) to generate pure product **M17** as a white solid in 45% yield, mp 48-49 °C.

IR ν cm^{-1}: 2957, 1739, 1692, 1437, 1364, 1208, 1040, 596.

^{1}H NMR (400 MHz, $CDCl_3$) δ ppm: 0.88 (dt, *J* = 7.2, 1.2 Hz, 3H, $NHCH_2CH_2CH_2CH_3$), 1.32 (m, *J* = 15.0, 7.4 Hz, 2H, $NHCH_2CH_2CH_2CH_3$), 1.48 (quint, *J* = 14.2, 7.5 Hz, 2H, $NHCH_2CH_2CH_2CH_3$), 1.85-1.93 (m, 1H, CH_2CH_2CH), 2.13-2.26 (m, 1H, CH_2CH_2CH), 2.48 (dd, *J* = 17.5, 8.2 Hz, 1H, CH_2CH_2CH), 2.85 (quint, *J* = 18.6, 7.8 Hz, 1H, CH_2CH_2CH), 2.95 (s, 3H, $N(CH_3)_2$, 3.10 (s, 3H, $N(CH_3)_2$, 3.12-3.22 (m, 1H, $CH_2CH_2CH_2CH_3$), 3.23-3.33 (m, 1H, $CH_2CH_2CH_2CH_3$), 5.12 (d, *J* = 9.2 Hz, 1H, CH_2CH_2CH), 8.36 (br s, 1H, N*H*).

^{13}C NMR (100 MHz, $CDCl_3$) δ ppm: 13.7 (CH_3), 20.0 (CH_2), 21.3 (CH_2), 31.6 (CH_2), 32.0 (CH_2), 36.0 (CH_3), 36.8 (CH_3), 39.6 (CH2), 59.2 (CH), 152.6 (C), 171.1 (C), 177.0 (C).

Anal. calcd for **$C_{12}H_{21}N_3O_3$** (255.32 g/mol): C, 56.45; H, 8.29; N, 16.46%. Found: C, 56.73; H, 8.36; N, 16.24%.

LogP: -1.63 ± 0.63.

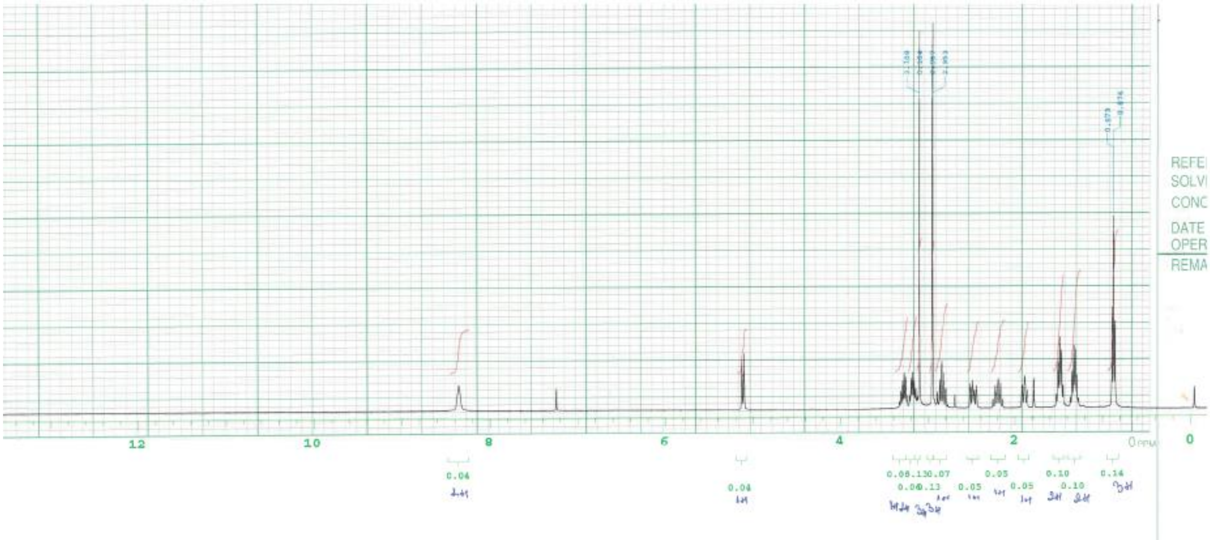

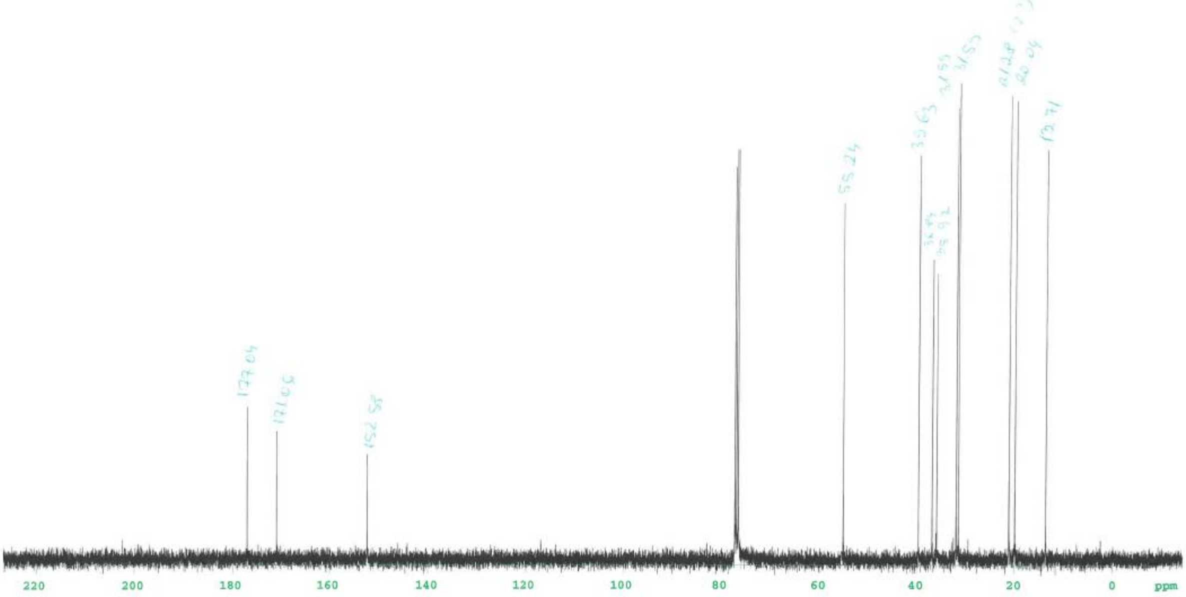

Dodecyl 1-[(butylamino)carbonyl]-5-oxoprolinate **(M18)**

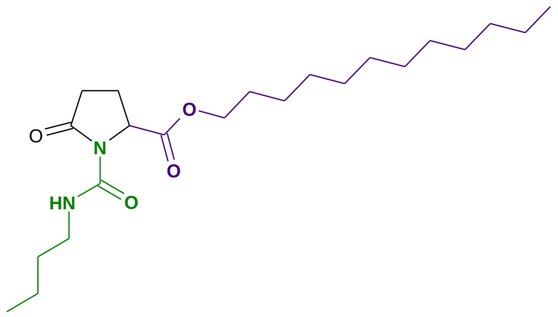

The general procedure was followed using dodecyl 5-oxoprolinate[vi] (5.0 g, 16.8 mmol, 1 equiv.) in toluene (20mL) and *n*-butyl isocyanate (1.99 g, 20.0 mmol, 1.2 equiv.) and the mixture was heated at reflux under nitrogen atmosphere for 48 hours. After cooling to room temperature, methanol (20 mL) was added. The residue was concentrated and separated by flash chromatography on silica column (elution with *n*-heptane/ethyl acetate: 80/20 → *n*-heptane/ethyl acetate: 0/100) to generate pure product **M18** as a yellow oil in 78% yield, R_f (EtOAc/*n*-heptane 50/50) = 0.7.

IR ν cm^{-1}: 3317, 2960, 1717, 1539, 1459, 1368, 1255, 1193, 734, 595.

^{1}H NMR (400 MHz, $CDCl_3$) δ ppm: 0.88 (t, *J* = 6.7 Hz, 3H, CH_3), 0.93 (t, *J* = 7.4 Hz 3H, CH_3), 1.26 (s, 18H, 9CH_2), 1.30-1.41 (m, 4H, 2CH_2), 1.50-1.57 (m, 2H, CH_2), 2.00-2.08 (m, 1H, CH_2CH_2CH), 2.28-2.39 (m, 1H, CH_2CH_2CH), 2.52-2.60 (m, 1H, CH_2CH_2CH), 2.68-2.78 (m, 1H, CH_2CH_2CH), 3.24-3.34 (m, 2H, $NHCH_2CH_2CH_2CH_3$), 4.12-4.22 (m, 2H, OCH_2), 4.79 (dd, *J* = 9.8, 2.7 Hz, 1H, CH_2CH_2CH), 8.28 (br s, 1H, N*H*).

^{13}C NMR (100 MHz, $CDCl_3$) δ ppm: 13.7 (CH_3), 14.0 (CH_3), 20.0 (CH_2), 21.3 (CH_2), 22.6 (CH_2), 25.7 (CH_2), 28.4 (CH_2), 29.2 (CH_2), 29.3 (CH_2), 29.4 (CH_2), 29.5 (CH_2), 29.6 (2CH_2), 31.6 (CH_2), 31.8 (CH_2), 31.9 (CH_2), 39.6 (CH_2), 58.1 (CH), 65.7 (CH_2), 152.1 (C), 171.5 (C), 176.3 (C).

Anal. calcd for $\mathbf{C_{22}H_{40}N_2O_4}$ (396.58 g/mol): C, 66.63; H, 10.17; N, 7.06%. Found: C, 66.98; H, 10.30; N, 7.32%.

LogP: 5.14± 0.62.

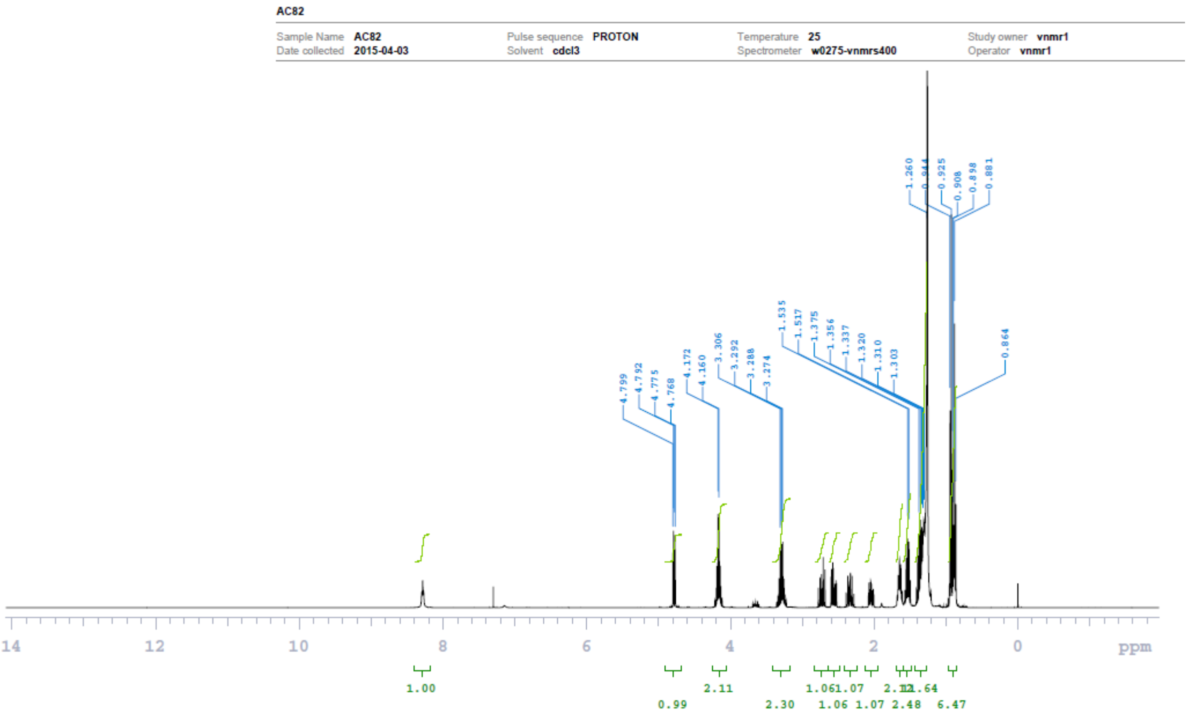
AC82
Sample Name AC82
Date collected 2015-04-03
Pulse sequence PROTON
Solvent cdcl3
Temperature 25
Spectrometer w0275-vnmrs400
Study owner vnmr1
Operator vnmr1
4.799
4.792
4.775
4.768
4.172
4.160
3.306
3.292
3.288
3.274
1.535
1.517
1.375
1.356
1.337
1.320
1.310
1.303
1.260
0.944
0.925
0.908
0.898
0.881
0.864
14
12
10
8
6
4
2
0
ppm
1.00
0.99
2.11
2.30
1.06
1.06
1.07
1.07
2.48
6.47

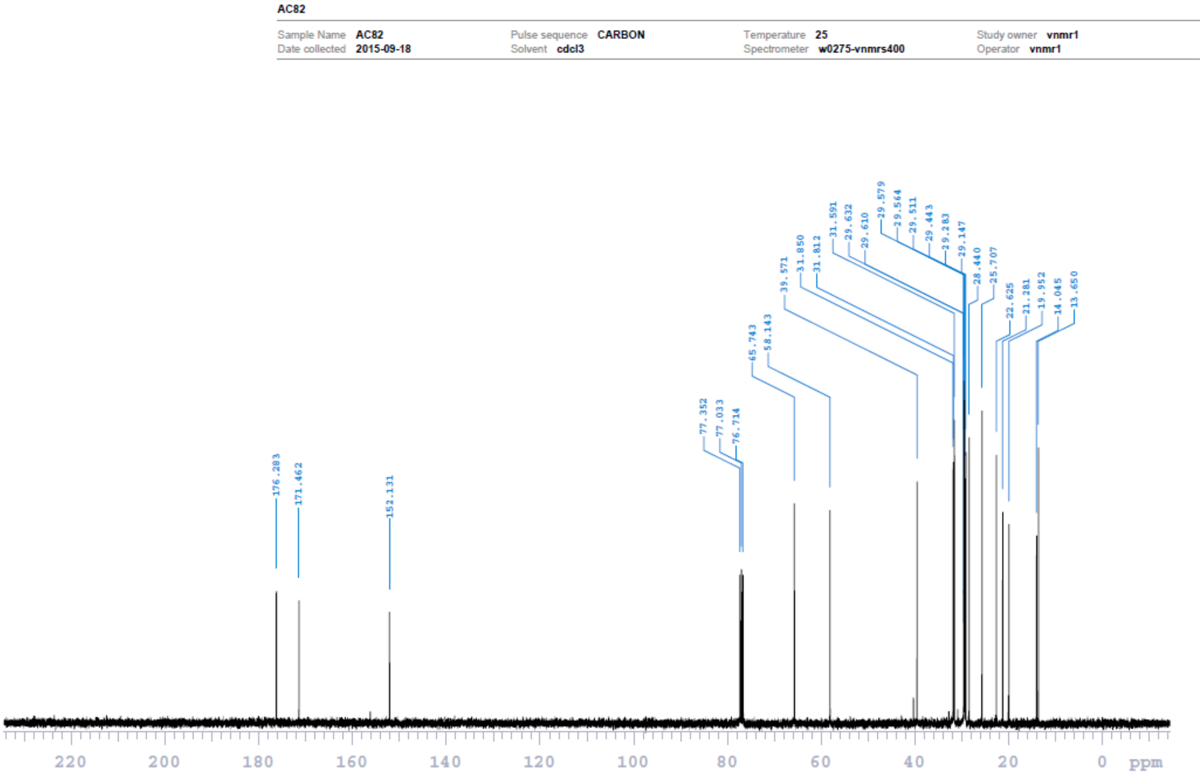
AC82
Sample Name AC82
Date collected 2015-09-18
Pulse sequence CARBON
Solvent cdcl3
Temperature 25
Spectrometer w0275-vnmrs400
Study owner vnmr1
Operator vnmr1
176.283
171.462
152.131
77.352
77.033
76.714
65.743
58.143
39.571
31.850
31.812
31.591
29.632
29.610
29.579
29.564
29.511
29.443
29.283
29.147
28.440
25.707
22.625
21.281
19.952
14.045
13.650
220
200
180
160
140
120
100
80
60
40
20
0
ppm

5-Methoxycarbonylmethylene-pyrrolidine-2-carboxylic acid methyl ester (**M21**)

The synthesis of **M21** is presented in Reaction 9 and was realized starting from the pyroglutamic acid and was described previously by our team.[3]

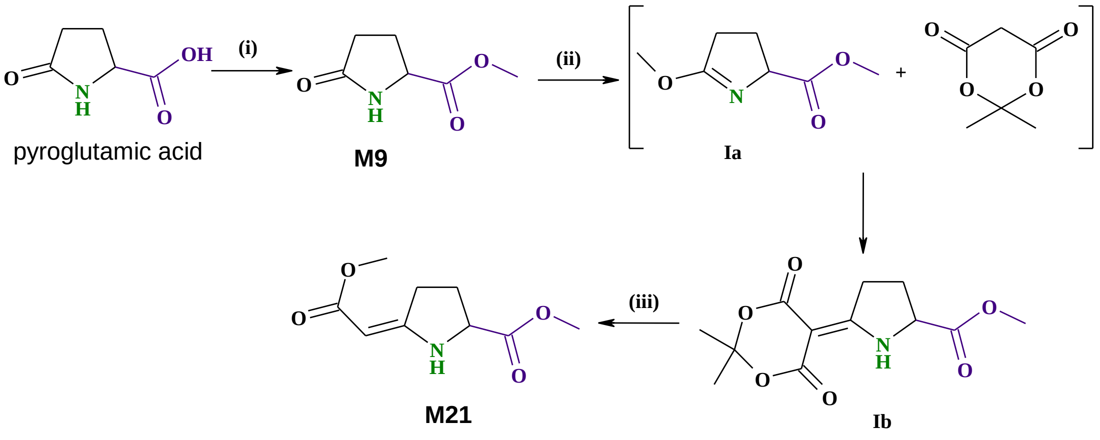

Reaction 9. Reagents and conditions: *(i) MeOH, $CHCl_3$, 3Å molecular sieves, $ZrCl_4$, reflux, 48h; (ii) (a) $(MeO)_2SO_2$, 60°C, 12h (b) TEA, rt, 5h; (iii) (a) MeONa, MeOH, reflux, 24h (b) HCl, rt;*[3]

Methyl 2-oxo-1,3-oxazolidine-4-carboxylate (**M22**) *and* *2-Oxo-1,3-oxazolidine-4-carboxylic acid* (**M27**)

The synthesis of **M22** was realized following a two-steps procedure, described in Reaction 10. The first step was the synthesis of 2-oxo-1,3-oxazolidine-4-carboxylic acid **M27** starting from commercially available serine by using the classical conditions described in the literature.[4] Carboxylic acid **M22** was then esterified in a mixture of methanol and chloroform at reflux in presence of thionyl chloride and catalytic amount of DMF. Final product **M22** was obtained as pure compound with the same physico-chemical properties as described in the literature.[5]

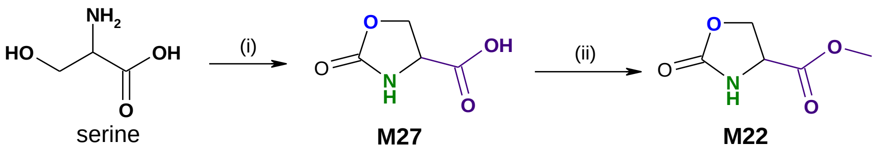

Reaction 10. *Reagents and conditions: (i) phenyl chloroformiate, NaOH, toluene/H_2O; (ii) thionyl chloride, DMF, MeOH/$CHCl_3$, reflux.*

<u>*Methyl 2-oxo-1,3-thiazolidine-4-carboxylate*</u> **(M23)**

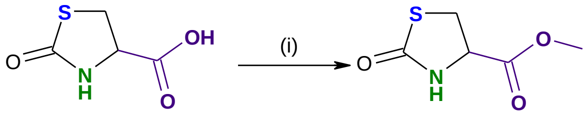

Reaction 11. *Reagents and conditions: (i) thionyl chloride, DMF, MeOH/CHCl*$_3$*, reflux, 51%.*

2-Oxo-1,3-thiazolidine-4-carboxylic acid (**M28**) (1 equiv.) was added to a solution of thionyl chloride (1.4 equiv.) and treated with catalytic amount of *N,N'*-dimethylformamide (DMF, 100 µL) in MeOH (50 mL) at 80 °C (Reaction 11). The mixture was heated during 24 h under nitrogen atmosphere, then concentrated *in vacuo*. Dichloromethane was added to the crude and extracted with water. The organic layers were dried on $MgSO_4$ and concentrated *in vacuo* to provide the pure compound **M23** in 51% yield with the same physico-chimical properties as described in the literature.[11]

^{1}H NMR (400 MHz, $CDCl_3$) δ ppm: 3.61 (dd, J = 11.3, 4.9 Hz, 1H, SC*H*$_2$CH), 3.72 (dd, J = 11.3, 3.2 Hz, 1H, SC*H*$_2$CH), 3.83 (s, 3H, OC*H*$_3$), 4.48 (dd, J = 8.3, 4.7 Hz, 1H, SCH$_2$C*H*), 6.88 (br s, 1H, NH).

^{13}C NMR (100 MHz, $CDCl_3$) δ ppm: 31.8 (CH_2), 53.2 (CH_3), 56.1 (CH), 170.7 (C), 174.8 (C).
LogP: -0.65 ± 0.71.

<u>*1-tert-Butylcarbamoyl-5-oxo-pyrrolidine-2-carboxylic acid*</u> (**M24**)

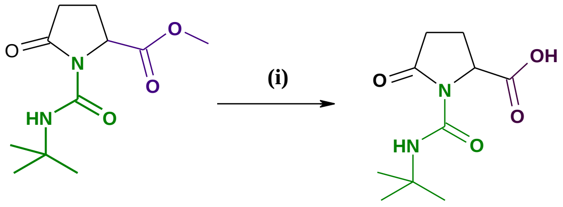

Reaction 12. *Reagents and conditions: (i) 1.2 equiv. NaOH, H*$_2$*O, reflux, 4h, 47%.*

1-*tert*-Butylcarbamoyl-5-oxo-pyrrolidine-2-carboxylic acid methyl ester (1 equiv.) was stirred at reflux with a 2N aqueous solution of sodium hydroxide (1.2 equiv.) for 4 hours (Reaction 12). After complete solubilization and cooling the medium at rt, concentrated HCl was added till a white precipitate formed. The precipitate was washed with water and dried to provide pure

carboxylic acid **M24** as a white solid in 47% yield; mp (H_2O) 110-113°C; R_f (EtOAc/*n*-heptane 1/1) = 0.56.

^{1}H NMR (400 MHz, $CDCl_3$) δ ppm: 1.36 (s, 9H, C(CH_3)$_3$), 2.16-2.34 (m, 2H, CH_2CH_2CH), 2.51-2.60 (m, 1H, C$H_2$$CH_2$CH), 2.70-2.81 (m, 1H, C$H_2$$CH_2$CH), 4.78 (dd, J = 9.4, 2.7 Hz, 1H, CH_2CH_2CH), 8.38 (s, 1H, NH), 8.38 (br s, 1H, $CO_2$$H$).

^{13}C NMR (100 MHz, $CDCl_3$) δ ppm: 20.7 (CH_2), 28.8 (3CH_3), 31.9 (CH_2), 51.3 (C), 57.9 (CH), 151.4 (C), 175.1 (C), 176.4 (C).

Anal. calcd for **$C_{10}H_{16}N_2O_4$** (228.25 g/mol): C, 52.62; H, 7.07; N, 12.27%. Found: C, 52.40; H, 7.33; N, 12.28%.

LogP: -1.53 ± 0.62.

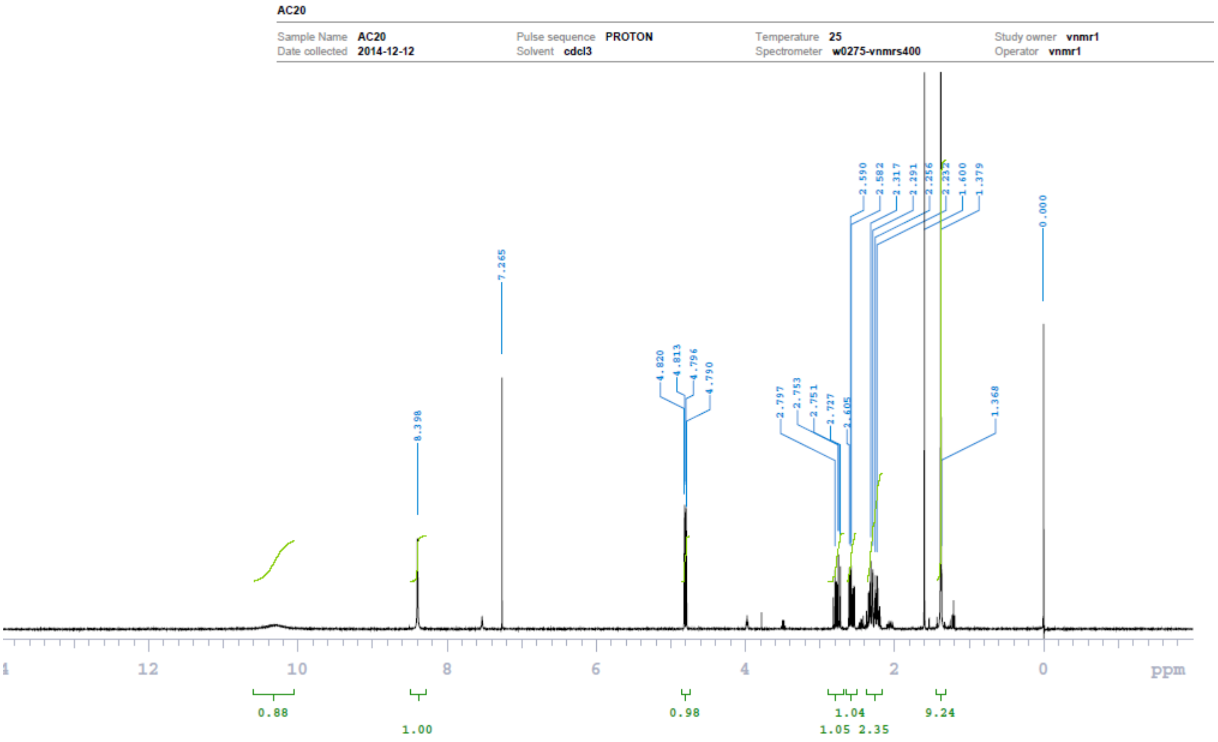

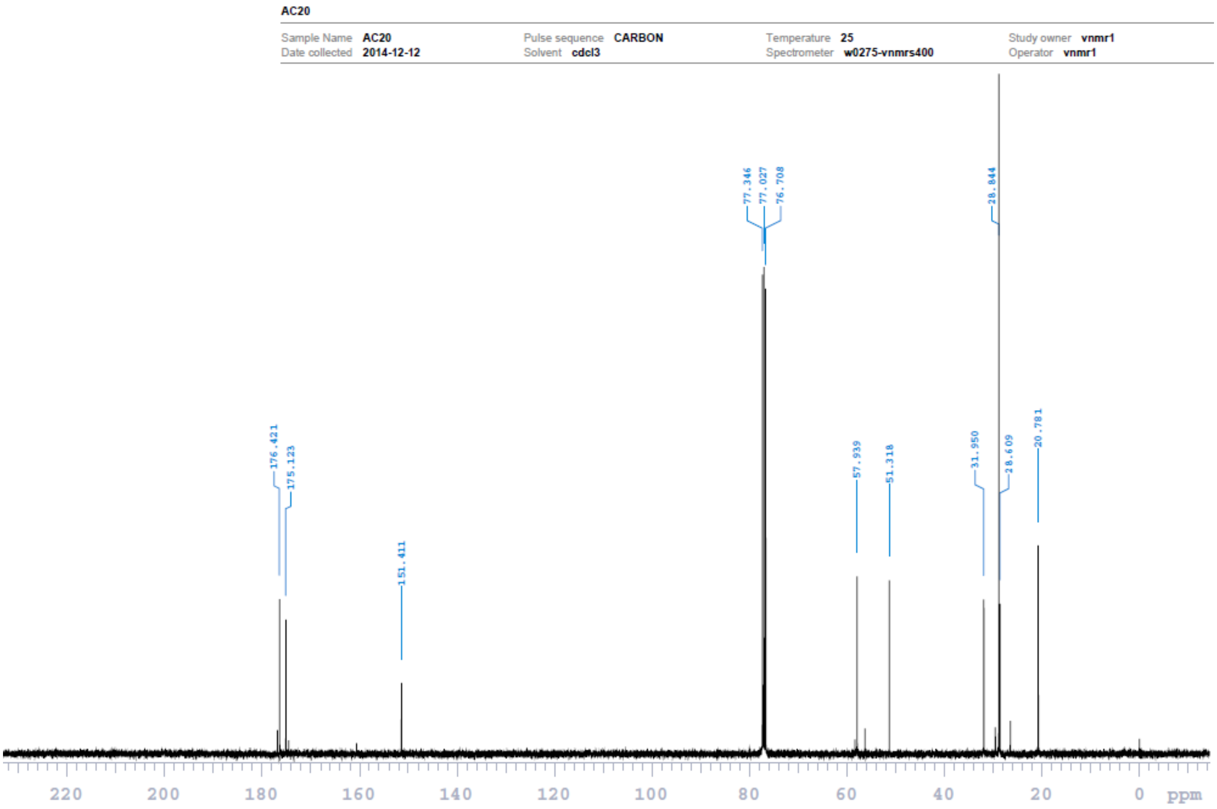

Dodecyl 1-[(dodecylamino)carbonyl]-5-oxoprolinate **(M25)**

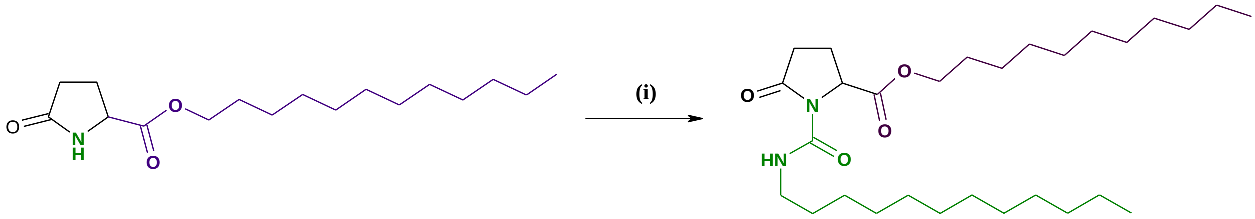

M25

Reaction 13. *Reagents and conditions: (i) 1.0 eq. n-dodecyl isocyanate, toluene, reflux, 24h.*

Dodecyl 5-oxoprolinate (3.0 g, 10 mmol, 1 equiv.) in toluene (20mL) was treated with *n*-dodecyl isocyanate (2.13 g, 10 mmol, 1 equiv.) and the mixture was heated at reflux under nitrogen atmosphere for 24 hours (Reaction 13). After cooling to room temperature, methanol (20 mL) was added. The residue was concentrated and separated by flash chromatography on silica pre-packed column (elution with *n*-heptane/ethyl acetate: 80/20 → *n*-heptane/ethyl acetate: 0/100) to generate pure product **M25** as a white solid in 75% yield, R_f (EtOAc/*n*-heptane 50/50) = 0.7.

IR ν cm^{-1}: 3334, 2919, 1721, 1686, 1537, 1465, 1248, 1225, 721, 634, 597.

^{1}H NMR (400 MHz, $CDCl_3$) δ ppm: 0.88 (t, *J* = 6.3 Hz, 6H, $2CH_2(CH_2)_9CH_2C\mathit{H}_3$), 1.26 (s large, 36H, $2CH_2(C\mathit{H}_2)_9CH_2CH_3$), 1.52-1.57 (m, 4H, $2CH_2(CH_2)_9C\mathit{H}_2CH_3$), 2.01-2.07 (m, 1H, $CH_2C\mathit{H}_2CH$), 2.28-2.39 (m, 1H, $CH_2C\mathit{H}_2CH$), 2.52-2.60 (m, 1H, $C\mathit{H}_2CH_2CH$), 2.69-2.78 (m, 1H, $C\mathit{H}_2CH_2CH$), 3.22-3.31 (m, 2H, $NHC\mathit{H}_2(CH_2)_9CH_2CH_3$), 4.14-4.19 (m, 2H, $OC\mathit{H}_2(CH_2)_9CH_2CH_3$), 4.78 (dd, *J* = 9.2, 2.0 Hz, 1H, $CH_2CH_2C\mathit{H}$), 8.28 (br s, 1H, $N\mathit{H}$).

^{13}C NMR (100 MHz, $CDCl_3$) δ ppm: 13.5 ($2CH_3$), 20.7 (CH_2), 22.1 ($2CH_2$), 25.2 (CH_2), 26.3 (CH_2), 27.9 (CH_2), 28.6 (CH_2), 28.7 ($4CH_2$), 28.9 ($2CH_2$), 29.0 ($7CH_2$), 31.3 ($2CH_2$), 39.4 (CH_2), 57.6 (CH), 65.2 (CH_2), 151.6 (C), 170.9 (C), 175.7 (C).

Anal. calcd for **$C_{30}H_{56}N_2O_4$** (508.79 g/mol): C, 70.82; H, 11.09; N, 5.51%. Found: C, 71.11; H, 11.42; N, 5.82%.

LogP: 9.39 ± 0.62.

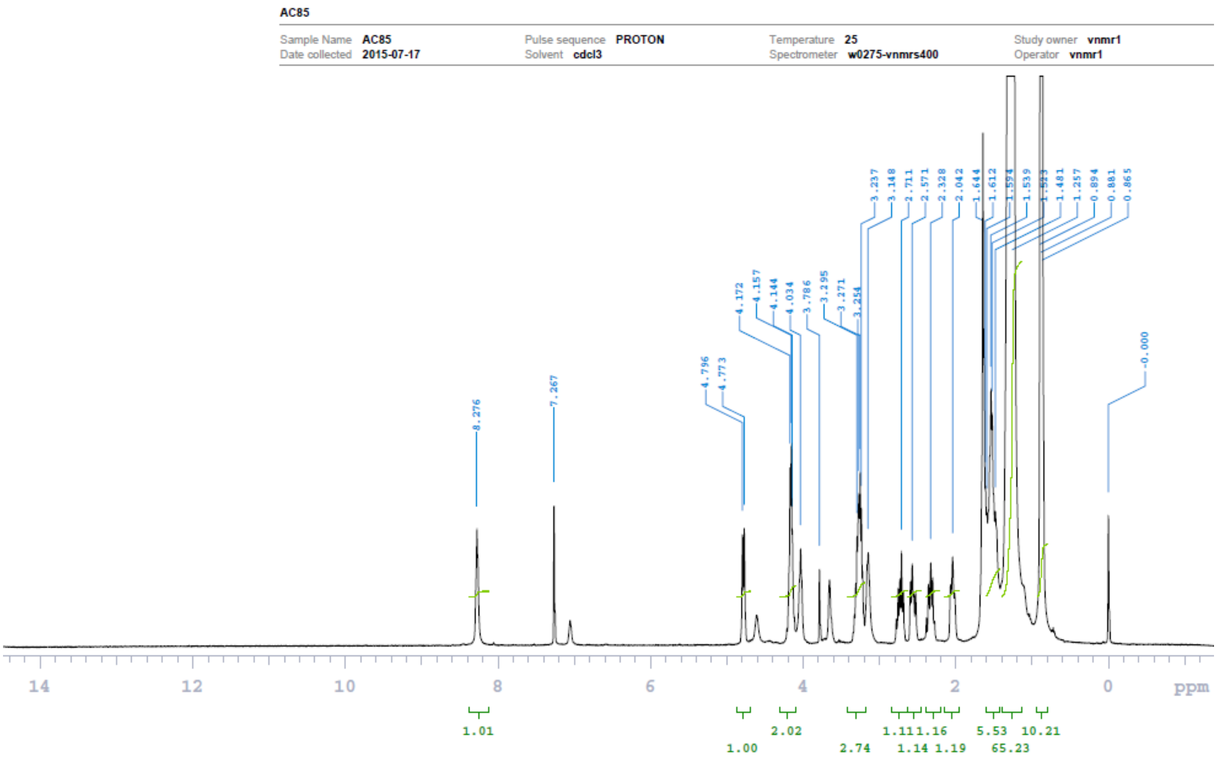

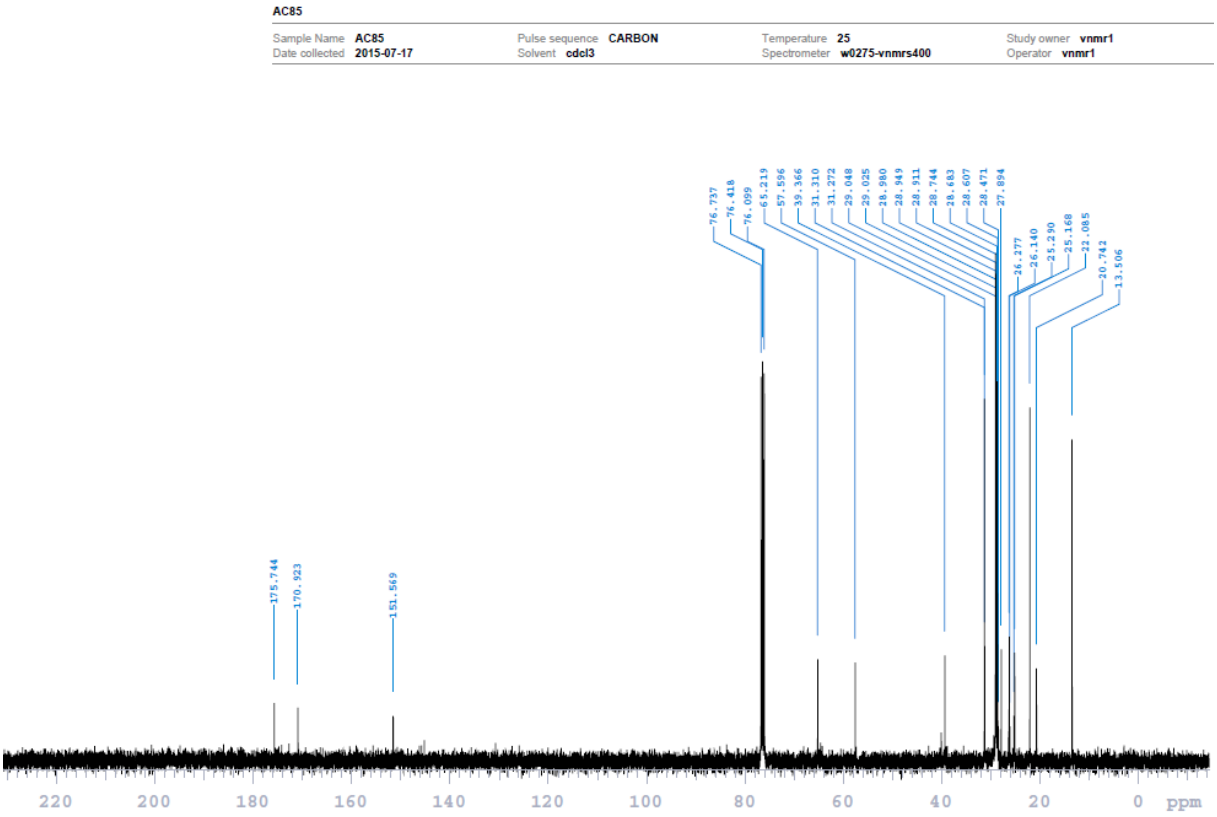

Methyl 6-oxopiperidine-2-carboxylate (**M26**)

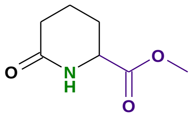

6-Oxopiperidine-2-carboxylic acid (1 equiv.) was added to a solution of thionyl chloride (1.4 equiv.) and treated with catalytical amount of *N*,*N*-dimethylformamide (DMF, 100 µL) in MeOH (50 mL) at 80 °C. The mixture was heated during 24 h under nitrogen atmosphere. The mixture was concentrated and dichloromethane was added. The organic layers were dried on $MgSO_4$ and concentrated in vacuum to provide the pure compound **M26** in 51 % yield with the same properties as described in the literature. [vii]

^{1}H NMR (400 MHz, $CDCl_3$) δ ppm: 1.74-1.95 (m, 4H, $CH_2CH_2CH_2CH$), 2.30-2.47 (m, 2H, $CH_2CH_2CH_2CH$), 3.79 (s, 3H, OCH_3), 4.10 (t, *J* = 6.4 Hz, 1H, $CH_2CH_2CH_2CH$), 6.23 (br s, 1H, N*H*).

^{13}C NMR (100 MHz, $CDCl_3$) δ ppm: 19.5 (CH_2), 25.3 (CH_2), 31.0 (CH_2), 52.7 (CH_3), 54.7 (CH), 171.2 (C), 171.5 (C).

Molecular formula: **$C_7H_{11}NO_3$** (157.17). **LogP**: -1.36 ± 0.28.

Methyl 1-[(butylamino)carbonyl]-6-oxopiperidine-2-carboxylate (**M29**)

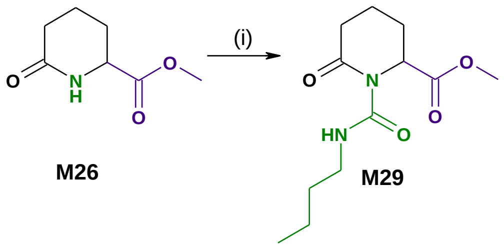

Reaction 14. *Reagents and conditions: (i) 1.2 equiv. n-C_4H_9NCO, toluene, reflux, 24 h, inert atmosphere.*

N-butyl isocyanate (1.89 g, 19.0 mmol, 1 equiv.) was added to methyl 6-oxopiperidine-2-carboxylate **M26** (3.0 g, 19.0 mmol, 1 equiv.) in toluene (20mL) and the resulting mixture was heated at reflux under nitrogen atmosphere for 24 hours (Reaction 14). After cooling to room temperature, methanol (20 mL) was added. The residue was concentrated and separated by flash chromatography on silica pre-packed column (elution with *n*-heptane/ethyl acetate: 80/20 → *n*-heptane/ethyl acetate: 0/100) to generate pure product **M29** as a yellow oil in 44 % yield.

IR ν cm^{-1}: 3280, 1745, 1700, 1655, 1525, 1390, 1202, 1091, 581.

^{1}H NMR (400 MHz, $CDCl_3$) δ ppm: 0.92 (t, *J* = 7.4 Hz, 3H, $NHCH_2CH_2CH_2CH_3$), 1.31-1.41 (m, 2H, $NHCH_2CH_2CH_2CH_3$), 1.48-1.57 (m, 2H, $NHCH_2CH_2CH_2CH_3$), 1.65-1.83 (m, 2H, $CH_2CH_2CH_2CH$), 1.92-2.02 (m, 1H, $CH_2CH_2CH_2CH$), 2.20-2.29 (m, 1H, $CH_2CH_2CH_2CH$), 2.45-2.56 (m, 1H, $CH_2CH_2CH_2CH$), 2.58-2.67 (m, 1H, $CH_2CH_2CH_2CH$), 3.21-3.35 (m, 2H, $NHCH_2CH_2CH_2CH_3$) 3.76 (s, 3H, OCH_3), 5.10 (dd, *J* = 6.0, 3.5 Hz, 1H, $CH_2CH_2CH_2CH$), 9.33 (br s, 1H, NH).

^{13}C NMR (100 MHz, $CDCl_3$) δ ppm: 13.7 (CH_3), 17.6 (CH_2), 20.0 (CH_2), 25.4 (CH_2), 31.4 (CH_2), 33.7 (CH_2), 40.2 (CH_2), 52.5 (CH_3), 55.8 (CH), 154.6 (C), 172.0 (C), 174.1 (C).

Anal. calcd for **$C_{12}H_{20}N_2O_4$** (256.30 g/mol): C, 56.24; H, 7.87; N, 10.93 %. Found: C, 56.28; H, 7.99; N, 11.32%.

LogP: -0.14 ± 0.62.

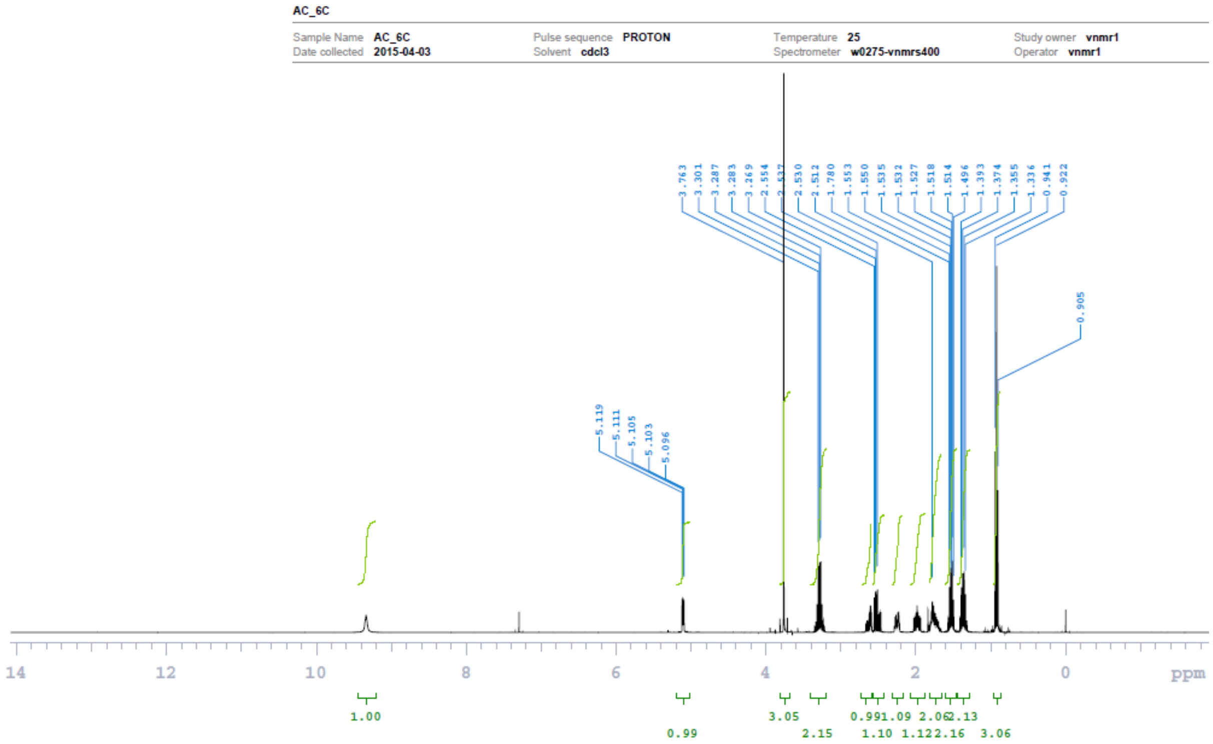
AC_6C
Sample Name AC_6C
Date collected 2015-04-03
Pulse sequence PROTON
Solvent cdcl3
Temperature 25
Spectrometer w0275-vnmrs400
Study owner vnmr1
Operator vnmr1
ppm

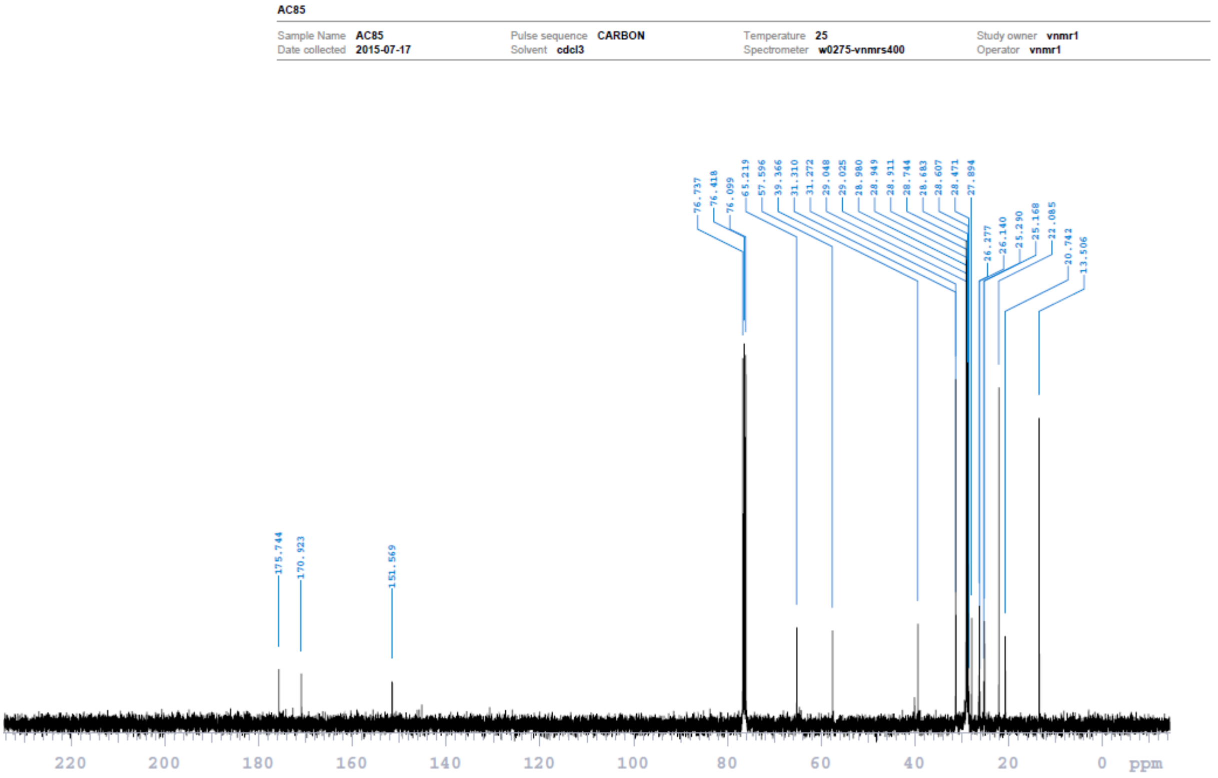
AC85
Sample Name AC85
Date collected 2015-07-17
Pulse sequence CARBON
Solvent cdcl3
Temperature 25
Spectrometer w0275-vnmrs400
Study owner vnmr1
Operator vnmr1
ppm

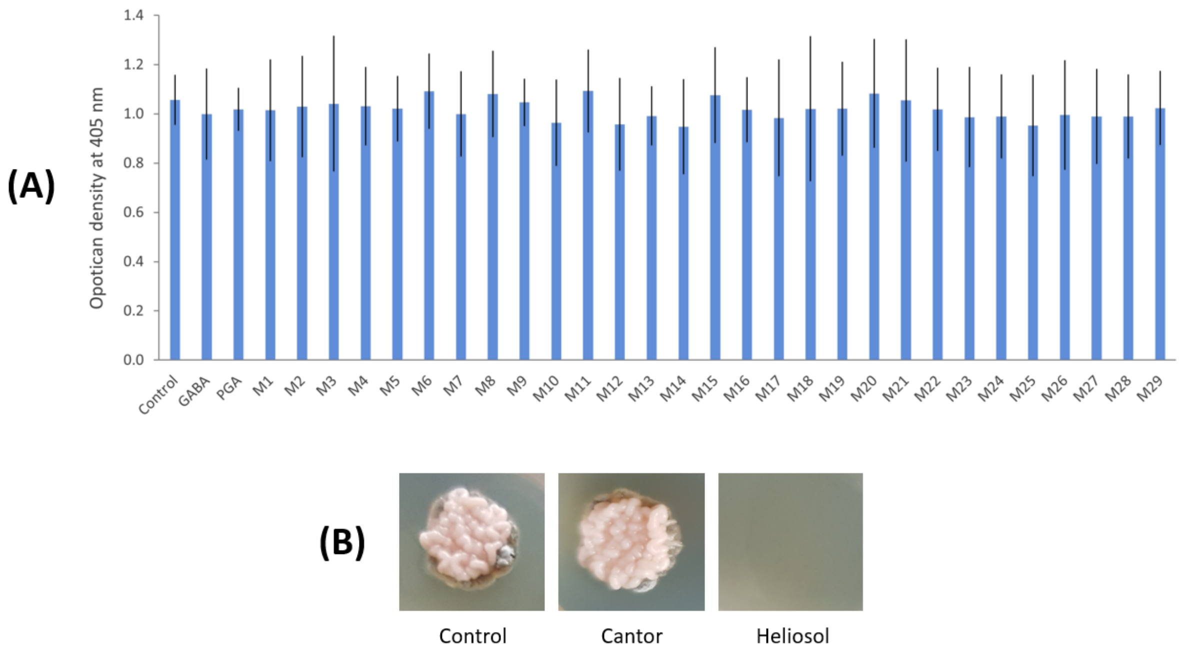

Supplementary Figure S2. *In vitro* antifungal activity of the compounds tested in the present study against *Zymoseptoria tritici* (strain T02596). **A**, *in vitro* antifungal effect at 5 mM of γ-aminobutyric acid (GABA), pyroglutamic acid (PGA), M1 (derived from PGA), and 28 molecules functionalized from M1, assessed on glucose peptone liquid medium using 96-well miroplates. **B**, Illustration of the *in vitro* antifungal assay performed with the wetting agents Heliosol (at 0.2%) and Cantor (at 0.15%) on Petri dishes amended with potato dextrose agar (PDA) solid medium. Three biological replicates were used for each molecule and the standard deviations (bar errors) were calculated from the mean of these three biological replicates.

Supplementary Table S1. Primers used in quantitative reverse-transcription polymerase chain reaction (RT-qPCR) assays and their calculated efficacies.

Gene	Primer sequences 5’-3’	Accession number	Efficacy/template
ACT (*actin, housekeeping gene*)	AACCTTCAGTTGCCCAGCAA<br>TGTTCGACCGCTGGCATAC	AB181991	98.9 %
TUB (*beta tubulin, housekeeping gene*)	GGAGTACCCTGACCGAATGATG<br>AACGACGGTGTCTGAGACCTTT	U76895	104.3 %
LOX (*lipoxygenase*)	GGGCACCAAGGAGTACAAGGA<br>GCTCGTGATGGTGTGGATGA	U32428	103.7 %
PAL (*phenylalanine Ammonia-lyase*)	CCCCCATTGGTGTCTCCAT<br>ACTGCGCGAACATCAGCTT	AY005474	102.9 %
POX2 (*peroxidase*)	AGCCACAGCCACAACCAGATA<br>AGAAATGCGAGAGGCAGAACC	X85228	102.3 %
PR1 (*pathogenesis related*)	CATGCACCTTCGTATGCCTAACT<br>TGGCTTATTACGGCATTCCTTT	HQ541964	105 %

References

1. Cauliez, P.; Fasseur, D.; Couturier, D.; Rigo, B.; Kolocouris, A. *J. Heterocycl. Chem.* **1996**, *33*, 1233.
2. Rigo, B.; Erb, B.; Ghammarti, S. E.; Gautret, P.; Couturier, D. *J. Heterocycl. Chem.* **1995**, *32*, 1599
3. Rigo, B.; Lespagnol, C.; Pauly, M. *J. Heterocycl. Chem.* **1988**, *25*, 49.
4. Rigo, B. ; Lespagnol, C. ; Pauly, M. *Tetrahedron Lett.*, **1986**, *27*, 347.
5. Gang, F.; Zhu, F.; Li, X.; Wei, J.; Wu, W.; Zhang, J. *Bioorg. Med. Chem.* **2018**, I, 4644.
6. Shimada, H.; Yamazaki, K.; Ueno, M.; Meguro, K. J. Am. Oil Chem. Soc. 1980, 57, 318.
7. Fasseur, D.; Rigo, B.; Leduc, C.; Cauliez, P.; Defretin, S. *J. Heterocycl. Chem*. **1994**, *31*, 829
8. Seki, M.; Hatsuda, M.; Mori, Y.; Yoshida, S.; Yamada, S.; Shimizu, T. *Chem. Eur. J.*, **2004**, *10*, 6102.
9. Xue, F.; Seto, C. T. *Org. Lett*. **2010**, *12*, 1936.
10. Seki, M.; Hatsuda, M.; Mori, Y.; Yoshida, S.; Yamada, S.; Shimizu, T. *Chem. Eur. J.*, **2004**, *10*, 6102.
11. Kumar, K.S.A.; Misra, A.; Siddiqi, T.I.; Srivastava, S.; Jain, M.; Bhatta, R.S.; Barthwal, M.; Dikshit, M. *Eur. J. Med. Chem*. **2014**, *81*, 456.
